# APOL1-mediated monovalent cation transport contributes to APOL1-mediated podocytopathy in kidney disease

**DOI:** 10.1172/JCI172262

**Published:** 2024-01-16

**Authors:** Somenath Datta, Brett M. Antonio, Nathan H. Zahler, Jonathan W. Theile, Doug Krafte, Hengtao Zhang, Paul B. Rosenberg, Alec B. Chaves, Deborah M. Muoio, Guofang Zhang, Daniel Silas, Guojie Li, Karen Soldano, Sarah Nystrom, Davis Ferreira, Sara E. Miller, James R. Bain, Michael J. Muehlbauer, Olga Ilkayeva, Thomas C. Becker, Hans-Ewald Hohmeier, Christopher B. Newgard, Opeyemi A. Olabisi

**Affiliations:** 1 Duke Molecular Physiology Institute and Sarah W. Stedman Nutrition and Metabolism Center, Duke University School of Medicine, Durham, North Carolina, USA.; 2 Duke University School of Medicine, Department of Medicine, Division of Nephrology, Durham, North Carolina, USA.; 3 OmniAb Inc., Durham, North Carolina, USA.; 4 Department of Medicine, Division of Cardiology, Duke University School of Medicine, Durham, North Carolina, USA.; 5 Department of Medicine, Division of Endocrinology, Metabolism, and Nutrition, Duke University School of Medicine, Durham, North Carolina, USA.; 6 Department of Pharmacology and Cancer Biology, Duke University Medical Center, Durham, North Carolina, USA.; 7 Department of Pathology, Duke University Medical Center, Durham, North Carolina, USA.

**Keywords:** Nephrology, Calcium signaling, Chronic kidney disease

## Abstract

Two coding variants of apolipoprotein L1 (*APOL1*), called G1 and G2, explain much of the excess risk of kidney disease in African Americans. While various cytotoxic phenotypes have been reported in experimental models, the proximal mechanism by which G1 and G2 cause kidney disease is poorly understood. Here, we leveraged 3 experimental models and a recently reported small molecule blocker of APOL1 protein, VX-147, to identify the upstream mechanism of G1-induced cytotoxicity. In HEK293 cells, we demonstrated that G1-mediated Na^+^ import/K^+^ efflux triggered activation of GPCR/IP3–mediated calcium release from the ER, impaired mitochondrial ATP production, and impaired translation, which were all reversed by VX-147. In human urine-derived podocyte-like epithelial cells (HUPECs), we demonstrated that G1 caused cytotoxicity that was again reversible by VX-147. Finally, in podocytes isolated from *APOL1 G1* transgenic mice, we showed that IFN-γ–mediated induction of G1 caused K^+^ efflux, activation of GPCR/IP3 signaling, and inhibition of translation, podocyte injury, and proteinuria, all reversed by VX-147. Together, these results establish APOL1-mediated Na^+^/K^+^ transport as the proximal driver of APOL1-mediated kidney disease.

## Abstract 2

APOL1-mediated monovalent cation transport is a proximal driver of APOL1-mediated podocytopathy in kidney disease.

## Graphical abstract

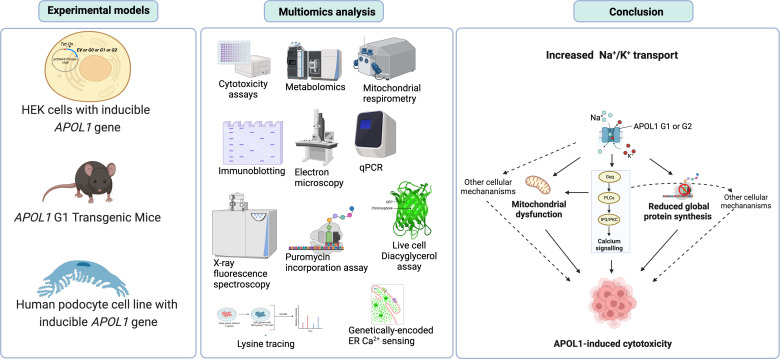

## Introduction

Two African apolipoprotein L1 (*APOL1*) variants, named G1 (p.S342G and p.I384M) and G2 (deletion of p.N388/Y389), appear to have evolved to confer protection against the African sleeping sickness parasite *Trypanosoma brucei rhodesiense*. However, G1 and G2 are also markedly associated with increased risk of a spectrum of chronic kidney diseases among African Americans. This discovery explains much of the excess risk of kidney disease among African Americans, who develop end-stage kidney failure at 3 to 4 times the rate of White Americans (1–7). The mechanism by which G1 and G2 — collectively called *APOL1* renal risk variants (RRVs) — cause kidney disease is poorly understood. Cell-based and transgenic animal models show that RRVs cause cellular injury and cell death, whereas the reference *APOL1* G0 is relatively nontoxic (8–12). The mechanism by which RRVs induce cytotoxicity in experimental models is believed to mirror the pathomechanism of APOL1-mediated kidney disease. Therefore, elucidation of the mechanism of RRV-induced cytotoxicity is a major priority.

Experimental models thus far have failed to produce a unified mechanism that explains the multiple cytotoxic phenotypes caused by RRVs (13–16). Prior studies show that in a planar lipid bilayer, APOL1 protein forms a cation-selective pore that is permeable to Na^+^ and K^+^ (17–19) and that cation flux drives trypanosome lysis by APOL1 (17, 19, 20). In a HEK cellular model with inducible expression of APOL1 G0, G1, or G2, APOL1 also forms cation pores in the plasma membrane (PM) of mammalian cells, but only G1 and G2 cause aberrant influx of Na^+^ and efflux of K^+^, leading to cellular swelling, activation of JNK and p38 MAPK, and cellular death (9, 21). Based on these results, we proposed that the cation pore function of RRVs is the proximal driver of cytotoxicity. While other investigators have confirmed our observation that RRVs cause K^+^ efflux and Na^+^ influx in mammalian cells, the role of this event as the proximal driver of RRV cytotoxicity has been contested (22, 23). Recently, Giovinazzo et al. (24) further extended the debate by reporting that in a planar lipid bilayer, all 3 APOL1 variants are also permeable to Ca^2+^ and that in cell-based models, expression of G1 and G2 caused a gradual increase in cytoplasmic Ca^2+^. They proposed that Ca^2+^ imported by APOL1 (along with Na^+^) drives cell death (24). Wu et al. also reported that induced expression of RRVs in human urinary podocytes led to increases in intracellular Ca^2+^ that drive NLRP3 inflammasome activation and pyroptosis (25). Overall, identification of the causal role or roles of RRV-mediated cation transport and the specific cations involved remains unresolved.

Additional studies have proposed that impaired mitochondrial function drives the cytotoxicity of APOL1 RRVs (21–23, 26–28). However, identification of the cellular pathways that connect RRVs to mitochondrial dysfunction remain elusive. Other proposed mechanisms of RRV-mediated cytotoxicity (summarized in Supplemental Figure 1A; supplemental material available online with this article; https://doi.org/10.1172/JCI172262DS1) include aberrant activation of p38, JNK, and AMPK (21, 23), ER stress (29–31), inhibition of APOL3 function (32, 33), inhibition of global protein synthesis (21, 34), intracellular cholesterol accumulation (35), soluble urokinase plasminogen activator receptor–dependent (suPAR-dependent) integrin activation (36), and defective intracellular trafficking, including autophagy (11, 37, 38). There is a need for a new paradigm that weaves these seemingly disparate cellular events into a coherent pathomechanism of RRVs (Supplemental Figure 1B). The proximal, causal event would be expected to satisfy two criteria. First, inhibition of the proximal causal event would block all downstream cytotoxicity. Second, inhibition of the proximal causal event would provide durable cytoprotection against RRV-induced cell death.

Recently, Vertex Pharmaceuticals reported VX-147 (also known as inaxaplin) as a small-molecule blocker of APOL1 pore function (39). During the preparation of our current manuscript, the clinical efficacy of VX-147 in reducing proteinuria in APOL1-mediated kidney disease was reported (40). Our study provides the first detailed biochemical mechanism, to our knowledge, that explains how VX-147 protects kidney cells.

As recently reported, VX-147 is APOL1 specific (40). For research purposes, we synthesized VX-147 (Supplemental Figure 2) to investigate its capacity to block the APOL1 RRV–induced cascade of cytotoxic phenotypes and signaling events. We studied the commonly used HEK293 cell line with conditional APOL1 G1 expression as our discovery tool. Using a comprehensive suite of methodologies, we mapped a sequence of cytotoxic events, including previously unrecognized responses, following expression of APOL1 G1. We then validated key results in a human podocyte cell line and in APOL1 G1 transgenic mice.

Our findings establish APOL1 G1–mediated Na^+^/K^+^ transport as a necessary precursor to the IP3 receptor (IP3R) and ryanodine receptor–induced (RyR-induced) ER/Ca^2+^ signaling cascade that drives multiple downstream cytotoxic phenotypes, including mitochondrial dysfunction and reduced amino acid uptake, to inhibit global protein synthesis. This paradigm of APOL1 G1 cytotoxicity organizes new and known cellular phenotypes of APOL1 RRVs into a causality framework.

## Results

### APOL1 channel inhibitor prevents cell swelling and rescues cell viability.

**Figure 1:**
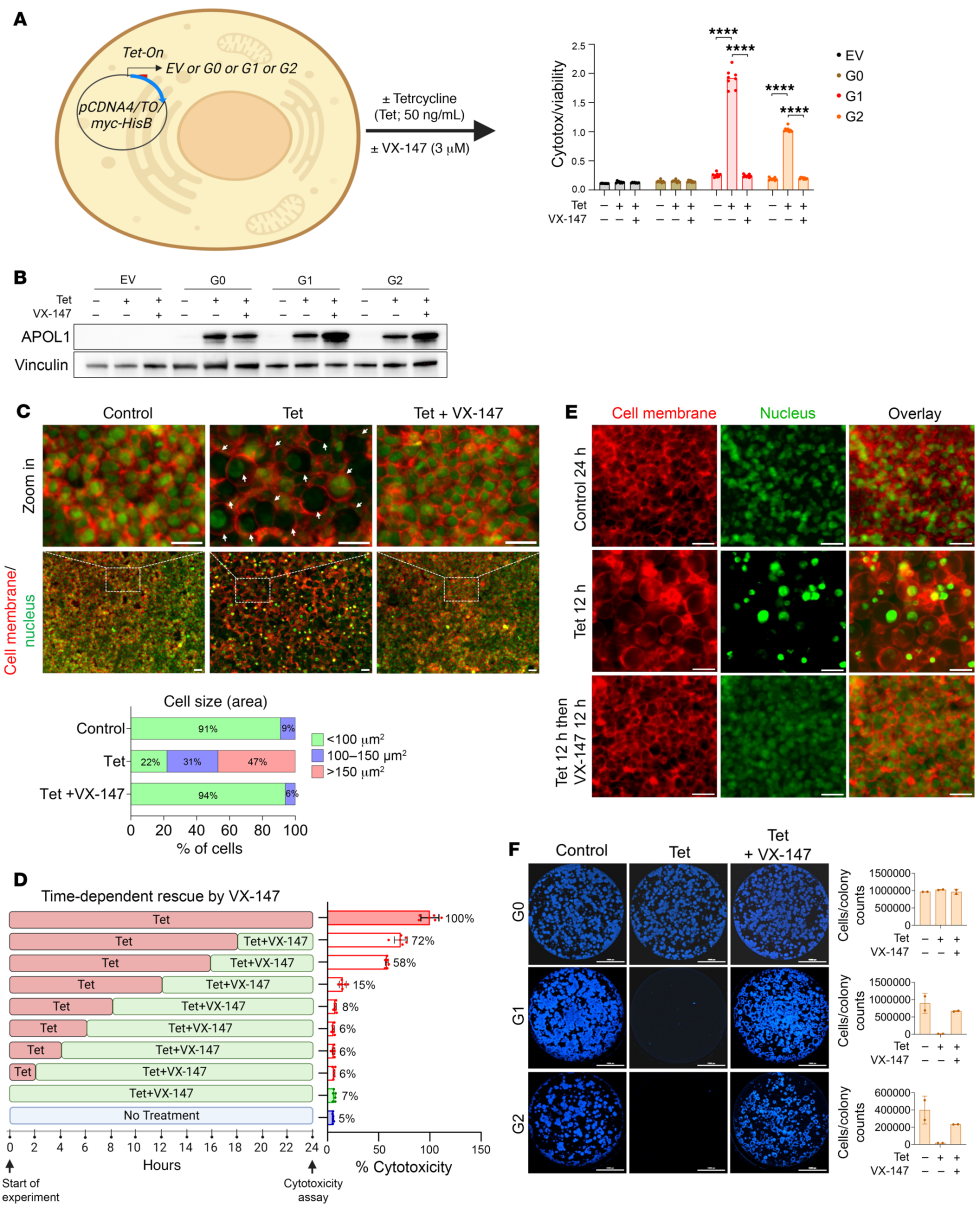
VX-147 protects T-REx-293 cells from APOL1 G1– and G2–induced cellular swelling and cell death. (**A**) Illustration of a T-REx-293 cell with Tet-inducible APOL1 G0, G1, or G2 expression construct. Tet-induced G1 or G2 but not G0 causes cytotoxicity at 24 hours that is completely prevented by VX-147 (*n* = 8). (**B**) Immunoblot of T-REx-293 cell lysates shows that Tet induces similar expression of G0, G1, and G2 proteins at 8 hours and VX-147 further increases G1 and G2 levels. (**C**) Live-cell fluorescence microscopy of T-REx-293 G1 cells shows that 8 hours of G1 expression causes cell swelling, pushing the PM (red) away from the nuclei (green), quantified in lower panel. Scale bars: 36.8 μm; zoom in scale bars: 311 μm. (**D**) Multitox assay shows that APOL1 G1 expression causes 100% cytotoxicity in T-REx-293 by 24 hours (topmost bar), an effect completely reversed by VX-147 within 12 hours and partially reversed at 16 hours following Tet induction (*n* = 6). (**E**) Live-cell fluorescence microscopy of T-REx-293 G1 cells shows that cell swelling caused by 12 hours of Tet-induced APOL1 G1 (top row) is reversed by a subsequent 12 hours of VX-147 treatment (middle row). Untreated T-REx-293 G1 cells served as controls. Scale bars: 311 μm. (**F**) Clonogenic survival assay (cell nuclei stained blue) performed after 10 to 12 days of continuous treatment shows that G0 expression does not affect the survival of T-REx-293 cells (top row), whereas G1 and G2 cause complete loss of cell survival that is rescued by VX-147. Scale bars: 10,000 μm (*n* = 3). Quantification of cells or colony counts (right panel). All data are represented as mean ± SD. ****P ≤ 0.0001, ordinary 1-way ANOVA with Tukey’s multiple-comparison test.

Previous observations support the hypothesis that APOL1 RRV–mediated cation transport across the PM drives cellular swelling and cell death (8, 9, 21, 23, 24). Here (9, 24), to test this hypothesis directly, we induced expression of APOL1 G0, G1, and G2 proteins in tetracycline-regulated (Tet-regulated) expression-293 (T-REx-293) cells in the absence or presence of APOL1 channel blocker VX-147. Consistent with our prior reports (9, 21), Tet-induced APOL1 G1 and G2 expression caused robust cytotoxicity, while APOL1 G0 and empty vector (EV) control were nontoxic, despite similar levels of expression of all the APOL1 proteins (Figure 1, A and B). VX-147 (3 μM) completely blocked cytotoxicity caused by G1 and G2 (Figure 1A). The choice of a 3 μM concentration of VX-147 was based on an initial exploratory dose-response curve (Supplemental Figure 3) and findings of its high selectivity at that dose when tested in many off-target assays (40). Cotreatment of G1-expressing T-REx-293 cells with Tet (to induce G1 expression) and VX-147 for 8 hours completely prevented cell swelling, making these cells indistinguishable from untreated controls (Figure 1C). Together, these results suggest that APOL1 RRV–mediated cation transport is upstream of cellular swelling and cell death in this model.

Next, we tested the durability of VX-147 effects. We induced APOL1 G1 expression in T-REx-293 cells for variable time points followed by the addition of VX-147 and measured cytotoxicity after 24 hours. VX-147 reversed cytotoxicity if added within 12 hours of APOL1 G1 expression, whereas when added at 16 hours, cytotoxicity was partially reversed (Figure 1D). VX-147 also rescued APOL1 G1–induced cellular swelling when added up to 12 hours following induced expression (Figure 1E). The ability to rescue APOL1 toxicity after protein production is consistent with a primary role for APOL1 ion channel function in cytotoxicity rather than an effect of mutant APOL1 variant protein overexpression per se.

We tested to determine whether the cytoprotection conferred by VX-147 is long-lasting. The multiplex cytotoxicity assay relies on the measurement of protease activity as a surrogate for cell viability and cellular injury, which may or may not result in cell death. We performed a clonogenic survival assay to measure the effect of APOL1 channel inhibitor on T-REx-293 cell survival after 10 to 12 days of constant APOL1 G1 expression in the absence or continued presence of VX-147. As shown in Figure 1F, expression of APOL1 G1 for 8 days in the absence of the inhibitor left no surviving HEK293 clones. In comparison, clonogenic survival of VX-147–treated, APOL1 G1–expressing cells was similar to that of uninduced controls at day 8. This shows that inhibition of APOL1 G1 channel function confers a durable protection against cytotoxicity of APOL1 G1.

### APOL1 channel inhibitor does not reduce APOL1 expression or its localization to the PM.

**Figure 2:**
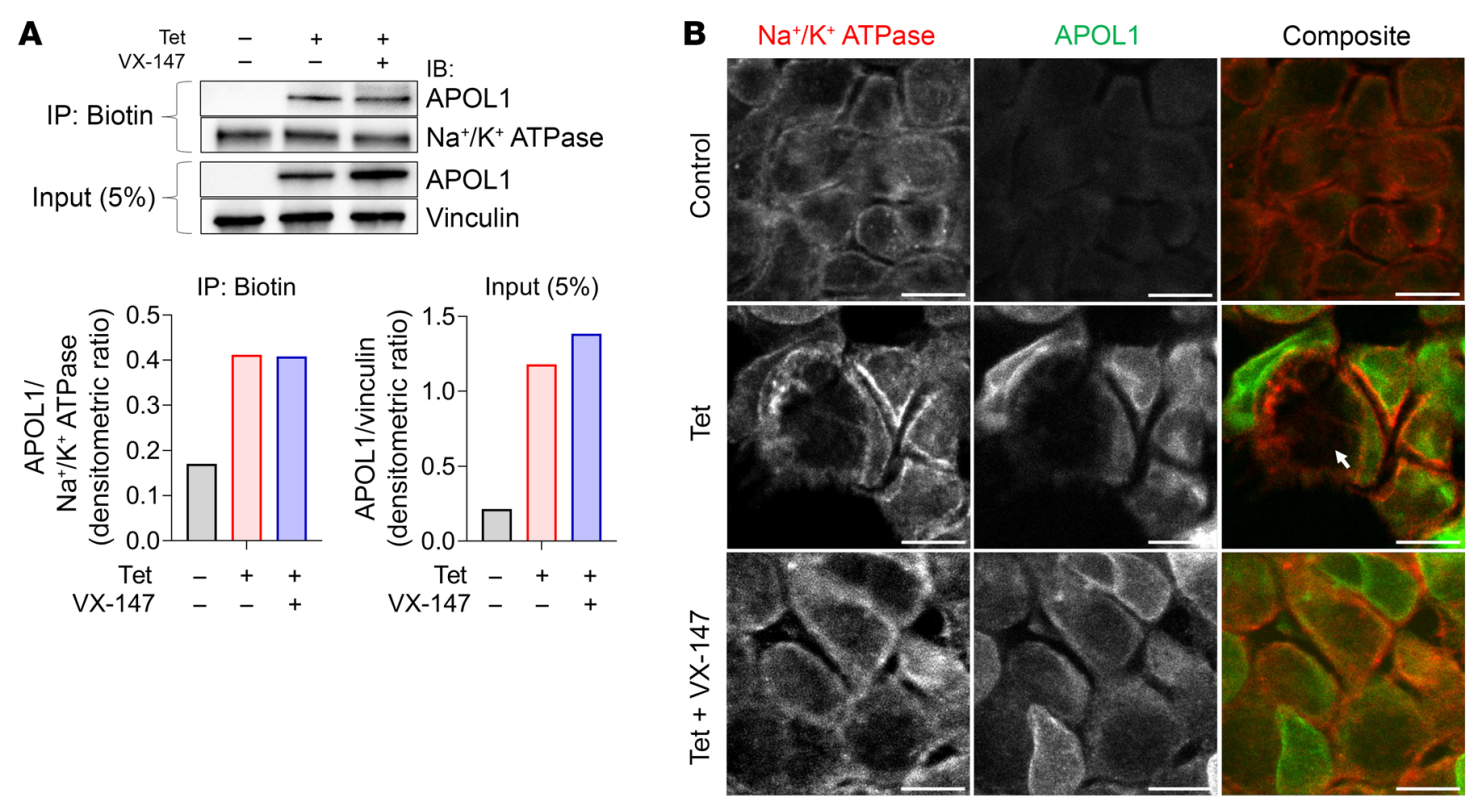
VX-147 does not reduce APOL1 localization to the PM. (**A**) Representative immunoblots of PM-localized, biotin-labeled APOL1 G1 and Na^+^/K^+^-ATPase proteins in T-REx-293 G1 cells after 8 hours of treatment, showing that VX-147 does not affect PM APOL1 G1 protein levels. APOL1 and vinculin levels in total cell lysate served as expression controls. Densitometric analysis data are represented in bar diagrams. Representative of 2 independent experiments. (**B**) Immunofluorescence staining showing PM and cytoplasmatic localization of APOL1 G1 (green) in T-REx-293 cells following 8 hours of treatment with Tet. Na^+^/K^+^-ATPase (red) serves as the PM reference protein. White arrow indicates area with cellular swelling. Scale bars: 11 μm.

Previous studies have shown that cytotoxicity of APOL1 RRVs is dose dependent and requires translocation of the APOL1 protein to the PM (21, 24). Therefore, we investigated whether VX-147 affects levels of expression or PM localization of APOL1 G1 protein. We found that (9, 21) total APOL1 G1 protein expression was increased by VX-147 (Figure 2A), whereas Tet-induced APOL1 mRNA was unaffected by VX-147 (Supplemental Figure 4). Moreover, localization of APOL1 G1 to the PM was not altered by VX-147, as demonstrated by immunoprecipitation and immunostaining (Figure 2, A and B). Consistent with a prior report (41), we found that only a small proportion (approximately 5%) of endogenous APOL1 G1 protein was localized to the PM (Figure 2A). Based on these results, we conclude that the cytoprotective effects of VX-147 are not due to a decrease in G1 protein expression or translocation to the PM.

### APOL1 G1 causes early sodium influx and potassium efflux, triggering a delayed increase in intracellular calcium.

APOL1 is a nonselective cation channel, with evidence suggesting it conducts Na^+^, K^+^, and possibly Ca^2+^ ions (17, 24). The precise impact of temporal changes in the intracellular concentrations of each of these ions on RRV-mediated cellular signaling and cell death remains unclear. Therefore, we conducted experiments based on the premise that the intracellular concentrations of cations that are directly transported via APOL1 channels would be measurable shortly following APOL1 induction and inhibited in the presence of VX-147.

**Figure 3:**
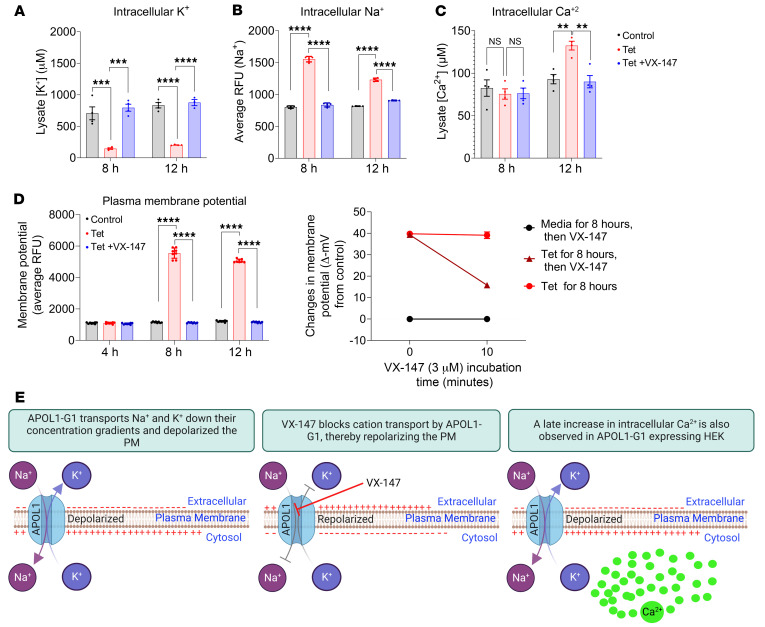
APOL1 G1 mediates rapid efflux of K^+^ and influx of Na^+^ associated with a delayed increase in intracellular Ca^2+^. (**A**) XRF spectroscopy (*n* = 4) and (**B**) real-time sodium indicator ING2 show G1-induced depletion of intracellular K^+^ and G1-induced import of Na^+^, respectively, in T-REx-293 as early as 8 hours after induction (*n* = 4). Both events are reversed by VX-147. Based on calibrated ING2 fluorescence (Supplemental Figure 5), 8 hours of G1 expression results in an approximate intracellular (Na^+^) of 40–50 mM. (**C**) XRF spectroscopy shows that increased intracellular Ca^2+^ is not detectable until 12 hours after G1 induction (*n* = 4). (**D**) Fluorescent membrane potential dye shows G1-induced depolarization of PM is detectable at 8 hours, coincident with G1-mediated Na^+^/K^+^ transport (*n* = 8). (**E**) G1-induced PM depolarization is reversible by 10 minutes of VX-147 treatment (*n* = 6). (**F**) Schematic summary of **A**–**D**. All data are represented as mean ± SD. **P ≤ 0.005; ***P ≤ 0.001; ****P ≤ 0.0001, ordinary 1-way ANOVA with Tukey’s multiple-comparison test.

Consistent with this prediction, 8 hours of APOL1 G1 expression causes a simultaneous reduction of intracellular K^+^ and an increase in intracellular Na^+^ that is prevented with VX-147 cotreatment (Figure 3, A and B). In agreement with a prior report (24), we also found that APOL1 G1 expression depolarized the PM at the same time point (Figure 3, D and E). Based on calibration of the membrane potential fluorescence response, we found that G1 caused a 40 mV depolarization of the PM in treated cells compared with control HEK cells (Figure 3, D and E, and Supplemental Figure 5, A–D). HEK cells cultured with VX-147 during the 8 to 12 hours of G1 expression were protected from G1-induced PM depolarization (Figure 3D). Moreover, 10 minutes of VX-147 treatment following 8 hours of uninterrupted G1 expression was sufficient to repolarize the PM (Figure 3E). Together, these results support our conclusion that APOL1 G1 directly mediates reciprocal exchange of Na^+^ and K^+^ across the PM down their respective concentration gradients, causing PM depolarization (Figure 3F). In contrast, the increase in total intracellular Ca^2+^ was not detectable until 12 hours following G1 induction (Figure 3C). The 4-hour delay in the change of intracellular Ca^2+^ relative to the change in intracellular Na^+^ and K^+^ concentrations suggests that the rise in intracellular Ca^2+^ may not be a direct effect of APOL1 G1 cation transport function. Nonetheless, the presence of VX-147 also abolished the rise in total Ca^2+^ (Figure 3C). The apparent delay in the increase of intracellular Ca^2+^ suggests that Ca^2+^ transport is likely a downstream consequence of G1-mediated transport of Na^+^ and K^+^.

### APOL1 G1 triggers the release of ER Ca^2+^ by activating Gαq-PLC-IP3R/RYR signaling.

Next, we investigated the underlying causes of increased intracellular Ca^2+^ levels following APOL1 G1 induction. Analysis of T-REx-293 cell global transcriptomic data predicts that APOL1 G1 expression activates Ca^2+^ release from the ER via IP3R and that VX-147 blocks this pathway (Supplemental Figure 6A and Supplemental Table 1). Further analysis also predicts that XSpC, an IP3R inhibitor, would mimic the effect of VX-147 by blocking calcium-induced signaling (Supplemental Figure 6B and Supplemental Table 2). To test the hypothesis that APOL1 G1 channel function triggers the depletion of ER Ca^2+^ and that VX-147 reverses this effect, we treated cells with thapsigargin. At basal state, ER Ca^2+^ content reflects the balance of Ca^2+^ pumped into the ER by sarco/ER Ca^2+^-ATPase (SERCA) and Ca^2+^ released from the ER via the calcium transporters IP3R and RyR. Thapsigargin inhibits the SERCA pump, thereby blocking transport of Ca^2+^ into the ER. The existing Ca^2+^ within ER leaks back into the cytosol and can be measured (42, 43).

**Figure 4:**
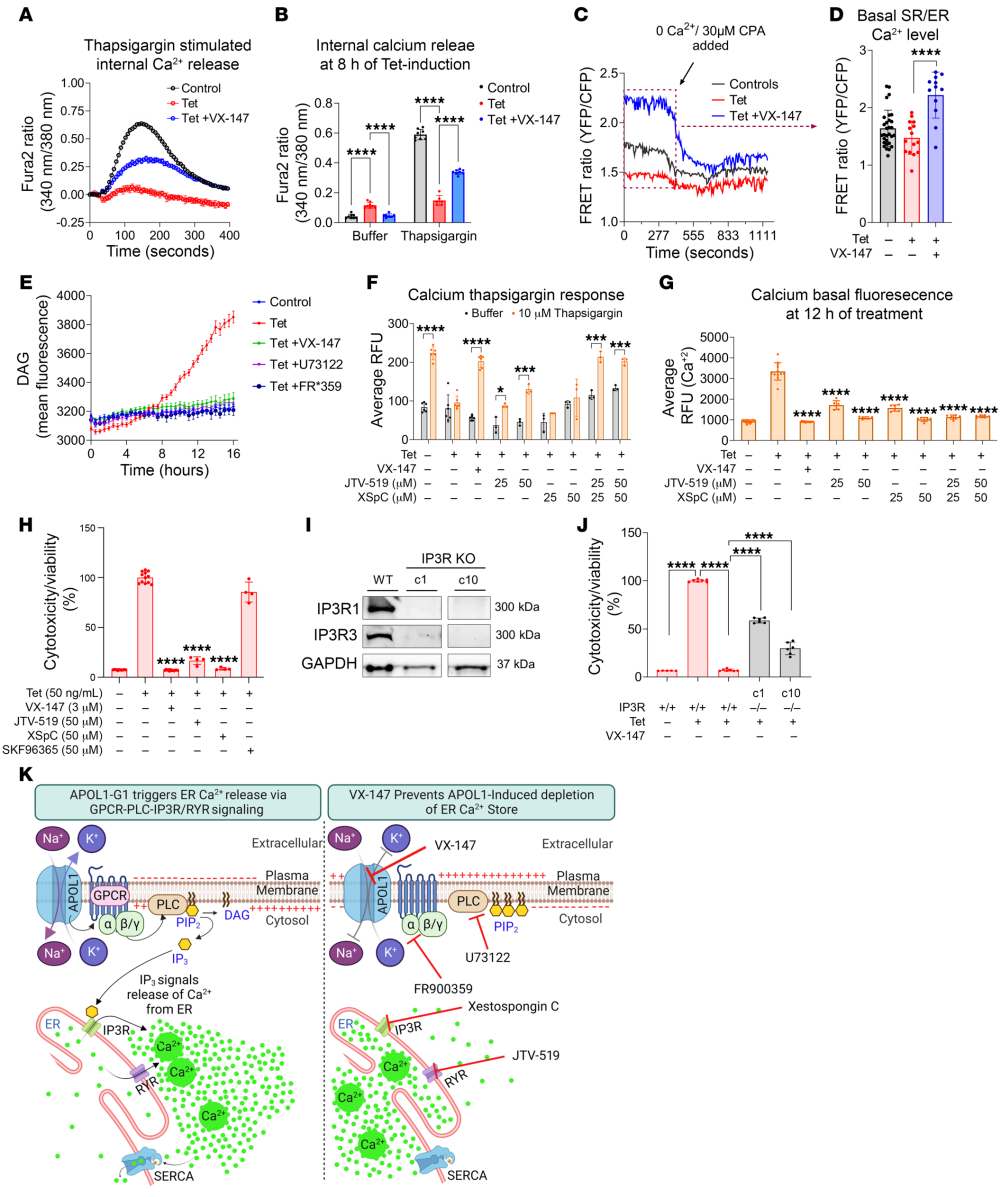
APOL1 G1 activates Gαq-PLC-IP3R/RYR signaling to liberate calcium from the ER into the cytosol of T-REx-293 cells. (**A**) Fura-2 Ca^2+^ sensor shows that 8 hours of APOL1 G1 expression attenuates thapsigargin-induced surge of cytosolic Ca^2+^, which is rescued by VX-147 cotreatment (*n* = 8). (**B**) Thapsigargin-induced Ca^2+^ release plotted as bar graphs (*n* = 8). (**C**) Direct measurement of ER Ca^2+^ with fluorescent sensor (D1ER) shows that 8 hours of APOL1 G1 expression depletes basal ER Ca^2+^, which is restored by VX-147 cotreatment (*n* = 8–12). Addition of CPA, which further depletes ER Ca^2+^, has marginal effect on ER Ca^2+^ of APOL1 G1–expressing cells. (**D**) Basal ER Ca^2+^ level before CPA addition shown as bar graph (*n* = 12–30). (**E**) Real-time live-cell fluorescence shows increased DAG biosynthesis after 8 hours of G1 induction. G1-induced DAG synthesis is blocked by VX-147, U73122 (PLC inhibitor), and FR*359 (Gαq inhibitor) (*n* = 8). (**F**) Fluorescent Ca^2+^ sensor shows that combined inhibition of IP3R with XSpC and of RYR with JTV-519 rescues thapsigargin-induced Ca^2+^ response in G1-expressing T-REx-293 cells (12 hours of treatment), similar to the effect of VX-147 (*n* = 3–6). (**G**) Measurement of live-cell basal cytosolic Ca^2+^ levels shows that VX-147, XSpC, and JTV-519 cotreatment prevents G1-induced increase in cytosolic Ca^2+^ (*n* = 6–12). (**H**) Multitox assay shows that JTV-519, XSpC, and VX-147, but not SKF96365, rescue APOL1 G1–induced cytotoxicity in T-REx-293 after 24 hours of treatment (*n* = 4–12). (**I**) Immunoblot shows successful CRISPR/Cas9 knockout of IP3R1 and IP3R3 in T-REx-293 G1 cells (C1 and C10 are 2 independent clones). (**J**) Multitox assay shows loss of IP3R-mediated APOL1 G1–induced cytotoxicity in T-REx-293 after 24 hours of treatment (*n* = 5–7). (**K**) Schematic summary of Figure 4, showing how each step of this signaling cascade is reversible. All data are represented as mean ± SD. *P ≤ 0.05; ***P ≤ 0.001; ****P ≤ 0.0001, ordinary 1-way ANOVA with Tukey’s multiple-comparison test.

We measured thapsigargin-induced cytosolic Ca^2+^ levels following 8 hours of APOL1 G1 expression in the absence and presence of VX-147 and found that 8 hours of G1 expression depletes ER Ca^2+^ stores, as indicated by the blunted thapsigargin-induced increase in cytosolic Ca^2+^ (Figure 4, A and B). In contrast, cotreatment with VX-147 during G1 expression preserved ER Ca^2+^ stores, as indicated by their robust thapsigargin response (Figure 4, A and B). To validate this result, we also measured ER Ca^2+^ directly with a genetically encoded Förster resonance energy transfer–based (FRET-based) indicator, D1ER (44). We confirmed that the basal ER Ca^2+^ levels were indeed lower in APOL1 G1–expressing cells compared with both untreated controls and APOL1 G1–expressing cells cotreated with VX-147 (Figure 4, C and D). Like thapsigargin, cyclopiazonic acid (CPA) also inhibits the SERCA pump (45) and caused a steep drop in the ER Ca^2+^ of control cells and VX-147–treated G1-expressing cells compared with APOL1 G1–expressing cells. These results provide direct evidence that APOL1 G1 expression depletes ER Ca^2+^ levels and that VX-147 reverses this effect. The blunted thapsigargin response in APOL1 G1–expressing cells is consistent with the prediction that APOL1 G1–induced IP3R signaling mediates the release of Ca^2+^ from ER stores.

To investigate our hypothesis that APOL1 G1 channel function triggers the release of IP3 to activate IP3R, we measured the activity of phospholipase C (PLC), the enzyme that hydrolyzes phosphatidylinositol-4,5-bisphosphate (PIP2) to IP3 and diacyl glycerol (DAG). Given the relative ease of direct measurement of DAG compared with IP3, we measured DAG in T-REx-293 with and without APOL1 G1 expression and in the absence and presence of VX-147. After 8 hours of G1 expression, DAG levels were increased. This increase was blocked by VX-147, PLC inhibitor (U73122), and Gαq inhibitor (FR*359) (Figure 4E). Increased DAG synthesis was corroborated by increased phosphorylation of PKC in G1-expressing T-REx-293 cells and its reduction in VX-147–treated G1-expressing cells (Supplemental Figure 6). This result supports the hypothesis that APOL1 G1 channel function activates Gαq/PLC/IP3 signaling.

To further confirm the ER as the source of elevated cytoplasmic Ca^2+^ in G1-expressing cells, we pharmacologically inhibited IP3R and RyR with xestospongin C (XSpC) and JTV-519, respectively, and then repeated the thapsigargin challenge. It is known that Ca^2+^ released from the ER via IP3R can induce RyR-mediated Ca^2+^ release (46). We discovered that the combined inhibition of IP3R and RyR in G1-expressing cells conserved ER Ca^2+^ stores, as indicated by the restored thapsigargin response (Figure 4F). It is notable that the ER Ca^2+^–preserving effect of combined inhibition of IP3R and RyR is similar to the effect of VX-147 (Figure 4F). Consistent with our hypothesis that the ER is the source of the rise in cytosolic Ca^2+^ in G1-expressing cells, we found that combined inhibition of IP3R and RYR prevented a rise in basal cytosolic Ca^2+^ similar to the effect of VX-147 (Figure 4G and Supplemental Figure 5E). In comparison, SKF96365, a store-operated Ca^2+^ entry (SOCE) inhibitor, did not reduce the G1-induced increase in cytosolic Ca^2+^ (data not shown), suggesting that the increase in intracellular Ca^2+^ is not due to activation of SOC channels.

### APOL1 G1–induced,IP3R/RYR-mediated ER Ca^2+^ release is cytotoxic.

Next, we investigated whether the inhibition of ER Ca^2+^ release affects the cytotoxicity caused by APOL1 G1. We discovered that both JTV519 and XSpC reduced APOL1G1-induced cytotoxicity, while SKF96365 did not (Figure 4H). The Ca^2+^ chelator BAPTA also reduced the cytotoxicity of APOL1 G1 (Supplemental Figure 6C), consistent with a prior report (25), thereby further buttressing the role of Ca^2+^ signaling in this cytotoxic cascade.

To further investigate the role of IP3R in mediating the release of Ca^2+^ from the ER, we used CRISPR/Cas9 to genetically knock out IP3Rs in HEK293 G1 cells. Global transcriptomic data from unedited HEK293 cells show that the 3 *IP3R* genes are expressed with *ITPR3*>*ITPR1*>*ITPR2* (Supplemental Figure 6E) and that their protein levels are not affected by expression of APOL1 G1 (Supplemental Figure 6F). We confirmed robust expression of IP3R1 and IP3R3 in unedited HEK cells and absence of detectable IP3R1 and IP3R3 in CRISPR-edited clones (Figure 4I and Supplemental Figure 6G). IP3R2 protein was not detected by immunoblot analysis (data not shown). The loss of IP3R significantly reduced APOL1 G1–induced cytotoxicity, although not quite to the levels seen with VX-147 treatment (Figure 4J). Together, these results are consistent with the conclusion that APOL1 G1–mediated Na^+^ influx/K^+^ efflux induces ER Ca^2+^ release via IP3R and RYR and that increased cytosolic Ca^2+^ contributes to cytotoxicity (Figure 4K).

### APOL1 G1–induced IP3R/RYR-mediated ER calcium release impairs mitochondrial ATP production.

**Figure 5:**
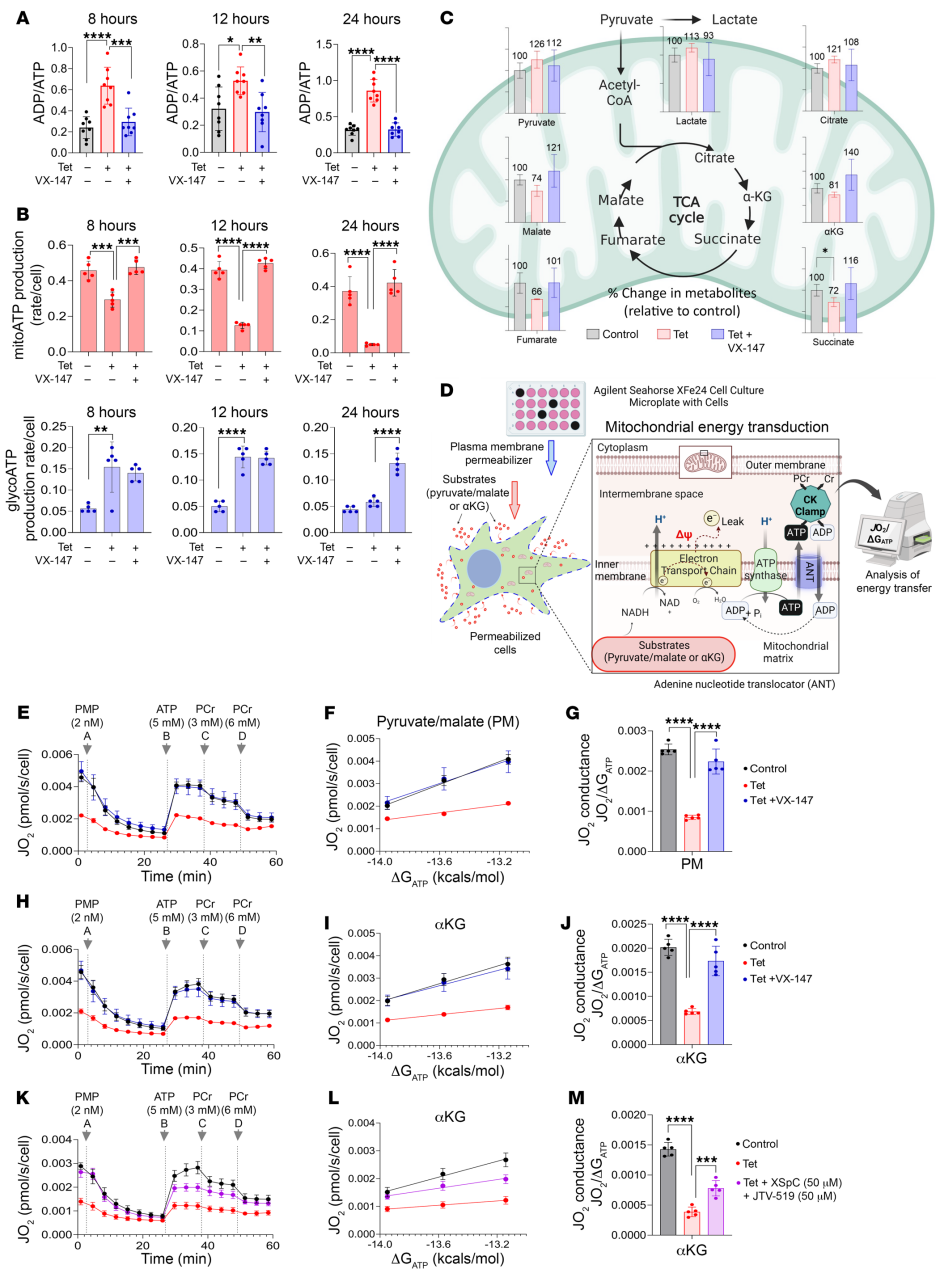
APOL1 G1–mediated cation transport reduces mitoATP production by impairing mitochondrial structure in T-REx-293 cells. (**A**) In T-REx-293 cells, induction of APOL1 G1 for 8, 12, or 24 hours increases the ADP/ATP ratio, which is normalized by VX-147 (*n* = 8). (**B**) Seahorse XFp real-time ATP rate assay shows that induction of APOL1 G1 for 8, 12, or 24 hours reduces mitoATP production, which is rescued by cotreatment with VX-147 (*n* = 5). Tet-induced glycoATP production is independent of APOL1 G1. (**C**) Targeted metabolomics measurement of TCA cycle metabolites in T-REx-293 G1 cells ± VX-147 for 8 hours (*n* = 3). (**D**) Schematic illustration of the assessment of mitochondrial respiratory conductance via the CK clamp in T-REx-293 G1 cells. Cells were treated for 12 hours with Tet ± VX-147. (**E**–**M**) Assessment of mitochondrial respiratory conductance via the creatine kinase clamp method in T-REx-293 G1 cells (*n* = 5). (**E**–**G**) Relationship between mitochondrial oxygen consumption (*J*O_2_) and ATP free energy (ΔG_ATP_) in permeabilized cells energized with either 5 mM pyruvate/2.5 mM malate, or 5 mM αKG. G1 reduces *J*O_2_, which is rescued by VX-147, but not by supplemental pyruvate/malate or αKG. (**K**–**M**) Cotreatment with XSpC and JTV-519 in cells with G1 expression improved *J*O_2_. Changes in *J*O_2_ in the presence of pyruvate/malate (**E**) or αKG (**H** and **K**). Analysis of the linear relationship between energy demand (ATP:ADP, ΔG_ATP_) and steady-state oxygen flux (*J*O_2_; **F**, **I**, and **L**) was used to determine respiratory conductance (*J*O_2_/ΔG_ATP_), whereby a higher slope indicates greater respiratory kinetics in response to changes in energy demand (**G**, **J**, and **M**). All data are represented as mean ± SD. *P ≤ 0.05; **P ≤ 0.005; ***P ≤ 0.001; ****P ≤ 0.0001, ordinary 1-way ANOVA with Tukey’s multiple-comparison test.

Impaired mitochondrial ATP (mitoATP) production is the most consistently reported cellular event in RRV-expressing cells (21–23, 26, 27, 47). Based on the ability of APOL1 inhibition to rescue toxicity, we hypothesized that APOL1 G1–mediated perturbation of cation fluxes triggers the reduction in mitoATP production. To test this hypothesis, we induced the expression of APOL1 G1 in T-REx-293 cells in the absence and presence of VX-147 for 8, 12, or 24 hours. As shown in Figure 5A, expression of APOL1 G1 increased the ADP/ATP ratio as early as the 8-hour time point, whereas VX-147 completely normalized the ADP/ATP ratio in APOL1 G1–expressing cells (Figure 5A). To further investigate the factors contributing to impaired ATP production, we measured oxidative and glycolytic ATP (glycoATP) resynthesis rates in whole cells. We found that APOL1 G1 reduced the rate of mitoATP production, consistent with prior reports (Figure 5B) (21–23, 26, 27). Importantly, VX-147 prevented the reduction of mitoATP in G1-expressing cells (Figure 5B). G1 expression increased glycoATP production, an effect not inhibited by VX-147 (Figure 5B), suggesting that Tet-induced glycolysis is independent of G1 cation transport function and consistent with reports that Tet increases glycolysis in eukaryotic cells, including HEK293 cells (48). Consistent with this interpretation, we observed a similar increase in glycolysis in Tet-treated EV and G0 HEK cells (Supplemental Figure 7). Total ATP production rate (mitoATP plus glycoATP) in G1-expressing cells was significantly lower than in control cells, but rescued in VX-147–treated cells. These results suggest that impaired mitoATP production is downstream of APOL1 G1–mediated cation transport. Note that APOL1 G0, which in this cellular model does not alter Na^+^/K^+^ transport function (9, 21), had no effect on mitoATP production (Supplemental Figure 7, A–C).

We hypothesized that G1-mediated cation transport reduces mitoATP production by 1 of 2 possible mechanisms: (a) depletion of TCA cycle metabolites or (b) altering mitochondrial structural integrity. To measure TCA cycle metabolites, we performed targeted metabolomic analysis of HEK cell lysates after 8 hours of G1 expression in the presence or absence of APOL1 channel inhibitors. We discovered that G1-expressing cells have reduced levels of α-ketoglutarate (αKg), succinate, fumarate, and malate (Figure 5C), confirming a prior report (26). Notably, coincubation of G1-expressing HEK cells with VX-147 restored the depleted TCA cycle metabolites (Figure 5C).

We next performed Seahorse-based measurement of oxygen consumption rates using permeabilized T-REx-293 cells, a method that affords a direct assessment of respiratory function in a cell-free system while retaining an intact mitochondrial reticulum (Figure 5D). We provided permeabilized cells with specific carbon substrates that feed the TCA cycle-αKg or pyruvate plus malate. Mitochondrial oxygen consumption rate (*J*O_2_) provides a measure of the efficiency and activity of the mitochondria. Expression of APOL1 G1 for 12 hours caused a significant reduction in *J*O_2_, which was not rescued by the supplemented TCA metabolites (Figure 5, E–J). However, inhibition of APOL1 G1 cation function by VX-147 during the 12 hours of APOL1 G1 expression prevented reduction in mitochondrial respiratory capacity (Figure 5, E–J). Given that APOL1 is localized to the PM but not mitochondria (41), our results support the conclusion that APOL1 G1 channel activity at the PM is the proximal cause of impaired mitoATP production.

It is known that increased cytoplasmic Ca^2+^ can trigger the opening of the mitochondrial permeability transition pore (mPTP), thereby causing reduced mitoATP production. Shah et al. previously reported that APOL1 RRV protein can translocate into the mitochondrial matrix, open mPTP, and inhibit ATP synthase (27). However, subsequent studies failed to confirm the presence of APOL1 protein in mitochondria (41). We hypothesize that G1-induced Ca^2+^ release from the ER opens mPTP, thereby contributing to the reduction of mitoATP production. Thus, we predict that inhibition of ER Ca^2+^ release would improve mitoATP production in G1-expressing cells. As shown in Figure 5, K–M, inhibition of IP3R and RYR-mediated ER Ca^2+^ release partially rescued mitochondrial oxygen consumption, supporting the conclusion that APOL1 G1–induced IP3R/RyR-mediated ER Ca^2+^ release contributes to mitochondrial impairment.

### APOL1 G1 inhibits global protein synthesis by reducing cellular amino acid import and by AMPK-mediated inhibition of mTORC1 and eIF2α.

**Figure 6:**
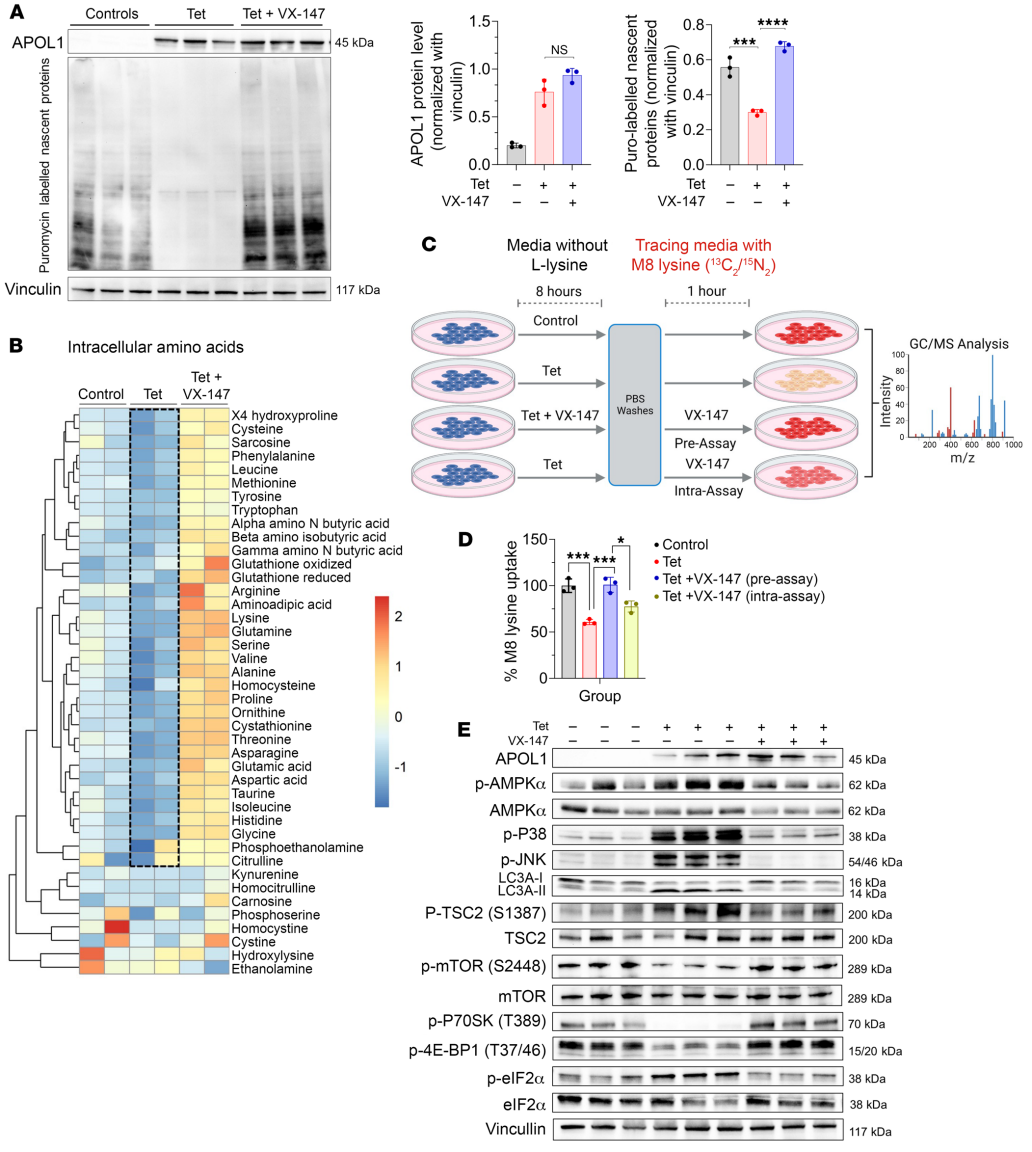
APOL1 G1–mediated cation transport reduces global protein synthesis by inhibiting amino acid import and activating AMPK. (**A**) Immunoblot of puromycin-labeled proteins shows that induction of APOL1 G1 in T-REx-293 G1 cells for 8 hours significantly reduces total protein synthesis, which is rescued by VX-147 (*n* = 3). Densitometric quantification of the data shown at right. (**B**) Targeted metabolomics of cell lysates shows that induction of APOL1 G1 in T-REx-293 G1 cells for 8 hours significantly reduces levels of several amino acids and related biogenic amines, which is rescued by VX-147 (*n* = 2). (**C**) Schematic illustration of experiments in which M8 lysine (U-^13^C, ^15^N-labeled) was used to trace the cellular uptake of lysine from the media into T-REx-293 cells. (**D**) Induction of APOL1 G1 in T-REx-293 G1 cells for 8 hours significantly reduces M8 lysine uptake, which is rescued by VX-147, and partially with VX-147 treatment only during the last 1 hour of the experiment (*n* = 3). (**E**) Immunoblot analyses showing the levels of AMPK, phospho-AMPKα (Thr172), AMPKα, phospho-tuberin/TSC2 (Ser1387), Tuberin/TSC2, phospho-P38 and p-JNK, and LC3A I-II in T-REx-293 G1 cells after 8 hours of treatment with Tet ± VX-147. Untreated cells (no treatment) served as controls. All data are represented as mean ± SD. *P ≤ 0.05; ***P ≤ 0.001; ****P ≤ 0.0001, ordinary 1-way ANOVA with Tukey’s multiple-comparison test.

We and other investigators previously reported that expression of APOL1 RRVs inhibits global protein synthesis (21, 34), but the underlying mechanism is not well understood. Okamoto et al. proposed that RRV RNA drives this effect by activating protein kinase R (PKR), which in turn phosphorylates and inhibits eukaryotic initiation factor-2α (eIF2α) (34). Here, we asked whether inhibition of global protein synthesis is a downstream effect of APOL1 G1 channel function. To investigate this question, we induced G1 expression in T-REx-293 cells for 8 hours in the absence or presence of VX-147, followed by the measurement of nascent global protein synthesis with a puromycin incorporation assay. Consistent with our previous report (21), global protein synthesis was significantly reduced in G1-expressing cells, whereas VX-147 completely restored global protein synthesis in these cells (Figure 6A). Because VX-147 does not affect levels of the APOL1 G1 mRNA transcript (Supplemental Figure 4), this result suggests that inhibition of global protein synthesis is a consequence of APOL1 G1 channel function rather than an effect on APOL1 RNA.

This result raises the possibility that G1-mediated cation transport causes a depletion of the intracellular amino acid pool and/or inhibits key regulators of protein translation. To test this hypothesis, we measured intracellular amino acid levels in HEK cells following 8 hours of G1 expression in the absence or presence of VX-147. Surprisingly, G1 expression caused clear decreases in levels of a broad array of amino acids, an effect completely prevented by VX-147 (Figure 6B). Given that import of several amino acids by amino acid/Na^+^ cotransporters is driven by the Na^+^ electrochemical gradient across the PM (49, 50), we hypothesized that disruption of the Na^+^ gradient by APOL1 G1 might attenuate Na^+^-facilitated amino acid transport. To test this hypothesis, we measured the uptake of labeled lysine in HEK cells after 8 hours of APOL1 G1 expression (Figure 6C). Expression of APOL1 G1 significantly reduced the uptake of labeled lysine (Figure 6D). However, when APOL1 G1–mediated transport of Na^+^ was blocked by VX-147 during the 8 hours of APOL1 G1 expression and the 1 hour lysine-uptake assay, lysine transport was completely restored (Figure 6D). Notably, inhibition of APOL1 G1–mediated Na^+^ transport by VX-147 for just 1 hour during the assay produced a significant, albeit incomplete restoration of labeled lysine uptake (Figure 6D).

^13^C-labeled lysine can be metabolized to citrate. Citrate labeling was similar across all experimental conditions, showing that the reduction in labeled cytosolic lysine was not caused by increased consumption of lysine, but was rather due to reduced import (Supplemental Figure 9H). Depletion of the intracellular pools of a host of other unlabeled amino acids in response to G1 expression and reversal of this effect by inhibition of Na^+^ import by VX-147 were further confirmed in these experiments (Supplemental Figure 9, I–Z). A reduction in the synthesis of creatine, creatinine, and phosphocreatine due to depletion of their amino acid precursors arginine, ornithine, and methionine illustrates the impact of this global amino acid depletion (Supplemental Figure 8, A and B). Additionally, we found that glutamic acid and its metabolite GABA are elevated in the media compared with cell lysates of G1-expressing cells, suggesting that G1-imported Na^+^ augments the export of these metabolites (Supplemental Figure 9F). Together, these results strongly suggest that G1-mediated import of Na^+^ causes the collapse of the Na^+^ electrochemical gradient, resulting in suppression of Na^+^-facilitated import of amino acids.

**Figure 7:**
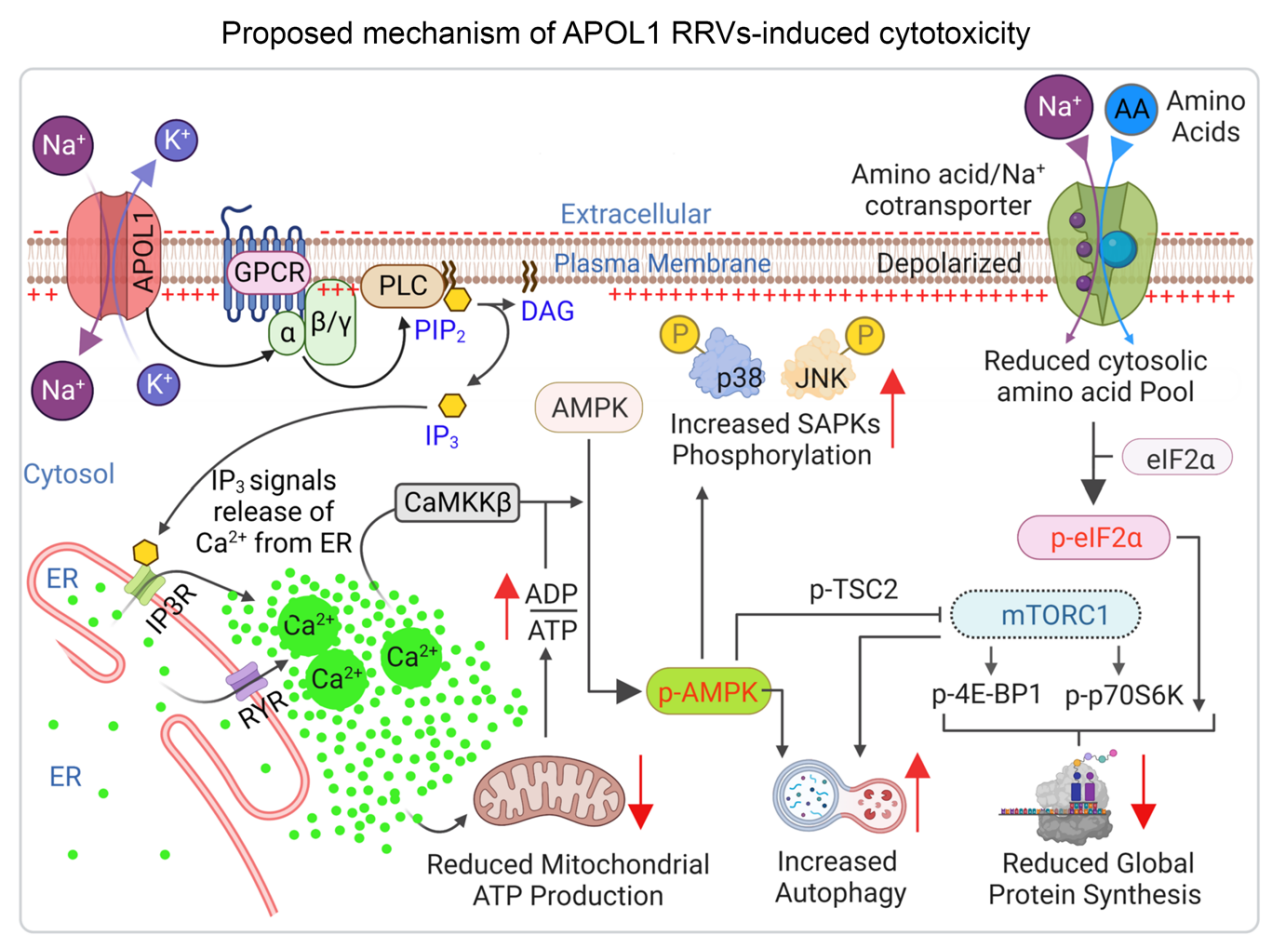
A unified model of RRV-induced cytotoxicity. APOL1 G1 forms cation pores in the PM where it transports Na^+^ and K^+^ down their concentration gradients, depolarizes the PM, and leads to activation of ER IP3R via GPCR/PLC/IP3 signaling. Ca^2+^ released via IP3R induces further Ca^2+^ release by RyR. Increased cytosolic Ca^2+^ impairs mitoATP production and increases the phosphorylation (activation) of AMPK by CaMKKβ. The activated AMPK promotes catabolic processes, such as autophagy, and inhibits anabolic processes, such as protein synthesis, via inhibition of mTORC1 and eIF2α. Additionally, APOL1 G1 collapses the Na^+^ gradient that drives amino acid import by amino acid/Na^+^ cotransporter. The resulting amino acid deficiency further inhibits protein synthesis.

Amino acid and energy deprivation can reduce global protein synthesis (51). We hypothesized that APOL1 G1–induced amino acid deprivation might increase eIF2α phosphorylation and thereby inhibit protein synthesis. Also, based on the analysis of transcriptomic data (Supplemental Figure 9, A and B), we hypothesized that APOL1 G1–induced energy deprivation would inhibit mTOR via AMPK/TSC2 signaling and also inhibit protein synthesis. Consistent with this hypothesis, we discovered that G1 expression increased phospho-eIF2α and that VX-147 blocked this increase (Figure 6E). We also found that G1 expression induces AMPK phosphorylation via Ca^2+^/calmodulin-dependent protein kinase II (CaMKII) and that VX-147 blocks this effect (Figure 6E and Supplemental Figure 9D). Consistent with established knowledge (52, 53), and as predicted by our transcriptomic analysis (Supplemental Figure 9, A and B), we found that in G1-expressing cells, activated AMPK phosphorylates TSC2, which in turn phosphorylates and inhibits mTORC1, thereby promoting inhibition of p70S6 kinase and dephosphorylation and activation of eukaryotic initiation factor 4E binding (4E-BP1) to inhibit protein translation initiation (Figure 6E). Importantly, the APOL1 channel inhibitor VX-147 prevented AMPK activation, mTOR inhibition, and eIF2α inhibition induced by APOL1 G1 expression (Figure 6E), thereby restoring protein synthesis (Figure 6A and Supplemental Figure 9E). Similarly, inhibition of AMPK by dorsomorphin also partially reversed eIF2α inhibition (phosphorylation) (Supplemental Figure 9D), partially restored protein synthesis (Supplemental Figure 9E), and reduced APOL1 G1–induced cytotoxicity (Supplemental Figure 9C). It is notable that VX-147 reversed eIF2α phosphorylation and restored protein synthesis without reversing PKR phosphorylation (Supplemental Figure 9G). This result suggests, contrary to a prior report (34), that increased PKR phosphorylation is not responsible for APOL1 G1–induced inhibition of global protein synthesis. Together, these results support our conclusion that APOL1 G1 reduces global translation by causing amino acid deprivation and inhibition of eIF2α and mTOR via Ca^2+^/AMPK/TSC2 signaling. Unsurprisingly, we also found increased phosphorylation of other AMPK substrates, including p38 and JNK, as well as increases in AMPK-regulated cellular events such as autophagy, as indicated by increased LC3II conversion in G1-expressing cells (Figure 6E and Supplemental Figure 9D). Based on all of these data derived from orthogonal approaches, we proposed a unified model of APOL1 G1–induced cytotoxicity in HEK cells (Figure 7).

### Validation of APOL1 G1 pathomechanism in human podocyte cell line.

**Figure 8:**
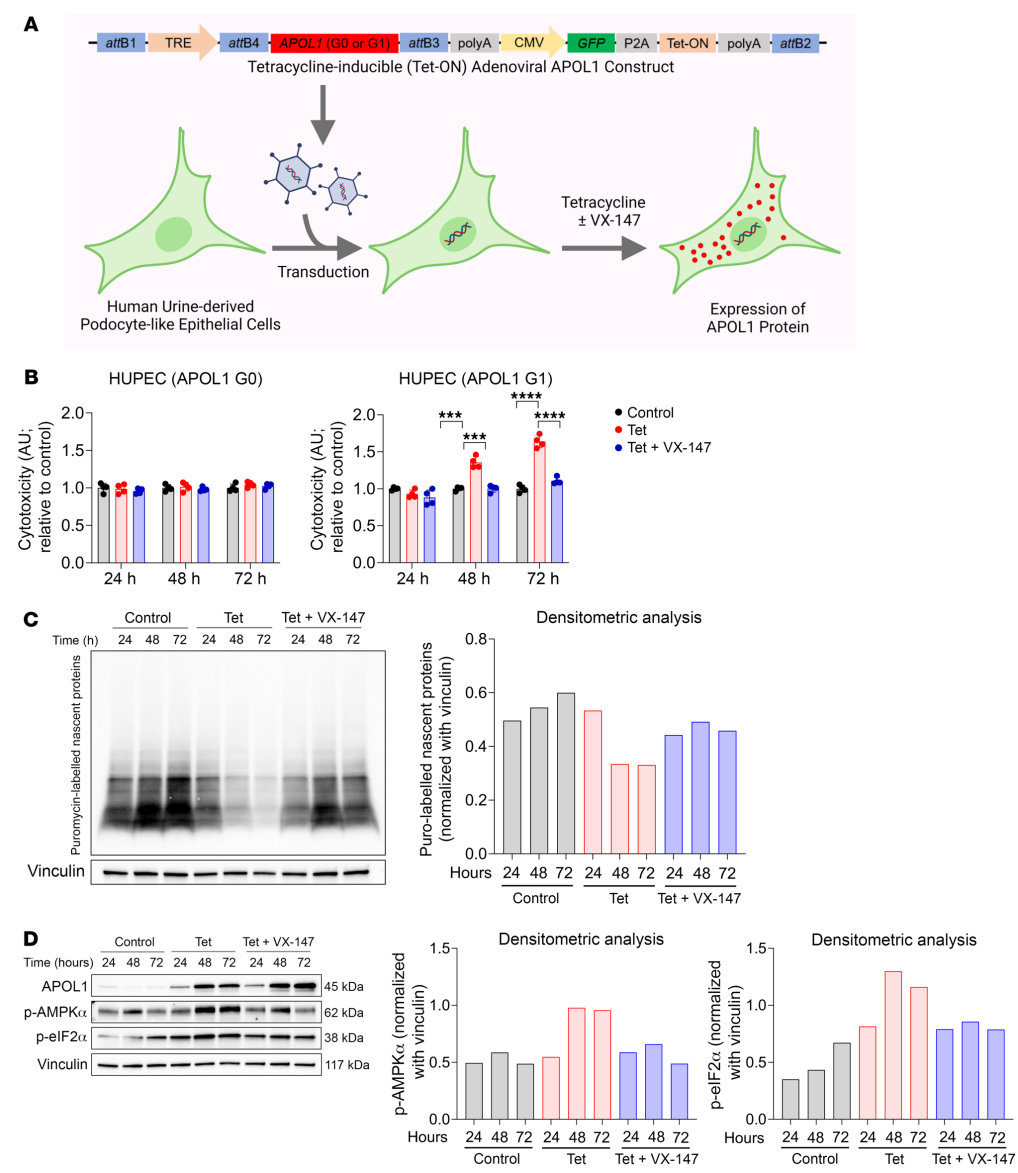
APOL1-induced podocytopathy is rescued by VX-147 in HUPECs. (**A**) Schematic summary of experimental design including the recombinant adenoviral vector designed for Tet-inducible expression of APOL1 G0 or G1 in HUPEC. (**B**) MultiTox-Fluor Cytotoxicity Assay shows that APOL1 G1, but not APOL1 G0, causes cytotoxicity that is detectable as early as 48 hours that is rescued by VX-147 coculture. Data normalized to untreated control cells set as 1 and represented as mean ± SD (*n* = 4). (**C**) Induction of APOL1 G1 in HUPECs reduces global protein synthesis as measured by puromycin labeling, which is rescued by VX-147, with densitometric quantification shown at right. (**D**) Immunoblot analyses of G1-expressing HUPECs showing increased levels of activated AMPK (p-AMPKα [Thr172]) and p-eIF2α. ***P ≤ 0.001; ****P ≤ 0.0001, ordinary 1-way ANOVA with Tukey’s multiple-comparison test.

Podocyte injury (podocytopathy) is the central feature of APOL1-mediated kidney disease. Therefore, we investigated whether the mechanism of APOL1 G1–induced cytotoxicity that we discovered in T-REx-293 cells is generalizable to podocytes and whether VX-147 will also reverse such APOL1 G1–induced podocytopathy. We transduced a conditionally immortalized human urine-derived podocyte-like epithelial cell (HUPEC) line (54, 55) with adenoviral vectors containing Tet-inducible *APOL1* G0 or G1 cDNA constructs (Figure 8A and Supplemental Figure 10). Consistent with results from T-REx-293 cells, we found that APOL1 G1 but not APOL1 G0 expression caused significant cytotoxicity in HUPECs and that VX-147 completely reversed this cytotoxicity (Figure 8B). Compared with T-REx-293 cells, in which cytotoxicity is detectable less than 24 hours following APOL1 G1 induction, APOL1 G1–induced cytotoxicity in HUPECs was detected at 48 hours, likely reflecting differences in intrinsic adaptive capacity of these 2 cell types to the cytotoxic effects of APOL1 G1. Consistent with results from T-REx-293 cells, we found that APOL1 G1 inhibited global protein synthesis in HUPECs (Figure 8C) and increased calcium-mediated signaling, as indicated by increased AMPK phosphorylation (Figure 8D), effects completely reversed by VX-147 (Figure 8, C and D). These results affirm that the pathomechanism of APOL1 G1 cytotoxicity in T-REx-293 cells also applies to APOL1 G1–induced podocytopathy.

### Validation of APOL1 G1 pathomechanism in primary podocytes of APOL1 G1 transgenic mice.

**Figure 9:**
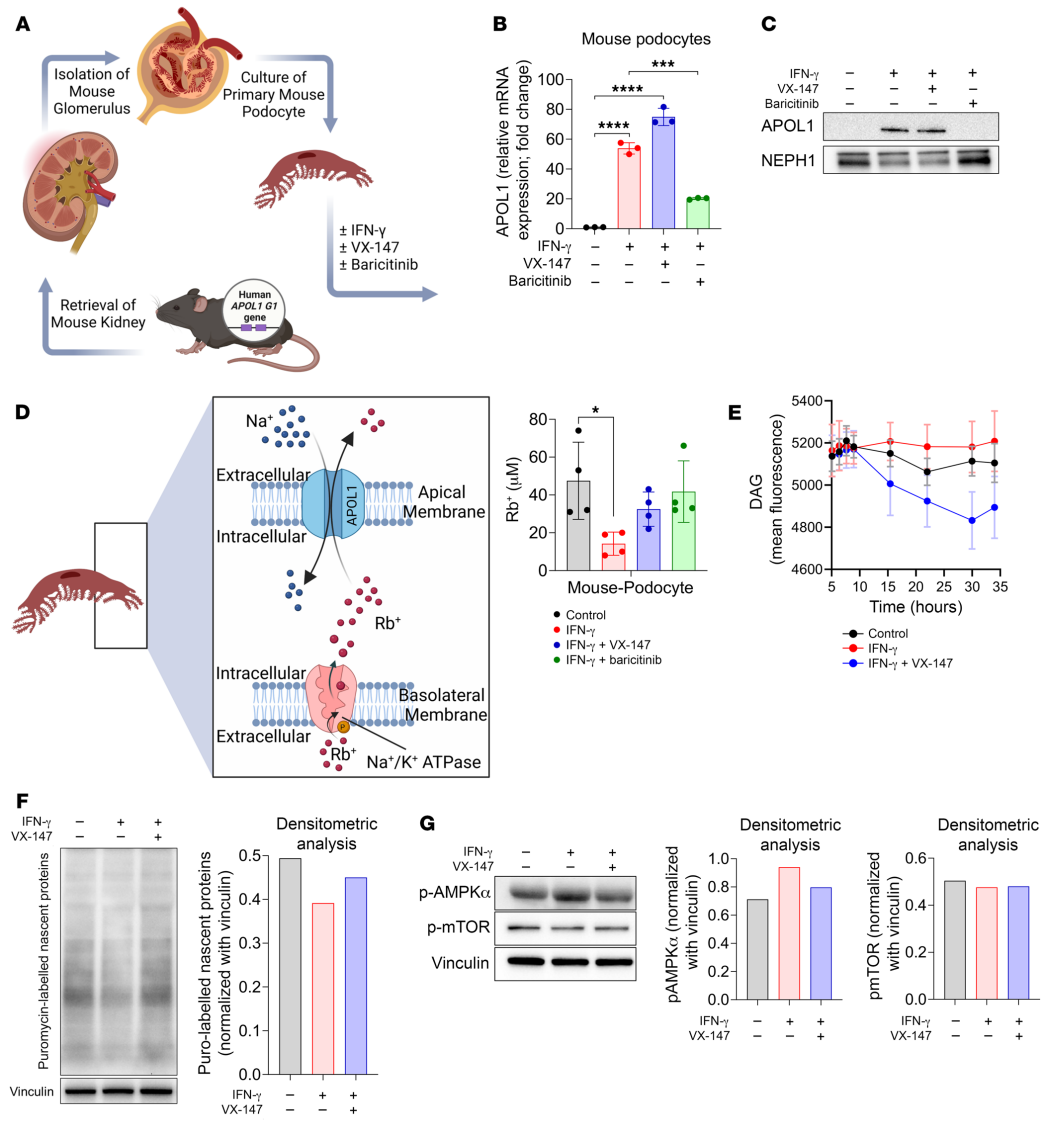
Physiologic expression of APOL1 G1 in primary mouse podocytes. (**A**) Schematics of primary podocyte isolation from APOL1 G1 transgenic mice followed by treatment with and without IFN-γ (10 ng/mL) ± VX-147 (3 μM) and ± Jak inhibitor baricitinib (10 μM) for 24 hours or 48 hours (*n* = 3). (**B**) IFN-γ increases APOL1 G1 mRNA expression (quantitative PCR [qPCR]) and (**C**) APOL1 G1 protein (immunoblot), effects blocked by baricitinib but not VX-147. GAPDH is used as internal control for qPCR fold-change analysis. Podocyte marker NEPH1 used as loading control for Western blot. (**D**) XRF spectroscopy of primary podocytes treated or not with IFN-γ (10 ng/mL) ± VX-147 (3 μM) and ± baricitinib (10 μM) for 45 hours shows that both VX-147 and baricitinib prevented APOL1 G1–mediated Rb^+^ efflux and preserved intracellular Rb^+^ (*n* = 4). (**E**) VX-147 reduces DAG synthesis in APOL1 G1–expressing podocytes (*n* = 7). (**F**) Induction of APOL1 G1 reduces global protein synthesis in mouse podocytes measured by puromycin incorporation, which is rescued by VX-147. (**G**) Immunoblot of p-AMPK, p-mTOR, and vinculin, performed 28 hours after APOL1 G1 activation. All data are represented as mean ± SD. *P ≤ 0.05; ***P ≤ 0.001; ****P ≤ 0.0001, ordinary 1-way ANOVA with Tukey’s multiple-comparison test.

To test the pathophysiologic relevance of APOL1 G1–mediated monovalent cation transport as the proximal driver of APOL1-mediated kidney disease in the in vivo setting, we generated APOL1 G1 transgenic mice. Unlike T-REx-293 and HUPEC models, which rely on artificial, Tet-induced overexpression of APOL1 G1, expression of human APOL1 G1 in this mouse model is under the control of natural human APOL1 regulatory elements. Exposure of these transgenic mice to systemic IFN-γ induces expression of human APOL1 G1 in the podocytes and other tissues via Jak/STAT-mediated signaling. We isolated primary podocytes from untreated APOL1 G1 transgenic mice and cultured them in vitro in the absence or presence of IFN-γ, with and without VX-147 and with and without baricitinib, an inhibitor of Jak1/2 kinases (Figure 9A). As expected, IFN-γ–induced APOL1 G1 expression was reduced by baricitinib, but not by VX-147 (Figure 9, B and C). Indeed, APOL1 mRNA increased in VX-147–treated mouse podocytes (Figure 9, B and C). In contrast to T-REx-293 and HUPECs, in which VX-147 increased APOL1 protein levels, this effect was absent in primary mouse podocytes (Figure 9C). Using rubidium (Rb^+^) as a tracer for potassium, we discovered that IFN-γ–induced APOL1 G1 increased Rb^+^ efflux and reduced intracellular Rb^+^ levels (Figure 9D), effects reversed by baricitinib or VX-147 treatment (Figure 9D). Furthermore, VX-147 reduced DAG biosynthesis in APOL1 G1–expressing podocytes (Figure 9E), and APOL1 G1 reduced global protein synthesis in the primary mouse podocyte, which was rescued by cotreatment with VX-147 (Figure 9F). Finally, APOL1 G1–expressing primary mouse podocytes had increased levels of p-AMPK, consistent with an increased energy demand, and VX-147 also reversed this phenotype (Figure 9G). Together, results obtained in T-REx-293 cells, HUPECs, and primary mouse podocytes were highly concordant, strongly supporting monovalent cation transport as the proximal mechanism underlying APOL1 G1–induced cellular injury.

### APOL1 G1 cation-channel function and podocytopathy pathogenesis in mice.

**Figure 10:**
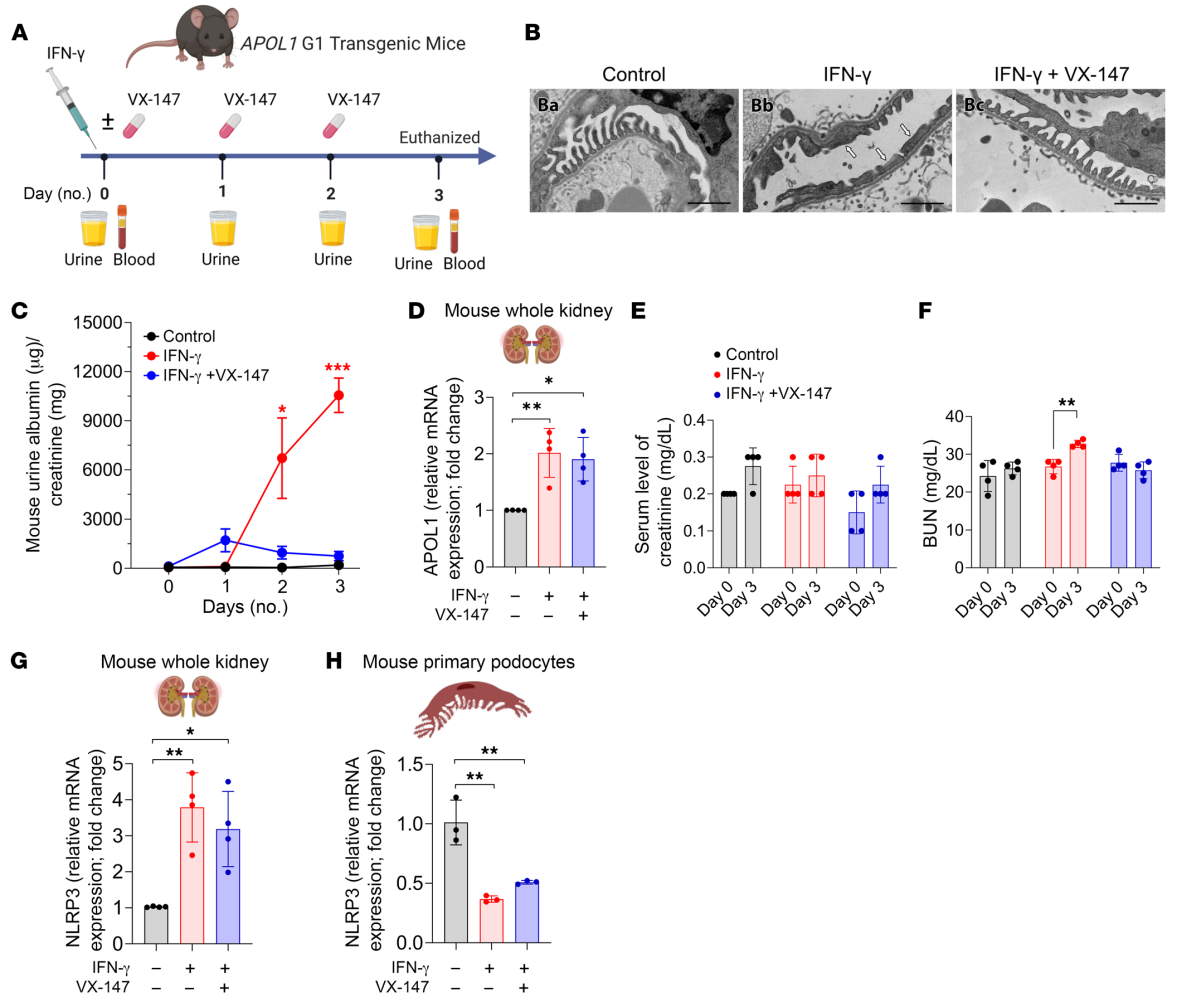
VX-147 rescues G1-induced podocyte injury and proteinuria in vivo. (**A**) Eight-week-old APOL1 G1 transgenic mice received 1 of 3 treatments: PBS injection (control), IFN-γ injection (on day 0), or IFN-γ injection on day 0 and VX-147 on days 0, 1, and 2 (*n* = 4 mice/treatment). (**B**) Representative electron micrographs from each of the 3 treatment groups. IFN-γ–treated APOL1 G1 mice developed focal podocyte foot process effacement (white arrows), microvillar transformation, and cytoplasmic shedding (Supplemental Figure 11). VX-147 rescued all of these histopathologic phenotypes. (**C**) IFN-γ–treated APOL1 G1 mice developed robust proteinuria by day 2, which was significantly attenuated by VX-147. (**D**) Measurement of APOL1 G1 expression in whole kidney lysate by qPCR shows that IFN-γ increased APOL1 expression and that VX-147 does not alter the expression level. (**E**) Serum creatine is unchanged, but (**F**) BUN is increased by IFN-γ treatment, an effect rescued by VX-147. (**G**) Measurement of NLRP3 by qPCR in whole kidney lysate shows increased NLRP3 mRNA transcript in IFN-γ–treated APOL1 G1 mice independent of VX-147. (**H**) IFN-γ–induced APOL1 G1 does not increase NLRP3 in primary podocytes. GAPDH was used as internal control for qPCR fold-change analysis (*n* = 3). All data are represented as mean ± SD. *P ≤ 0.05; **P ≤ 0.005, ordinary 1-way ANOVA with Tukey’s multiple-comparison test (**C**, **D**, **G** and **H**) and 2-tailed *t* test (**E** and **F**).

To investigate the impact of this mechanism in vivo, we injected APOL1 G1 transgenic mice with IFN-γ in the presence and absence of VX-147 coadministration. Within 48 hours, IFN-γ–treated mice developed significant proteinuria, which was almost completely attenuated with cotreatment with VX-147 (Figure 10, A and C). Podocyte injury causes proteinuria and can be detected at ultrastructural levels by electron microscopy. By day 3 of treatment, IFN-γ–treated APOL1 G1 mice developed classic features of podocyte injury, including focal podocyte foot process effacement, microvillar transformation, and cytoplasmic shedding (Figure 10B and Supplemental Figure 12). Importantly, these injury phenotypes were prevented by VX-147 treatment. IFN-γ–treated APOL1 G1 mice also developed elevated blood urea nitrogen, an effect reversed by VX-147 (Figure 10, E and F). Analysis of total mRNA from whole kidney, an aggregate of all kidney cells including podocytes, corroborates our results from primary mouse podocyte (Figure 9, A and B) by showing that IFN-γ induces APOL1 expression and that VX-147 does not reduce this expression (Figure 10D). It has been proposed that APOL1 induces podocytopathy and kidney injury by inducing NLRP3-mediated pyroptosis (25). We measured NLRP3 mRNA expression in whole kidney and in primary podocytes isolated from APOL1 G1 transgenic mice and found that, while IFN-γ increases NLRP3 expression in whole kidney, this increase was not reversed by VX-147 (Figure 10G). There was also no detectable increase of NLRP3 expression in IFN-γ–treated G1-expressing podocytes (Figure 10H), in accord with a recent report that podocytes do not express NLRP3 (56). These results are consistent with the conclusion that APOL1 G1–induced podocytopathy that we report in our mouse model occurred independently of NLRP3. Together, these results support our overarching conclusion that inhibition of APOL1 G1 monovalent-cation function is sufficient to prevent histological and clinical manifestations of APOL1-mediated kidney disease.

## Discussion

In this study, based on 3 experimental models — T-REx-293 cells, HUPECs, and APOL1 G1 transgenic mice — and a comprehensive, orthogonal experimental strategy, we have developed a paradigm of APOL1 G1–induced cytotoxicity. This unified model holds that APOL1 G1–mediated (and G2 mediated) transport of Na^+^ and K^+^ across the PM is the proximal, causal driver of downstream signaling that terminates in cytotoxicity in general and podocytopathy in particular, a major cause of APOL1-mediated kidney disease. Inhibition of this initial monovalent cation transport by VX-147 abolishes all measures of APOL1 G1–induced cellular injury in T-REx-293 and HUPEC cell line models as well as podocyte injury and proteinuria in APOL1 G1 transgenic mice (57, 58).

We also uncovered 2 new cellular functions of APOL1 G1. First, we demonstrate that APOL1 G1–mediated transport of Na^+^/K^+^ across the PM (and subsequent PM depolarization) triggers the release of Ca^2+^ from the ER via Gαq-PLC-IP3R/RYR signaling. Our observed 40 mV depolarization of T-REx-293, which typically has a resting PM potential of –20 mV, implies that APOL1 G1 is conducting more Na^+^ than K^+^. The basis of this unequal stoichiometry is unknown and warrants future studies. Second, we show that APOL1 G1 inhibits cellular amino acid import, causing intracellular amino acid deficiency, likely due to collapse of the PM Na^+^ gradient. It is also plausible that increased intracellular Na^+^ concentration may cause reduced Na^+^-Ca^2+^ exchange and further contribute to the rise in cytosolic Ca^2+^ in APOL1 G1–expressing cells in addition to the increased release of Ca^2+^ from the ER Ca^2+^ stores. We recognize that APOL1 G1–induced Ca^2+^ release from the ER could very well have broader pathologic roles than discussed here. Also, it is unclear at present whether the effect of APOL1 G1 on Ca^2+^ release is cell-type specific. Based on a prior report that the presence of Ca^2+^ increases APOL1-mediated K^+^ transport (59), it is plausible that the increased cytosolic Ca^2+^, mediated by Gαq-PLC-IP3R/RYR signaling, acts as positive feedback that augments APOL1 G1–cation transport. Further investigation of these issues will expand our knowledge of APOL1 function in physiologic and pathologic states.

Our results validate and extend conclusions of some, but not all, previous studies. First, we previously reported that APOL1 RRV–induced K^+^ efflux is an early molecular event that reversibly triggers activation of stress-activated protein kinases (SAPKs) and cytotoxicity (9, 21). While other investigators confirmed K^+^ efflux (17, 47) and SAPK activation (23) in APOL1 RRV–expressing cells, there was no general agreement that APOL1-mediated cation transport is the causal driver of cytotoxicity (14, 15, 60, 61). Moreover, the mechanisms that connect APOL1 RRV–mediated cation transport to SAPK activation were unknown. The present study provides the missing link by showing that APOL1 G1–mediated Na^+^/K^+^ transport causes ER Ca^2+^ release, which in turn activates AMPK to phosphorylate SAPKs and other substrates and to increase autophagosome formation.

Second, while we (21) and others (22, 23, 26, 27) have previously reported that expression of APOL1 RRV in various cell types impairs mitoATP production, there is no consensus on the causal mechanism. Additionally, there is debate as to whether aberrant cation transport by the APOL1 RRVs causes mitochondrial dysfunction or whether mitochondrial dysfunction causes impaired cation transport. Some investigators have proposed that the energy deficit caused by impaired mitoATP production causes unregulated influx of Na^+^ and efflux of K^+^ (9, 21, 24). The current study clearly demonstrates that APOL1-mediated Na^+^/K^+^ transport triggers ER Ca^2+^ release, which in turns impairs mitoATP production, likely via the opening of the mPTP. Because inhibition of ER Ca^2+^ release rescued approximately 50% of mitochondrial function, a Ca^2+^-independent mechanism may also mediate the effect of APOL1 on mitochondrial function.

Third, we and others (21, 34) reported that RRVs caused global reduction of protein synthesis (34). This effect has been attributed to eIF2α-mediated inhibition of protein synthesis via APOL1 RNA activation of PKR, which phosphorylates and inhibits eIF2α (34). Our data suggest instead that the inhibition of global protein synthesis is mediated by APOL1 G1 protein and not APOL1 mRNA. We demonstrate that APOL1 G1 cation transport inhibits global protein synthesis by Ca^2+^-induced activation of AMPK, which in turn inhibits mTORC1 and eIF2α, and by reducing amino acid import to deprive the protein-translation machinery of its essential substrates. Amino acid deficiency is known to increase eIF2α phosphorylation, thereby inhibiting its function. Our finding that VX-147 restores protein synthesis without preventing PKR phosphorylation strongly suggests that PKR phosphorylation is not essential for RRV inhibition of global protein synthesis. Amino acid deficiency is known to activate GCN2 — an eIF2α kinase (62). We detected a modest activation (phosphorylation) of GCN2 but not of PERK, suggesting that GNC2 contributes to the phosphorylation of eIF2α in APOL1 G1–expressing cells (Supplemental Figure 9G).

The current study is not the first to report that expression of APOL1 RRVs leads to increases in cytoplasmic Ca^2+^, but it is the first, to our knowledge, to demonstrate that the ER Ca^2+^ store is the source of the increased cytoplasmic Ca^2+^. Previously, Giovinazzo et al. reported that recombinant APOL1 is permeable to Ca^2+^ in a planar lipid bilayer and that APOL1 RRVs (but not G0) activate cytoplasmic Ca^2+^ influx, beginning approximately 12 to 18 hours after APOL1 induction. Because the Ca^2+^ influx seemed to require the trafficking of RRVs from the ER to the PM, they concluded that the source of Ca^2+^ is extracellular (24). While the current study confirms the reported timing of the increased cytoplasmic Ca^2+^, we find that ER Ca^2+^ store release is the principal source of this increase (25). Our conclusion is based on the demonstration that (a) the depletion of ER Ca^2+^, which is detectable at 8 hours after activation of APOL1 G1, precedes the rise of cytosolic Ca^2+^, which is detectable at 12 hours; (b) ER Ca^2+^ release is reversibly mediated by IP3R and RyR; (c) IP3R activation is a consequence of APOL1-induced PLC activation; and (d) inhibition of APOL1 G1 channel function, or Gαq-PLC-IP3R/RYR signaling, preserves ER Ca^2+^ and prevents the rise in cytosolic Ca^2+^. The mechanistic link between APOL1 G1 channel function activation and Gαq-PLC activity is still incomplete. A prior report indicates that depolarization of the PM brings PIP2 and PLC closer together, thereby potentiating the formation of IP3 (63). However, optimal activation of PLC requires a G protein. Identification of the GPCR that mediates the effect of APOL1 G1 channel function merits future investigation.

Our use of APOL1 G1 transgenic mice in which the physiologic expression of APOL1 G1 is driven by the human APOL1 promoter addresses the potential concern of artifactual effects of overexpressed APOL1 in cultured cells (25). Our mouse model not only corroborates the results from T-REx-293 and HUPEC models, showing that APOL1 channel function is a causal mechanism of toxicity, but also demonstrates the relevance of this mechanism in an in vivo setting.

This study has limitations. While we evaluated the impact of APOL1 pore blockage on several previously proposed mechanisms of RRV-mediated cytotoxicity, we were unable to investigate them all. For instance, we did not explore the impact of VX-147 on APOL1-associated impairment of cholesterol efflux (35), reduced association of RRVs with lipid droplets (31), inhibition of APOL3 (33), and suPAR-dependent integrin activation (36). However, the fact that a pharmacologic APOL1 channel inhibitor completely restored cell viability and mitigated a host of other known APOL1 G1–induced responses strongly suggests that these mechanisms are also downstream of APOL1 cation-transport function. Because our study is based on experimental models, additional studies in human kidney tissue are required to validate the relevance of our results to human APOL1-mediated kidney disease. However, the clinical relevance of our results is strongly supported by recent reports that naturally occurring genetic inhibition of APOL1 pore function prevents APOL1-mediated kidney disease in humans (57, 58).

In conclusion, results of this study advance the current understanding of APOL1 biology as it relates to the cytotoxic effects of APOL1 RRV expression in podocytes and possibly other cell types. From an evolutionary point of view, our results describe a molecular boomerang that strikes its thrower. *APOL1 G1* (and *G2*) variants emerged as mediators of human innate immunity against *Trypanosoma brucei rhodesiense* (1, 64). It is the potent cation pore function of these *APOL1* variants that lyses *Trypanosoma* parasites (17, 19). Ironically, the current study demonstrates that the same cation pore function of APOL1 G1 also drives podocyte injury and kidney disease. Our paradigm offers a unified mechanism of APOL1-mediated cytotoxicity that explains previously reported cytotoxic phenotypes of RRVs. It also provides a rationale that supports the clinical development of APOL1 modulators such as inaxaplin (ClinicalTrials.gov NCT04340362) and inhibitors of APOL1 synthesis such as Janus kinase-STAT inhibitors (ClinicalTrials.gov NCT05237388) as promising therapeutic strategies for APOL1-mediated kidney disease. Finally, we believe that the proposed paradigm will inform future investigations of APOL1 biology and pathobiology.

## Methods

Complete details regarding experimental procedures are provided in the Supplemental Methods.

### Generation of T-REx-293 cell line and HUPECs with inducible APOL1.

T-REx-293 APOL1 G0 (KIK haplotype background, i.e., K150, I228, K255), APOL1 G1 (EIK haplotype background; i.e., E150, I228, K255), and APOL1 G2 (EIK haplotype background) cells were generated as previously described (21). Cells were treated with Tet (50 ng/mL) to induce APOL1 expression. The conditionally immortalized HUPEC line was a gift from the Jeffrey B. Kopp lab (National Institute of Diabetes and Digestive and Kidney Diseases, Bethesda, Maryland, USA) (54, 55); 1.7 × 10^9^ virion particles containing *APOL1* G0 (KIK haplotype) or G1 (EIK haplotype) were used to transduce 40,000 cells for 6 to 8 hours, followed by a fresh media change for another 16 to 18 hours. After a total 24 hours of transduction, cells were treated with Tet (2.5 μg/mL) to induce APOL1 expression.

### Generation of APOL1 transgenic mouse model.

A transgenic mouse expressing the human *APOL1 G1* was generated using a low-copy plasmid containing the *APOL1* coding region from BAC CTD-2333M18. The final sequence included 5.1 Kb of upstream and 12.2 Kb of downstream *APOL1* flanking sequence, including the endogenous *APOL1* promoter region. *APOL1* exon 7 was modified to contain the G1 variant mutations (S342G, I384M) in an EIK haplotype background, and the final construct was sequenced to confirm the presence of modifications and the correct coding sequence. Transgenic mice were generated by the Jackson Laboratory in a C57BL/6J background. Founder animals were identified, and the presence of *APOL1* was confirmed by sequencing. Because prior studies of APOL1 in rodent model did not observe any sex-dependent differences in phenotypes (65), in the current study, both male and female mice were used.

To study the effect of VX-147 on APOL1 channel function in vivo, 8-week-old mice were divided into 3 treatment groups: control, IFN-γ, and IFN-γ plus VX-147. Each treatment group included 4 mice. A dose of 1.125 × 10^7^ unit/kg of IFN-γ (BioLegend, 575306) was injected (i.v.) via tail vein at day 0 for the IFN-γ only and IFN-γ plus VX-147 groups. Mice in the IFN-γ plus VX-147 group received a dose of 10 mg/Kg VX-147 twice a day on days 0, 1, and 2 via oral gavage. Mice in the IFN-γ–only group received similar oral gavage of the vehicle solution (10% DMSO/60%PEG400/30% water). Urine samples were collected every day from each mouse. Blood samples were collected at day 0 and day 3. On day 3, mice were euthanized and kidney tissue was retrieved for histology and analysis by electron microscopy.

### Isolation of mouse kidney glomeruli and culture of primary mouse podocytes.

To isolate mouse glomeruli, we used a modified differential adhesion method (66). Briefly, anesthetized mice were perfused with 1× HBSS. Kidneys were harvested, minced, and digested with collagenase IV (1 mg/mL) in 1× HBSS at 37°C for 15 minutes. Digested tissues were filtered through 100 μm to 75 μm cell strainers, and the filtrate containing glomeruli was collected on the 40 μm cell strainer. Isolated glomeruli were cultured in RPMI 1640 media containing 10% FBS, insulin-transferrin-selenium (ITS), and penicillin-streptomycin for 7 to 9 days on laminin-521–coated plates. Enriched outgrowth of primary podocytes was detached using TrypLE Express (with phenol red; Life Tech). The purity of the primary podocyte was confirmed by podocalyxin immunostaining, and the podocytes were seeded for experiments.

### Measurement of intracellular K^+^ and Ca^2+^ levels.

T-REx-293 cell intracellular K^+^ and Ca^2+^ levels were measured with Icagen XRpro x-ray fluorescence (XRF) as previously described (9) and detailed in Supplemental Methods.

### Measurement of Rb^+^ flux in APOL1 transgenic mouse primary podocytes.

In brief, 25,000 cells were plated on laminin-521–coated 96-well plates and treated with mouse IFN-γ (10 ng/mL) with or without VX-147 (3 μM) for 45 hours. Thereafter, the cells were loaded with Rb^+^, a potassium tracer (rubidium chloride [RbCl], 5 mM) for 3 hours. The cells were washed 3 times with modified 1× EBSS buffer without Ca^+2^ and K^+^ and air dried. The intracellular Rb^+^ content was quantified with XRpro XRF (detailed in Supplemental Methods).

### Real-time measurement of intracellular Na^+^ levels in live cells.

T-REx-293 G1 cell intracellular Na^+^ was measured with fluorescent intracellular Na^+^ indicator ING2 as detailed in Supplemental Methods. To determine an estimate of ING2-bound Na^+^, we treated HEK293 cells with 10 μM gramicidin in the presence of increasing extracellular Na^+^. The resulting plot suggests that expression of APOL1 G1 for 8 hours results in an approximate intracellular concentration (Na^+^) of 40–50 mM (Supplemental Figure 5D).

### Real-time measurement of intracellular Ca^2+^ levels in live cells.

Cytosolic Ca^2+^ was measured with Fluo4-AM or a ratiometric Ca^2+^ indicator, Fura2-AM, as detailed in Supplemental Methods.

### Direct measurement of ER Ca^2+^ levels.

D1ER plasmid, a genetic coded calcium indicator (GCCI) of SR/ER Ca^2+^, was electroporated using a neon electroporation system (Thermo Fisher) into T-REx-293 G1 cells and seeded on a 35 mM MatTeck plate. After treating the cells for 12 hours with Tet (50 ng/mL) or Tet (50 ng/mL) plus VX-147 (3 μM), the plates were mounted to the stage of Nikon TE2000-E inverted microscope and continuously perfused with Tyrode’s solution containing 145 mM NaCl, 5 mM KCl, 10 mM HEPES, 5 mM glucose, 2 mM CaCl_2_, and 0.3 mM NaH_2_PO_4_, 1 mM MgCl_2_, and pH7.4. Cells without treatment served as control. The D1ER was excited at 436 nm with a CoolLed light (CoolLed Corp.). CFP and YFP were filtered with narrow-band FRET filters installed inside a motorized cubic filter wheel and collected with a 250 ms delay. Ca^2+^ imaging data were recorded with a pco-Edge 5.0 camera (PCO Corp.), and CFP/YFP ratios were analyzed with Metafluor software, version 7.10 (Molecular Devices). ER Ca^2+^ was released by using a Ca^2+^-free Tyrode solution with 30 μM CPA.

### Real-time measurement of changes in cellular membrane potential in live cells.

PM potential of T-REx-293 G1 cells was measured with FMP dye (Molecular Devices, R8126), as detailed in Supplemental Methods. Our attempts to measure the absolute values of membrane potential with patch clamp in the current clamp mode were unsuccessful due to unstable recording. The cells became less healthy and leaky with APOL1 G1 expression. As an alternate strategy, we generated a calibration curve in HEK293 cells expressing a 2-pore potassium channel. This allows the cell to behave like a K electrode and gives large dynamic range to calibrate the dye (Supplemental Figure 5, A–C). Using this calibration, we calculated the change in membrane potential between uninduced and Tet-induced plus VX-147–treated cells. We found that 8 hours of G1 induction depolarized the cell by 48.5 mV.

### Permeabilized cell respiration.

Respiration assays using permeabilized T-REx-293 G1 cells were performed with the Seahorse Flux Analyzer (Seahorse Bioscience) and a modified version of the creatine kinase (CK) bioenergetic clamp as described previously (67, 68). Additional details are presented in Supplemental Methods.

### Statistics.

All data are represented as mean ± SD. The number of replicates for each experiment is shown in the figure legends. Statistical analysis was calculated using GraphPad Prism 9.0 software. Comparison between groups was performed using 1-way ANOVA with correction for multiple comparisons shown in the figure legends. All *P* values of less than 0.05 were considered statistically significant, as indicated in the text.

### Study approval.

All animal experiments were conducted in accordance with the principles and procedures outlined in the NIH’s *Guide for the Care and Use of Laboratory Animals* (National Academies Press, 2011) and approved by the Institutional Animal Care and Use Committee at Duke University.

### Data availability.

All RNA-Seq data and values for all data points in graphs are reported in the Supporting Data Values file.

## Author contributions

The study was conceived and planned by OAO and SD. Experiments were conducted by SD, BA, NZ, JT, HZ, ABC, GZ, DS, GL, KS, DF, and SN. Data were acquired and analyzed by SD, BA, NZ, JT, HZ, PBR, ABC, DMM, GZ, DF, SEM, JRB, MJM, OI, and OAO. Reagents were provided by TCB and HH. Critical suggestions during the study were provided by DK and CBN. The manuscript was written by OAO and SD. All coauthors edited and approved the manuscript.

## Supplementary Material

Supplemental data

Unedited blot and gel images

Supplemental table 1

Supplemental table 2

Supporting data values

## Version 1 01/16/2024

In-Press Preview

## Version 2 03/01/2024

Electronic publication
