# Supplementary material for: APOL1-mediated monovalent cation transport contributes to APOL1-mediated podocytopathy in kidney disease
Source: J Clin Invest. 2024 Jan 16;134(5):e172262. doi: 10.1172/JCI172262 (PMC10904047; doi:10.1172/JCI172262)
Supplement: Supplemental data [file jci-134-172262-s221.pdf]

Supplemental Materials

Inventory of Supplemental Material

I. Supplemental Methods
II. References
III. Supplemental Figure 1. Status quo and a new conceptual framework.
IV. Supplemental Figure 2. NMR spectra and LCMS data for VX-147.
V. Supplementary Figure 3. VX-147 dose-dependent rescue of APOL1 G1- and G2 induced cytotoxicity.
VI. Supplementary Figure 4. VX-147 does not impact Tet-induced APOL1 mRNA level.
VII. Supplementary Figure 5. Calibration of membrane potential fluorescence and ING-2 fluorescence.
VIII. Supplementary Figure 6. Additional evidence in support of APOL1 G1-induced IP3R signaling.
IX. Supplementary Figure 7. APOL1 G0 does not affect mitochondrial ATP production nor downstream signaling perturbed by APOL1 G1 and G2 in T-REx-293 cells.
X. Supplementary Figure 8. APOL1 G1-induced reduction of intracellular methionine reduces the synthesis of energy carrier, phosphocreatine and its metabolites.
XI. Supplementary Figure 9. Additional evidence of APOL1 G1's impact on regulators of energy metabolism (AMPK) and protein synthesis.
XII. Supplementary Figure 10. APOL1 G1 expression construct
XIII. Supplementary Figure 11: Electron micrograph of APOL1 G1 transgenic mice.

Supplemental Methods

Maintenance of T-REx-293 cell line and human urine-derived podocyte-like epithelial cells (HUPEC).

T-REx-293 **c**ells were maintained under 5% CO_2, at 37°C in DMEM supplemented with 10% (vol/vol) FBS (S10350H, Tet-free; Atlanta Biologicals). 0.2 mg/ml zeocin (ant-zn-1; InvivoGen), 2mg/ml blasticidin (ant-bl-1; InvivoGen), and 1% antibiotic Penicillin-Streptomycin (10,000 U/mL) (15140122; Thermo). HUPEC cells were maintained under 5% CO_2, at 33°C in Advanced RPMI 1640 Medium supplemented with 10% (vol/vol)

FBS (S10350H, Tet-free; Atlanta Biologicals), and 1% antibiotic Penicillin-Streptomycin (10,000 U/mL) (15140122; Thermo).

Realtime Measurement of Intracellular Na^+ Levels in live cells.

In brief, T-REx-293-G1 cells were grown on 96-well and treated as described above. Culture media was removed, and cells were loaded with 4μM ING-2 (Ion Biosciences # 2011C, first diluted in equal volume of Pluronic F127 (Invitrogen # P3000MP) then further diluted to volume in Earle's Balanced Salt Solution: 137mM NaCl, 5.4mM KCl, 1.8mM $CaCl_2$, 0.8mM $MgCl_2$, 10mM HEPES, 5mM Glucose, pH = 7,4), for 1 hour at room temperature. To maintain presence of test agents, compounds were included in dye loading buffer matching that of induction media conditions. After dye loading, buffer was removed from the plates and cells were washed once using Earle's Balanced Salt Solution (EBSS). 50μl of EBSS was placed back on cells for imaging. All fluorescence intensity measurements were performed on a Molecular Devices FLIPR TETRA plate reader. Changes in intracellular sodium was recorded for 20 seconds.

Realtime Measurement of Intracellular Ca^{2+} Levels in live cells.

In brief, T-REx-293-G1 cells were grown on 96-well. Culture media was removed, and cells were loaded with 4μM Fluo4-AM or a ratiometric Ca^{2+} indicator, Fura2-AM (Invitrogen # F14202 and F1225, first diluted in equal volume of Pluronic F127 (Invitrogen # P3000MP) then further diluted to volume in Earle's Balanced Salt Solution: 137mM NaCl, 5.4mM KCl, 1.8mM $CaCl_2$, 0.8mM $MgCl_2$, 10mM HEPES, 5mM Glucose, pH = 7,4), for 1 hour at room temperature. To maintain presence of test agents, compounds were

included in dye loading buffer matching that of induction media conditions. After dye loading, buffer was removed from the plates and cells were washed once using a Ca^{2+}-free buffer containing 137 mM NaCl, 5.4mM KCl, 10 mM glucose, 10 mM HEPES (pH7.4), 0.8 mM $MgCl_2$, and 0.5mM EGTA. 50μl of wash buffer was placed back on cells for imaging. All fluorescence intensity measurements were performed on a Molecular Devices FLIPR TETRA and PENTA plate readers. Starting baseline fluorescence was recorded for 20 seconds. After initial baseline recording, Thapsigargin (Sigma # T9033) was applied to cells at a final concentration of 10μM in the Ca^{2+}-free buffer. Release of intracellular calcium was monitored for an additional 5 minutes. To monitor secondary Ca^{2+} influx, calcium was then re-introduced to the extracellular buffer (5mM Ca^{2+} final assay concentration) and subsequent responses were recorded for an additional 5 minutes.

XRpro x-ray fluorescence (XRF) Measurement of Intracellular K^+,Ca^{2+} and Rb^+ Levels.
In brief, APOL1-expressing cells were grown on 96-well plates as described above. Culture media was removed from the plates and cells were washed once quickly using a K^+- and Ca^{2+}-free buffer containing 137 mM NaCl, 10 mM glucose, 20 mM HEPES (pH7.4) and 1 mM $MgSO_4$. All reagents were trace metal grade or the highest quality commercially available. Cells were lysed for 16 hours in 0.1 ml/well of an osmotic lysis buffer containing 2.5 mM Tris-HCl (pH7.4), 0.25 mM Allura Red AC, 1 mM EDTA, 0.1mM YNO_3 and 0.1 mM Na_2CrO_4. After lysis, samples were transferred to 384-well XRpro XRF plates at 40 μl/well and dried by vacuum centrifugation. XRF spectra for photon energies from 1 to 20 KeV were acquired for each well using an instrument equipped with silicon drift detectors, a 30 W Mo x-ray tube, a microfocus X-ray optic and a

programmable stage. Resulting XRF spectra were curve fit to provide element signals in terms of counts. Lysate XRF signals were converted to molar concentrations by comparison of K/Cr, Ca/Cr and Rb/Y XRF signal ratios to KCl_2, $CaCl_2$ and RbCl standard curves measured in lysis buffer dried as described above. Measurements were made for four to six replicates per treatment condition.

Realtime Measurement of Changes in Cellular Membrane Potential in live cells.

In brief, T-REx-293-G1 cells were grown on 96-well and treated as described above. Culture media was removed, and cells were loaded with 1X FMP dye (Molecular Devices # R8126, diluted in Earle's Balanced Salt Solution), for 1 hour at room temperature. To maintain presence of test agents, compounds were included in dye loading buffer matching that of induction media conditions. After dye loading, cell plates were measured for changes in resting membrane potential. All fluorescence intensity measurements were performed on a Molecular Devices FLIPR TETRA plate reader. For experiments measuring of VX-147 correction of cellular membrane potential, buffer containing either DMSO alone or VX-147 was applied to the cells and membrane potential fluorescence was measured for an additional 10 minutes. Our attempts to measure the absolute values of membrane potential with patch clamp in the current clamp mode was unsuccessful due to unstable recording. The cells became less healthy and leaky with APOL1 G1 expression. As an alternate strategy, we generated calibration curve in HEK 293 cells expressing a 2-pore potassium channel. This allows the cell to behave like a K electrode and gives large dynamic range to calibrate the dye (Supplementary Figure 5A-C). Using this calibration, we calculated the change in membrane potential between uninduced, Tet-

induced plus VX-147-treated cells. We found that 8 hours of G1 induction depolarized the cell by 48.5mV.

Generation of recombinant adenoviruses.

Recombinant adenoviruses containing either APOL1 G0 (KIK haplotype) or G1 (EIK haplotype) were generated following the MultiSite Gateway® Pro-based modular cloning platform (pMVP) strategy as previously described (1). Briefly, gBlocks (Integrated DNA Technologies, Inc.) comprised of the relevant APOL1 open reading frame flanked by attB4r and attB3r sites at their 5' and 3' ends, respectively, were individually recombined into Gateway® entry vector pDONR-P4r-P3r. The resulting APOL1 entry vectors were sequence-verified, then recombined with entry plasmids IG603 (Addgene #121691) and IR904 (Addgene #121799) into Gateway® destination vector pAd-PL-DEST to generate recombinant adenoviral genomes designed for constitutive co-expression of the EGFP reporter and TetOn transcriptional activator genes and for inducible expression of the relevant APOL1 variant (Supple. Fig. 10). Recombinant adenoviral lysates were generated from these recombinant genome plasmids as previously described (1).

Western Blotting.

Western blotting was performed as we previously described (2). After treatment, cells were washed with PBS followed by cell lysis using 1X cell lysis buffer (10X, #9803; Cell Signaling Technology). Cell lysates were sonicated and protein lysates were collected after centrifugation at 10,000 rpm for 10 minutes at 4°C. Protein concentration was determined by BCA protein assay (Pierce, 23225). Samples were heated for 5 minutes at 100°C in 1X sample buffer containing b-mercaptoethanol, and 20–30 μg protein samples

were resolved using precast gels (Bio-Rad). Separated proteins were transferred from gel to PVDF membrane using Trans-Blot Turbo Transfer System (Bio-Rad). Membrane blocking was done using 5% (wt/vol) nonfat milk (M0842; LabScientific) in 1X TBST (IBB-181; Boston BioProducts) and primary antibodies were incubated overnight at 4°C. The following primary antibodies were used: APOL1 rabbit anti-human [Genentech, 3.1C1&3.7D6, Western blot (WB) 1:5000 (final concentration 0.05 ug/mL)]; APOL1 mouse anti-human [Genentech, 4.17A5; Immunofluorescence (IF) 4 ug/mL)]; Anti-Sodium Potassium ATPase antibody (ab76020,IF 1:500, WB 1:100000,Abcam); GAPDH mouse anti-human (Santa Cruz Biotechnology, sc47724, WB 1:200); Vinculin mouse anti-human (Sigma-Aldrich, V9131; WB 1:200); anti–phospho-eIF2α (Ser51) (rabbit mAb #3398, CST); anti-eIF2α (rabbit, #9722, CST); anti–phospho-p38 MAPK (Thr180/Tyr182) (rabbit mAb#4511, CST); anti–phospho-SAPK/JNK(Thr183/Tyr185) (rabbit mAb #4668, CST); anti–b-actin (mouse, sc-130300, Santa Cruz Biotechnology); anti-LC3A (Rabbit #4599, CST); anti-Phospho-AMPKα (Thr172) (Rabbit mAb #50081, CST); anti-AMPKα (Mouse mAb #2793, CST); anti-Phospho-Tuberin/TSC2 (Ser1387) [Rabbit #5584, CST]; anti-Tuberin/TSC2 (Rabbit mAb #4308, CST); anti- Phospho-mTOR (Ser2448) [Rabbit mAb #5536, CST]; anti-mTOR (Rabbit mAb #2983, CST); anti-Phospho-p70 S6 Kinase (Thr389) [Rabbit mAb #9234, CST]; anti-Phospho-4E-BP1 (Thr37/46) [Rabbit mAb #2855, CST]; Anti-rabbit IP3-receptor 1 and 2 antibodies from Prof. Yule's lab. Anti-Mouse IP3-receptor 3 (BD biosciences; #610312); Anti-phospho-GCN2 (T899) [Rabbit mAb #94668S, CST]; anti-GCN2 (#3302S, CST), anti-phospho-PKR (T451) [#07-886, Millipore-Sigma], anti-PKR (#3072S, CST), anti-phospho-PERK (T980) [Rabbit mAb

#3179S, CST), anti-PERK (Rabbit mAb #3192S) and anti-NEPH1 (Santa Cruz Biotechnology, Inc. sc-373787).

Company-recommended dilutions were used for all the antibodies. HRP-conjugated secondary antibodies (anti-mouse IgG, HRP-linked antibody #7076, anti-rabbit IgG, HRP-linked antibody #7074; CST) were used for 60 minutes at room temperature. The membranes were developed using SuperSignal West Dura Extended Duration Substrate (#34075; Thermo Fisher Scientific) and imaging was done with Bio-Rad ChemiDoc MP Imaging System (As per manufacturer's instructions). Densitometric analysis was performed using Alpha View SA software (ProteinSimple).

Generation of IP3-receptor knock-out line using CRISPR-Cas9 gene editing.

In T-REx-293-G1 cells, CRISPR-Cas9 gene editing was performed to generate IP3-receptor knock-out line. Guide-RNAs targeting IP3-receptors 1,2 and 3 and the Cas9 proteins were purchased from Synthego. After electroporation using neon electroporation system (ThermoFisher), single cells were plated in 96-well plates and multiple single clones were selected. Final confirmation of IP3-receptor knock-out clones were done using western blotting. Antibodies targeting IP3-receptor 1 and IP3-receptor 2 were kindly gifted from Dr. David Yule's lab, University of Rochester (3). Anti-Mouse IP3-receptor 3 antibody was purchased from BD biosciences (#610312).

Puromycin Incorporation Assay.

Puromycin incorporation assay was performed to measure the rate of nascent protein synthesis following different treatment conditions as we previously described (2). Cells were treated for 8 hours, 24/48/72 hours and 48 hours for T-REx-293-G1 cells, HUPEC cells and mouse-glom derived primary podocytes respectively and then incubated 10-20 minutes with puromycin (10 mg/ml). Cells were washed with PBS followed by cell lysis using 1X cell lysis buffer (10X, CST #9803). Rate of protein synthesis was measured by detecting puromycin-incorporated nascent protein in western blot, as discussed above, using Anti-Puromycin Antibody, clone 12D10 (MABE343; Sigma-Aldrich) and Vinculin mouse anti-human (V9131; Sigma-Aldrich).

Cell Surface Protein Biotinylation Assay.

Pierce™ Cell Surface Biotinylation and Isolation Kit (A44390; Thermo) was used to label and isolate cell surface proteins to be measured by western blot. Protocols were performed as per manufacturer's instruction and as we previously reported (4). Cells were treated either with Tet(50ng/ul) or Tet(50ng/ul) plus VX-147 (3uM) for 8 hours in a 10 cm dish (~95% confluency). After treatment, cells were washed with PBS and labelled with EZ-Link™ Sulfo-NHS-SS-Biotin for 10 minutes at room temperature. Cells were then washed with ice-cold 1X TBS (kit provided) and then transferred into 1.5ml tubes for cell lysis using lysis buffer (kit provided) on ice for 30 minutes. 5% of the total cell lysates were collected to be used as input during western blot analysis. Next, the isolation and final elution of labeled cell surface proteins was performed according to kit protocol. Finally, western blot analysis was performed using total cell proteins (5% input) and enriched

biotin-labeled APOL1 proteins from PM (Eluted). Na+/K+ ATPase and Vinculin served as loading controls for eluted and input samples respectively.

Immunofluorescence (IF) Staining.

T-REx-293-G1 cells were plated on 24-well culture plates (50,000 cells/well) and allowed to attach for 18–24 hours. After treatment, PM of live cells was stained using Cyto Painter Cell PM Staining Kit (ab219942, deep red fluorescence; Abcam). Nuclei were stained using NucBlue™ Live ReadyProbes™ Reagent (Hoechst 33342; Thermo R37605). For other staining, cells were washed with PBS and fixed in 4% PFA in PBS for 15 minutes at room temperature. After fixation, cells were washed three times with PBS and blocked in blocking buffer (1% BSA in PBS) for 60 min. Cells were incubated with primary antibodies overnight at 4°C followed by secondary antibodies for 1 hour at room temperature. Finally, cells were mounted with ProLong™ Glass Antifade Mountant with NucBlue™ Stain (P3698; Thermo Scientific). Fluorescent images were captured using an ECHO Revolve Microscope or Zeiss 780 or SP8 upright confocal microscope.

Realtime measurement of Diacylglycerol (DAG) Level.

DAG level was measured using the live cell DAG assay kit (U0300G Green Up DAG; Montana Molecular). Protocols were performed as per manufacturer's instruction. In brief, cells were detached using standard trypsinization protocol. Cells were resuspended in complete culture media followed by cell counting. 50,000 cells were seeded per well of 96-well plate. For each transduction reaction (i.e. one well in a 96-well plate), the transduction solution was prepared by mixing 20 µl of the sensor BacMam stock with 0.6

μl of the 500 mM SB (stock solution of sodium butyrate), and 29.4 μl of the complete culture media, for a total volume of 50 μL. 50 μl of transduction solution was mixed with 100 μl of cell suspension (i.e., 50,000 cells) for each well in a 96-well plate. 150 μl of mix were plated into each well and the plate was incubated at room temperature for 30 minutes followed by overnight incubation in a CO_2 incubator at 37° . The next day, cells were treated with Tet (50ng/ml) only or Tet (50ng/ml) plus VX-147 (3μM) and the plate was loaded into Lionheart FX Automated Microscope (BioTek) for 16 hours to capture mean fluorescence signal from DAG sensor. Uninduced cells served as the control group. For mouse glom-derived podocytes, 20000 cells were plated and treated with mouse Interferon-γ (10ng/ml) along with or without VX-147 (3μM) and continue monitoring fluorescence signal for 35 hours.

*Transmission Electron microscopy***.**

We performed transmission electron microscopy of mouse kidney tissue as we previously described (5). Mouse kidney tissue samples were fixed in Glutaraldehyde 2% Paraformaldehyde 2% (Electron Microscopy Sciences, #16536-06) and washed three times with 0.1 M sodium cacodylate buffer for 15 minutes each. Next, samples were post-fixed in 1.0% OsO4 in 0.10 M sodium cacodylate buffer, washed 3 times for 15 minutes each. After that, samples were dehydrated in 50%, 75%, 95%, 100% acetone sequentially, 3 times for 30 minutes (first), and 1 hour for each of the following two 100% acetone incubations. They were then incubated in a 50/50 mixture of EPON/ACETONE for overnight on a rotator, followed by two changes of 100% resin for x hours each on a rotator. Then, samples were embedded in BEEM capsules and baked for 48 hours at

60°C. They were ultrathin sectioned (60–70 nm) on a Leica EM UC7 ultramicrotome, and sections were stained with 2% uranyl acetate in distilled water for 20 minutes and in Sato's lead citrate for 45 seconds. Finally, stained sectioned were imaged on a JEOL 2100Plus electron microscope, and mages were evaluated by a pathologist blinded to treatment groups.

Measurement of Cytotoxicity and Viability.

Cellular cytotoxicity and viability were measured using MultiTox-Fluor Multiplex Cytotoxicity Assay kit (Promega, G9201) and CellTiter-Glo® 2.0 Assay kit (Promega, G9241). Protocols were performed as we previously reported (2). In brief, T-REx-293 APOL1-G0, APOL1-G1 and APOL1-G2 were plated on 96-well plates (100,000 cells/well) and incubated overnight to allow attachment. After 24 hours of treatment, 20µl of 5X MultiTox-Fluor Reagent were added into each well containing 100µl of media and incubated for 1hour at 37C. Fluorescence measurements (live-cell fluorescence at 400Ex/505Em; dead-cell fluorescence at 485Ex/520Em) were done using SpectramaxM3 plate reader (Molecular Devices). After fluorescence measurement, 120µl of CellTiter-Glo reagent was added and mixed thoroughly, and measurement of the luminescent signal was performed using SpectramaxM3 plate reader (Molecular Devices). CellTiter-Glo® 2.0 assay was used to determine the number of viable cells in each well by quantitating the amount of ATP present, which indicates the presence of metabolically active cells. Difference in measures was determined by Student's unpaired t-test.

Clonogenic Survival Assay.

Clonogenic survival assays were performed, to determine the ability of cells to survive and proliferate to form a large colony in presence or absence of certain agents. Protocols were performed as we previously reported (2). Cells were plated on six-well culture dish (400 cells per well) and allowed to attach for 18–24 hours before treatment start. There were three wells (Triplicates) for each treatment condition. Media were changed every 24 hours and cells were maintained for 10–12 days until visible colonies showed up in the control (wells without any treatment). Live cells' nuclei were stained using NucBlue™ Live ReadyProbes™ Reagent (Hoechst 33342; Thermo R37605) and the cell colonies were imaged and counted using Lionheart FX Automated Microscope (BioTek).

Measurement of Real-Time ATP Production Rate and ADP/ATP Ratio.

Agilent Seahorse XFp Real-Time ATP Rate Assay Kit (103591–100; Agilent) was used to simultaneously measure both glycolytic and mitochondrial ATP production rate in live cells as we previously described (2). ADP/ATP Ratio Assay Kit (MAK135, Sigma-Aldrich) was used to measure the levels of ADP and ATP in cells as we previously described (6). Briefly, 100,000 cells/well were plated on Seahorse XF24 cell culture microplates (102340-100; Agilent) in DMEM with 10%FBS (Tet-free) and incubated over night at 37°C with 5%CO2. Next day, the culture media was replaced with fresh media with or without treatment condition for different time points. At the respective timepoints, cells were washed twice with pre-warmed XF assay modified DMEM medium (supplemented with 10 mM XF glucose, 1 mM XF pyruvate, 2 mM XF glutamine; pH 7.4) and placed in non-CO2 incubation at 37°C for 60 minutes to pre-equilibrate the cells with the assay medium. The medium was replaced with fresh, pre-warmed medium before starting the XF assay and

the ATP production rate were measured according to manufacturer's instructions. Both glycolytic and mitochondrial ATP production rate were analyzed using Agilent Seahorse XF Real-Time ATP Rate Assay Report Generator.

For ADP/ATP Ratio Assay, protocols were performed as per manufacturer's instruction. In brief, cells were plated on 96-well plates (100,000 cells/well) and incubated overnight to allow attachment. Cells were treated with Tet (50ng/µl) only or Tet (50ng/µl) plus VX-147 (3µM) for 8, 12 and 24 hours. After treatment, media was removed and 90µl ATP reagent (prepared using kit provided reagents) were added into each well. Plates were tapped briefly to mix and then incubated for 1 minute at room temperature. Luminescence (relative light units) was measured on a luminometer for the ATP assay (RLU_A). Plates were then incubated for another 10 minutes at room temperature and another read was taken for ATP (RLU_B) which provide the background prior to ADP measurement. Immediately following the reading of RLU_B, 5 µL of ADP reagent (prepared using kit provided reagents) were added to each well and mix by tapping. After 1 minute, luminescence (RLU_C) was read. ADP/ATP ratio was calculated using the formula: ADP/ATP ratio = $(RLU_C – RLU_B) / RLU_A$.

Permeabilized Respirometry

Immediately prior to the assay, treatment media was replaced with Buffer Z (105 mM MES potassium salt, 30 mM KCl, 10 mM KH_2PO_4, 5 mM $MgCl_2$) supplemented with fatty acid free BSA (2.5 mg/mL), EGTA (1 mM), Cr (5 mM), PCr (1.5 mM), CK (20U/mL; pH=7.2) and respiratory substrates. At the beginning of assay, cells were permeabilized with 2.0nM XF PM Permeabilizer (PMP; 102504-100; Agilent), followed by the port addition of

5mM ATP. Sequential port additions of PCr were added to achieve final concentrations of 3 and 6mM, which progressively shifts the extra-mitochondrial CK equilibrium towards a lower energetic demand (*i.e.,* increased ATP/ADP ratio). The free energy of ATP hydrolysis (ΔG_{ATP}) after each addition was calculated using the online tool (https://dmpio.github.io/bioenergetic-calculators/ck_clamp/) which uses the equation $\Delta GATP = \Delta GATP^o - 2.3 \ RT*K*2.3*\log \ [(PCr]KCK/[Cr][Pi])]$, where $\Delta GATP^o$ is the standard ΔGATP (−7.592 kcal/mol), R is the gas constant (8.3145 calK−1mol−1), and T is the temperature (310.15 K). The “mix, wait, measure” phase of each measurement cycle consisted of 30 seconds mixing step, 30 seconds wait step, and 2 minutes measurement step. The average oxygen consumption across three measurement periods was used to derive JO_2 values. JO_2 conductance was calculated as the $\Delta JO2/\Delta G_{ATP}$ across the three energetic states. The following substrate combinations were used: 5 mM Pyruvate with 2.5 mM Malate (PM) and α-ketoglutarate (αKG; 5 mM). All data was normalized to cell counts obtained immediately prior to the assay.

Whole-Genome RNA-Sequencing and Ingenuity Pathway Analysis.

T-REx-293-G1 cells were treated with Tet (50ng/ml) only or Tet (50ng/ml) plus VX-147 (3μM) for 8 hours. Untreated cells served as control. RNA was isolated using RNeasy Mini Kit (74106; Qiagen) and sent to Genewiz/Azenta on dry Ice. RNA-seq library preparation and sequencing were performed in Illumina HiSeq4000 as previously described (2).

RNA-seq library preparation and sequencing were performed in Illumina HiSeq4000 following multiple steps, including mRNA enrichment using Oligo-d(T) beads, mRNA

fragmentation & random priming, cDNA synthesis, end repair, 5' phosphorylation & dA-tailing, adaptor ligation, PCR enrichment and sequencing. Sequence reads were trimmed to remove possible adapter sequences and nucleotides with poor quality using Trimmomatic v.0.36. The trimmed reads were mapped to Homo sapiens GRCh38 reference genome available on ENSEMBL using the STAR aligner v.2.5.2b. Unique gene hit counts were calculated using Subread package v.1.5.2. After extraction of gene hit counts, the gene hit counts table was used for downstream differential expression analysis. Comparative gene expression profiles between different groups were performed using DESeq2. The Wald test was used to generate p-values and log2 fold changes. P-value <0.05 and absolute log2fold >1 were called as differentially expressed genes for the comparisons. Ingenuity pathway analysis software (IPA, Qiagen) was used to identify and analyze top canonical signaling pathways altered in-between different groups.

Targeted Metabolomics for organic acids and amines

Cells were seeded on 10cm dishes and after 24-36 hours of incubation, the cells (at >90% confluency) were either not treated (control) or treated with Tet (50ng/ml) and Tet (50ng/ml) plus VX-147 (3μM) for 8 hours. After treatment, cell culture media was collected from each dish for un-targeted metabolomics (discussed below). The cells were washed with ice-cold PBS and 3ml of ice-cold PBS was added; then the cells were scraped off and transferred into a 15ml tube. Next, the cells were spun down at 1,000 rpm for 5 minutes at 4°C and the cell pellet was resuspended in 150μL of ice-cold deionized water containing 0.6% formic acid and 150μL of acetonitrile. Three biological replicate samples were used for each condition.

Organic acids were analyzed by capillary gas chromatography/ mass spectrometry (GCMS) using isotope dilution techniques employing Trace Ultra GC coupled to ISQ MS operating under Xcalibur 2.2 (Thermo Fisher Scientific, Austin, TX) (7). Briefly, 200 µl of cell lysates were spiked with a mixture of heavy isotope labeled internal standards and the keto acids were stabilized by ethoximation. The samples were acidified and the organic acids were extracted into ethyl acetate. The extracts were dried and derivatized with N,O-bis(Trimethylsilyl) trifluoroacetamide. The organic acids were quantified using ion ratios determined from single ion recordings of fragment ions which were specific for a given analyte and its internal standard. These ratios were converted to concentrations using calibrators constructed from authentic organic acids (Sigma, MO, USA).

Amino acids and biogenic amines were analyzed by LC-MS/MS using a modification of a previously published approach (8). 50 µl of cell lysates spiked with a cocktail of heavy isotope-labeled internal standards were deproteinized with methanol and the supernatants were derivatized with the AccQTag (Biosynth-Carbosynth) solution at 55°C for 10 min. Chromatographic separations were performed using a Waters Acquity UPLC system (Milford, MA) and a Waters Acquity UPLC HSS T3 column, 1.8 µm, 2.1 × 100 mm. Injection volume was 2 µL. Mobile phase A was composed of 0.1% formic acid in water. Mobile phase B was acetonitrile. The flow rate was set to 0.6 ml/min and the column temperature was maintained at 40°C. A 6 min gradient method (t=0, %B=0; t=1.0, %B=0; t=6, %B=95) was run followed by a 1 minute wash and 1 minute equilibration. All metabolites were detected in a positive ion MRM mode based on a characteristic fragmentation reaction employing a Waters Xevo TQ-XS mass spectrometer (Milford, MA). Metabolite concentrations were computed using isotope-labeled internal standards

and an external calibration constructed from a serial dilution of the amino acid and biogenic amine standards (Sigma, MO, USA).

Nucleotide analysis

Cells were seeded on 10cm dishes and after 24-36 hours of incubation cells (at >90% confluency) were either not treated (control) or treated with Tet (50ng/ml) and Tet (50ng/ml) plus VX-147 (3μM) for 8 hours. After treatment, all but 3ml media was removed and the cells were scraped off and transferred into a 15ml tube. Next, the cells were spun down at 1,000 rpm for 5 minutes at 4°C; then the cell pellet was washed with ice-cold PBS and resuspended in 300μL of methanol.

Nucleotides were extracted from cell lysates using hexane as described previously (9). Chromatographic separations and mass spec analysis were performed using a Waters Xevo TQ-XS quadrupole mass spectrometer coupled to Acquity UPLC system (Milford, MA) and a Chromolith FastGradient RP-18e 50-2mm column (EMD Millipore, Billerica, MA, USA). Injection volume was 2 μL. Mobile phase A was 95% water, 5% methanol and 5 mM dimethylhexylamine adjusted to pH 7.5 with acetic acid. Mobile phase B was 20% water, 80% methanol and 10 mM dimethylhexylamine. Flow rate was set to 0.3 mL/min and column temperature was maintained at 40°C. A 22 minutes gradient method (t=0, %B=0; t=1.2, %B=0; t=22, %B=40) was run followed by a 3 minutes wash and 7 minutes equilibration. Nucleotides were detected in a negative ion MRM mode based on a characteristic fragmentation reaction.

Measurement of Creatine and Phosphocreatine.

Cells were seeded on 6-well plates and after 24-36 hours of incubation cells (at >90% confluency) were either not treated (control) or treated with Tet (50ng/ml) and Tet (50ng/ml) plus VX-147 (3μM) for 8 hours . After treatment, the cells were washed with ice-cold PBS and 1ml of ice-cold PBS was added; then the cells were scraped off and transferred into a 1.5ml microcentrifuge tube. Next, the cells were spun down at 1,000 rpm for 5min at 4°C and the cell pellet was resuspended in 50μL of 50% methanol. Three biological replicate samples were used for each condition.

Creatine, phosphocreatine, creatinine, and guanidinoacetate were analyzed by LC-MS/MS using a modification of a previously published method (10). Cell lysates prepared in 50% methanol were spiked with heavy-isotope internal standards creatine-d_3 (CDN Isotopes, Canada), creatinine-d_3 (CDN Isotopes, Canada), and guanidinoacetic acid-d_2 (Sigma-Aldrich, MO, USA) and deproteinated with methanol. Chromatographic separations of the methanol supernatants were performed using a Waters Acquity UPLC system (Milford, MA) and a Thermo Hypercarb 100 mm x 2.1 mm, 3 μm column. Injection volume was 2 μL. Mobile phase A was composed of 10 mM ammonium bicarbonate, pH=10 in water. Mobile phase B was acetonitrile. The flow rate was set to 0.4 ml/minutes and the column temperature was maintained at 30°C. A 4 minute gradient method (t=0, %B=0; t=4, %B=60) was run followed by a 1 minute wash and 2 minute equilibration. Creatine, creatinine, and guanidinoacetate were detected in a positive ion MRM mode and phosphocreatine in a negative MRM mode based on a characteristic fragmentation reaction employing a Waters Xevo TQ-XS mass spectrometer (Milford, MA). Metabolite concentrations were computed using an external calibration constructed from a serial dilution of authentic standards and dialyzed fetal bovine serum (Sigma, MO, USA).

Amino acid Tracing.

T-REx-293-G1 cells were seeded in 6 well plates. After 24 hours, cells were washed twice with PBS and treated with Tet (50ng/ml) or Tet (50ng/ml) plus VX-147 (3μM) for 8 hours. For each condition there were three wells of a 6-well plate (triplicate). Cells were incubated for 1 hour in "tracing media" containing "heavy Lysine" (13C6,15N2-L- Lysine). "Tracing media" was prepared using DMEM for SILAC (deficient in both L-lysine and L-arginine; 88364; Thermo) along with 0.7978142mM 13C6,15N2-L-Lysine (CNLM-291-H-0.05; Cambridge isotope lab) and 0.39810428mM unlabeled L-arginine (ULM-8347-0.05; Cambridge isotope lab) were used. After 1hr of labeling, cells were washed 3 times and scraped down using 1.5ml methanol with 20 ul 0.05 mM D4 lysine (as internal standard; DLM-2640-PK; Cambridge isotope lab) into 1.7ml microcentrifuge tube (MCT). A modified GC-MS method was employed for labeled and unlabeled lysine analysis (11, 12). The methanol cell extracts were briefly sonicated for 1 minute followed by centrifugation for 15 minutes at 10,000 g. The supernatants were completely dried by nitrogen gas at 30°C. The dried residues were derivatized with methoxylamine hydrochloride (Alfa Aesar, MA) and N-tert-butyldimethylsilyl-N-methyltrifluoroacetamide (TBDMS) (CovaChem, IL) sequentially. Specifically, 40 μl of methoxylamine hydrochloride (2% (w/v) in pyridine) were added to the dried residues and incubated for 90 minutes at 40°C followed by addition of 20 μL of TBDMS with 1% tert-butylchlorodimethylsilane and incubation for 30 minutes at 80°C. The samples were then centrifuged for 10 minutes at 12,000 × g and the supernatants of derivatized samples were transferred to GC vials for further analysis. GC/MS analysis was conducted using an Agilent 8890 GC system with an Agilent 5977B

Mass Spectrometer (Agilent, CA). Specifically, 1 µl of the derivatized sample was injected into the GC column. The GC temperature gradient started at 80°C for 2 minutes, increased to 280°C at the speed of 7°C per minute, and held at 280°C until the completion of a run time of 40 minutes. The ionization was conducted by electron impact (EI) at 70 eV with Helium flow at 1 mL/minute. Temperatures of the source, the MS quadrupole, the interface, and the inlet were maintained at 230°C, 150°C, 280°C, and 250°C, respectively. Mass spectra (m/z) from 50 to 700 were recorded in mass scan mode. The m/z for unlabeled, [$^{13}C_6$,$^{15}N_2$]L-Lysine, and D4 lysine were 431, 439, and 435, respectively. The retention of lysine was at 26.2 minutes. Mass spectrometry data was recorded and analyzed by Agilent MassHunter and MassHunter Quantitative Analysis software. Refer to supplemental methods for additional details of measurement of labeled and unlabeled lysine in cell lysate.

Un-targeted metabolomics of cell culture medium.

Cell culture media from the targeted metabolomics experiment (described above) were saved for un-targeted metabolomics. Un-targeted metabolomics of cell culture medium was conducted via gas chromatography/electron-ionization mass spectrometry (GC/EI-MS) in the Metabolomics Laboratory of the Duke Molecular Physiology Institute, as described (13, 14). Metabolites in media were extracted by the addition of methanol. Extracts were methoximated, followed by derivatization with *N*-methyl-*N*-trimethylsilyl-trifluoroacetamide (MSTFA), and run on a 7890B GC/5977B MS (Agilent Corporation, Santa Clara, CA), with the MS set to scan broadly from *m/z* 50 to 600 during a GC heat ramp from 60º to 325 ºC. Raw data from Agilent's ChemStation software environment

were reduced with Automatic Mass Spectral Deconvolution and Identification Software or AMDIS (15). Deconvoluted spectra were annotated as metabolites using an orthogonal approach that relies on both retention time (RT) from GC and the fragmentation pattern observed in MS. Peak annotation was based primarily on DMPI's own RT-locked spectral library of metabolites, which is now one of the largest of its kind for GC/EI-MS. Our library is built upon the Fiehn GC/MS Metabolomics RTL Library (a gift from Agilent, part number G1676-90000) (16). Additional spectra for comparison have been gleaned from the Golm Metabolome Library (Max Planck Institute of Molecular Plant Physiology, Golm, Germany) (17) and other public spectral libraries, and by acquiring or synthesizing pure reagent standards and running them.

Mouse urine and blood analysis.

Urine albumin and creatinine levels were measured using Albuwell M Mouse Albumin ELISA kit (Ethos Biosciences, #1011) and Creatinine Companion Assay kit (Ethos Biosciences, #1012) per manufacturer protocols. Blood urea nitrogen (BUN) and serum creatinine were measured by IDEXX BioAnalytics, North Grafton, MA using enzymatic (kinetic) and Alkaline Picrate /modified Jaffe methods respectively.

References

1. Haldeman JM, et al. Creation of versatile cloning platforms for transgene expression and dCas9-based epigenome editing. *Nucleic Acids Res.* 2019;47(4):e23.

2. Datta S, et al. Kidney Disease-Associated APOL1 Variants Have Dose-Dependent, Dominant Toxic Gain-of-Function. *J Am Soc Nephrol.* 2020;31(9):2083-96.

3. Alzayady KJ, et al. Defining the stoichiometry of inositol 1,4,5-trisphosphate binding required to initiate Ca2+ release. *Sci Signal.* 2016;9(422):ra35.

4. Olabisi OA, et al. APOL1 kidney disease risk variants cause cytotoxicity by depleting cellular potassium and inducing stress-activated protein kinases. *Proceedings of the National Academy of Sciences of the United States of America.* 2016;113(4):830-7.

5. Lane BM, et al. Steroid-sensitive nephrotic syndrome candidate gene CLVS1 regulates podocyte oxidative stress and endocytosis. *JCI Insight.* 2022;7(2).

6. Nystrom SE, et al. JAK inhibitor blocks COVID-19 cytokine-induced JAK/STAT/APOL1 signaling in glomerular cells and podocytopathy in human kidney organoids. *JCI Insight.* 2022;7(11).

7. Jensen MV, et al. Compensatory responses to pyruvate carboxylase suppression in islet beta-cells. Preservation of glucose-stimulated insulin secretion. *J Biol Chem.* 2006;281(31):22342-51.

8. Gray N, et al. High-Speed Quantitative UPLC-MS Analysis of Multiple Amines in Human Plasma and Serum via Precolumn Derivatization with 6-Aminoquinolyl-N-hydroxysuccinimidyl Carbamate: Application to Acetaminophen-Induced Liver Failure. *Anal Chem.* 2017;89(4):2478-87.

9. Gooding JR, et al. Adenylosuccinate Is an Insulin Secretagogue Derived from Glucose-Induced Purine Metabolism. *Cell Rep.* 2015;13(1):157-67.

10. Wang JM, et al. Simultaneous determination of creatine phosphate, creatine and 12 nucleotides in rat heart by LC-MS/MS. *J Chromatogr B Analyt Technol Biomed Life Sci.* 2014;958:96-101.

11. Zhang GF, et al. Reductive TCA cycle metabolism fuels glutamine- and glucose-stimulated insulin secretion. *Cell Metab.* 2021;33(4):804-17 e5.

12. Wang Y, et al. Propionate-induced changes in cardiac metabolism, notably CoA trapping, are not altered by l-carnitine. *Am J Physiol Endocrinol Metab.* 2018;315(4):E622-E33.

13. McNulty NP, et al. The impact of a consortium of fermented milk strains on the gut microbiome of gnotobiotic mice and monozygotic twins. *Sci Transl Med.* 2011;3(106):106ra.

14. van Vliet S, et al. A metabolomics comparison of plant-based meat and grass-fed meat indicates large nutritional differences despite comparable Nutrition Facts panels. *Sci Rep.* 2021;11(1):13828.

15. Halket JM, et al. Deconvolution gas chromatography/mass spectrometry of urinary organic acids--potential for pattern recognition and automated identification of metabolic disorders. *Rapid Commun Mass Spectrom.* 1999;13(4):279-84.

16. Kind T, et al. FiehnLib: mass spectral and retention index libraries for metabolomics based on quadrupole and time-of-flight gas chromatography/mass spectrometry. *Anal Chem.* 2009;81(24):10038-48.

17. Kopka J, et al. GMD@CSB.DB: the Golm Metabolome Database. *Bioinformatics.* 2005;21(8):1635-8.

18. Brodney M, et al. In: Organization WIP ed. https://patentsgooglecom/patent/WO2020131807A1/en. USA: Vertex Pharmaceutical; 2019:1-434.

SUPPLEMENTARY FIGURE LEGENDS

Supplementary Figure 1: Status quo and a new conceptual framework.

(A) Graphical summary of existing theories of the mechanisms underlying APOL1-induced cytotoxicity. Nearly a dozen mechanisms have been proposed as mediators of APOL1 RRV-induced cellular injury. However, there is no consensus about the causal connection between these mechanisms. (B) Our proposed conceptual framework for re-imagining the connection between the proximal driver of APOL1 RRV-induced cytotoxicity and its downstream mediators. We hypothesize that inhibition of the proximal causal event would block all downstream cytotoxicity and provide a durable cytoprotection against APOL1 RRV-induced cell death.

Supplementary Figure 2: NMR spectra and LCMS data for VX-147.

(A) ^{1}H NMR spectra and assignments for VX-147. Spectra acquired at 400 MHz frequency in DMSO. (B) HPLC purity profile, SunFire C18 5um 4.6x150mm 25C 1.000mlmin 16min; Pump A Mobile Phase A: 0.03%TFA in H2O; Pump B Mobile Phase A: 0.03%TFA in ACN. (C) LCMS data for VX-147. SunFire C18 50*4.6mm 5um 2.6min 2.0ml/min Temperature: 40°C Gradient: 10% B increase to 50% B for 0.40 min, increase to 95 % B within 1.60 min, 95% B for 0.90 min, back to 10% B within 0.01 min. Pump A: 0.1%FA-H2O; Pump B: 0.1%FA-ACN. LC indicates 99.39% purity. (D) Mass Spectra for VX-147. SunFire C18 50*4.6mm 5um 2.6min 2.0ml/min Temperature: 40°C Gradient: 10% B increase to 50% B for 0.40 min, increase to 95 % B within 1.60 min, 95% B for 0.90 min, back to 10% B within 0.01 min. Pump A: 0.1%FA-H2O; Pump B: 0.1%FA-ACN. MS in +ve mode provides $m/z(M+H)^+ = 418$, MS in -ve mode provides $m/z(M-H)^+ = 416$ as base peaks. The reader is referred to reference Brodney et al (18) for synthetic routes and methods.

Supplementary Figure 3: VX-147 dose-dependent rescue of APOL1 G1- and G2 induced cytotoxicity.

(A) T-REx-293-G1 and (B) T-REx-293-G2 cells were either treated with Tet (50ng/ml) only or Tet (50ng/ml) plus VX-147 in 96-well microplate. Cells with no treatment serve as controls. Different concentrations of VX-147 (1nM, 10nM, 3µM, and 10µM) were used to determine the optimal dose that completely rescued APOL1-induced cytotoxicity. MultiTox-Fluor Cytotoxicity Assay was performed at 24 hours (n = 12 for control and Tet; n = 3 for VX-147 conditions). All data are presented as mean ± SD.

****, P ≤ 0.0001. Ordinary one-way ANOVA with Tukey’s multiple-comparison test.

Supplementary Figure 4: VX-147 does not impact Tet-induced APOL1 mRNA level.

T-REx-293-G1 cells were either treated with Tet (50ng/ml) ± VX-147 for 8 hours followed by extraction of total RNA and quantitation of APOL1 G1 mRNA by Realtime PCR. There is no difference in the levels APOL1 G1 mRNA between Tet-treated vs Tet + VX-147.

Supplementary Figure 5: Calibration of membrane potential fluorescence and ING-2 fluorescence.

Correlation of FMP membrane potential fluorescence (FMP kit Molecular Devices Cat #R8034) changes compared to measured changes in Vm. HEK-293 cells overexpressing a 2-pore K^+ channel were used to calibrate fluorescence changes relative to changes in membrane potential. Shifts in membrane were produced by varying extracellular K^+ concentrations. (A) Current clamp results measuring mV change in membrane potential in response to increasing extracellular K^+ concentrations. Δ-mV calculated relative to basal 5.4mM extracellular K^+. B) Changes in fluorescence in response as a function of extracellular K^+ concentration. Δ-fluorescence calculated relative to 5mM basal K^+ concentration. C) Correlation of Δ-Fluorescence to Δ-mV. $1/\Delta$ correlation plot producing linear fit with R^2 = 0.999. Calibration curve applied to estimate change in membrane potential from fluorescence responses in cells expressing APOL1. (D) To determine an estimate of ING2 bound Na+, HEK cells were treated with 10uM gramicidin in the presence of increasing extracellular Na^+. The resulting plot suggests expression of APOL1 at the 8hr induction timepoint results in an approximate intracellular [Na^+] of 40-50mM. The changes in Na^+ levels are over a much more limited range than that for calcium so the absolute levels of Na^+ remain estimates but are consistent with a depolarized Vm. (E) Live cell cytosolic basal Ca^{2+} levels were measured withFura-2 in T-REx-293 G1 cells co-treated for 12 hours with Tet and VX-147. There is an increase of 200-250nM in internal free Ca2+ following APOL1 G1 induction. VX-147 prevented this rise in Ca^{2+}. ****, P ≤ 0.0001. Ordinary one-way ANOVA with Tukey’s multiple-comparison test.

Supplementary Figure 6: Additional evidence in support of APOL1 G1-induced IP3R signaling.

(A) Ingenuity pathway analysis of RNAseq data from T-REx-293-G1 cells treated or not with Tet (50ng/ml) with or without VX-147 predicts that G1 activates PLC-IP3-mediated

canonical signaling which mitigated in VX-147-treated, G1 expressing cells. (B) The analysis also predicts that xestospongin C would inhibit APOL1 G1-induced IP3-Ca^{2+} signaling. (C) T-REx-293-G1 cells were either treated with Tet (50ng/ml) with or without VX-147(3µM) or different doses of STO-609 (CaM-KK inhibitor; #S8274, Selleckchem) or BAPTA (Membrane-permeable calcium chelator; #S7534, Selleckchem). After 24 hours of treatment, cellular cytotoxicity and viability were measured using MultiTox-Fluor Multiplex Cytotoxicity Assay kit (Promega, G9201). Co-treatment with STO-609 or BAPTA reduced APOL1 G1-induced cytotoxicity. All data are presented as mean ± SD. GraphPad Prism 9 software was used for data analysis. Significance was assessed using unpaired t-test (two-tailed) between Tet (50ng/ml) only and Tet (50ng/ml) plus other inhibitors (n= 6). ****pValue≤0.0001. Ordinary one-way ANOVA with Tukey's multiple-comparison test. (D) Western blot of cell lysate from T-REx-293-G1 cells treated (8 hour) or not with Tet with or without VX-147 shows that induced expression of APOL1 G1 increases phosphorylation of PKC, which was rescued by VX-147 (n= 3). (E) Heatmap showing the gene expression (mean RPKM) of IP3-receptor type 1, 2 and 3 (ITPR1, ITPR2 and ITPR3) from T-REx-293-G1 RNAseq data.
(F) Western blot analysis of T-REx-293-G1 lysate, showing that neither APOL1 G1 expression or its inhibition VX-147 impact protein levels of IP3R1/3 (n= 3). (G) Western blot analysis for the screening of CRISPR-Cas9 edited IP3-receptor knock-out clones. Antibodies against IP3-receptor 1, 2 and 3 (IP3R1, IP3R2 and IP3R3) were used. Anti-IP3R2 antibody did not work. An abbreviated version of this immunoblot is presented in Figure 4I.

Supplementary Figure 7: APOL1 G0 does not affect mitochondrial ATP production nor downstream signaling perturbed by APOL1 G1 and G2 in T-REx-293 cells.
(A, B, C) T-REx-293- EV, G0, and G2 cells were treated or not with Tet ± VX-147 for 24 hours followed by measurement of glycolytic ATP and mitochondrial ATP production rate using Agilent Seahorse XFp Real-Time ATP Rate Assay Kit (n= 5). (D) Western blot analysis showing the levels of phospho-AMPKα (Thr172), phospho-P38, phospho-mTOR (S2448) and phospho-eIF2α in T-REx-293 G0 cells after 8 hours of treatment with Tet ± VX-147. No treatment serves as control. All data are presented as mean ± SD. ***pValue≤0.001, **pValue≤0.005, . Ordinary one-way ANOVA with Tukey's multiple-comparison test.

Supplementary Figure 8: APOL1 G1-induced reduction of intracellular methionine reduces the synthesis of energy carrier, phosphocreatine and its metabolites.
(A) Schematic diagram depicting how reduced methionine level, caused by APOL1 G1, leads to reduced production of creatine and energy carrier, phosphocreatine. In the context of reduced methionine, the methylation of guanidinoacetate to creatine is reduced. Methionine adenosyl transferase, MAT; Guanidoacetate methyltransferase (GAMT). (B) LC-MS/MS analysis of cellular energy carriers. Tetracycline (50ng/ml) induced expression of APOL1 G1 and G2 for 8 hours is associated with reduced levels of creatine, phosphocreatine and creatinine. Expression of G0 did not affect these metabolites. Production of creatine precursor, guanidinoacetate, was unaffected by G1 and G2 expression. Ratio of (Tet) treatment to no treatment control were used for the bar

diagram. *pValue≤0.05. Ordinary one-way ANOVA with Tukey's multiple-comparison test.

Supplementary Figure 9: Additional evidence of APOL1 G1's impact on regulators of energy metabolism (AMPK) and protein synthesis.

Ingenuity pathway analysis of RNAseq data from T-REx-293-G1 cells treated or not with Tet (50ng/ml) with or without VX-147 predicts (A) that APOL1 G1 impacts amino acid transport, and (B) that APOL1 G1 activates AMPK, inhibit MTOR, augment autophagy and inhibit protein synthesis—all predicted to be reversed by VX-147. (C) T-REx-293-G1 cells were treated with Tet with or without plus Dorsomorphin (5µM/10µM) or Dorsomorphin only (5µM/10µM). After 24 hours of treatment cellular cytotoxicity and viability were measured using MultiTox-Fluor Multiplex Cytotoxicity Assay kit (Promega, G9201) and CellTiter-Glo® 2.0 Assay kit (Promega, G9241). Ratio of cytotoxicity and viability was used to represent actual toxicity to cells from each treatment condition. (D)Western blot analyses shows that Co-treatment with Dorsomorphin also reduced pAMPK (pP38and LC3A-II) caused by APOL1 G1.(E) . Puromycin incorporation assay shows that Dorsomorphin rescued protein synthesis blocked by APOL1 G1. Rate of protein synthesis was measured by detecting puromycin-labelled nascent proteins in western blot [anti-puromycin antibody (MABE343; Sigma)]. No treatment serves as control. Densitometric quantification of the western blot protein data is shown by Bar-diagram. (F) T-REx-293 G1 cells were treated with Tet (50ng/ml) for 8 hours. Intracellular (from cell lysate; Targeted metabolomics) and extracellular (from media; Un-targeted metabolomics) levels of glutamic acid, beta-amino-isobutyric acid and gamma-amino-n-butyric acid from the same experiment were compared. (G). Western blot analyses showing the level of phospho-GCN2 (T899), total GCN2, phospho-PKR (T451), total PKR, phospho-PERK (T980) and total PERK in T-REx-293 G1 cells after 8 hours of treatment with Tetracycline (50ng/ml) only or Tetracycline (50ng/ml) + VX-147(3µM). No treatment serves as control. (H-Z) TCA cycle intermediate labeling derived from ^{13}C lysine and the changes of amino acids under treatments of Tetracycline and VX-147. (H) The labeling of citrate isotopomers derived from ^{13}C lysine. M1-M6 mean one or more ^{13}C labeled citrate.

I-Z. Amino acid levels under the treatments of Tetracycline and VX-147.

A: Control (i.e., no treatment but labeled 1hr with L-Lysine $^{13}C_6$,$^{15}N_2$).

B: Tet (50ng/ml) treatment for 8 hours and then labeled 1hr with L-Lysine $^{13}C_6$,$^{15}N_2$. C: Tet (50ng/ml) plus VX-147 (3uM) for 8 hours and then labeled 1hour with L-Lysine $^{13}C_6$,$^{15}N_2$, in presence of 3µM VX-147.

D: Tet (50ng/ml) for 8 hours and then labeled 1 hour with L-Lysine $^{13}C_6$,$^{15}N_2$, in presence of 3µM VX-147.

All data are presented as mean ± SD. GraphPad Prism 9 software was used for data analysis. ****pValue≤0.0001, ***pValue≤0.001, **pValue≤0.005, *pValue≤0.05. One-way ANOVA with Tukey's multiple-comparison test

Supplementary Figure 10: APOL1 G1 expression construct.

(A) Schematic illustration showing the construction and generation of recombinant adenoviral vector containing APOL1 gene. (B) Coding sequence of APOL1 G0 and G1variants used in the recombinant adenoviral construct. (C) Design of the gBlock used in the construction of the recombinant adenoviral genome.

Supplementary Figure 11: Electron micrograph of APOL1 G1 transgenic mice.
Lower magnification electron micrographs of kidneys from APOL1 G1. (A) treated with PBS + vehicle, control, (B) IFNγ or (C) IFNγ + VX-147 Compared to control mice (A), FNγ-treated mice (B) have focal podocyte foot process effacement (white arrow), microvillous transformation (black arrow), and cytoplasmic shedding (white arrowhead). Treatment with VX-147 (C) rescued the cellular phenotypes. The order here is the same as in Figure 10. Bar = 400 nm.

Supplementary Figure 12: Mouse kidney-derived podocytes were stained using antibodies targeting podocyte markers, podocin and synaptopodin.

Supplementary Figure 1.

A

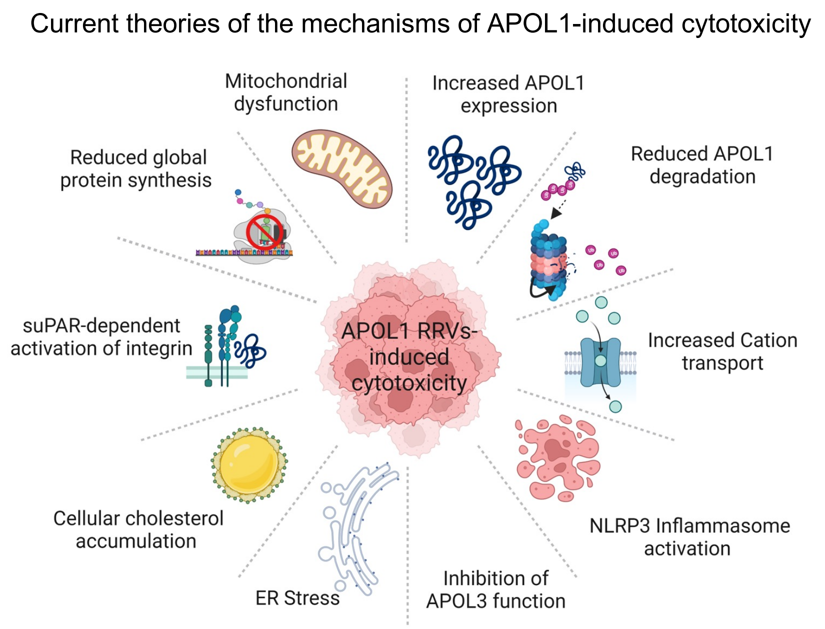

B.

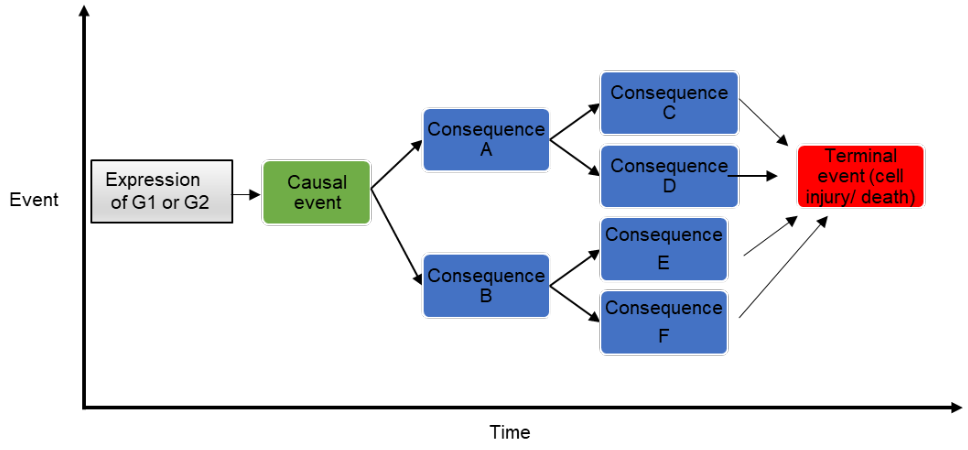

Supplementary Figure 1: Status quo and a new conceptual framework.

(A) Graphical summary of existing theories of the mechanisms underlying APOL1-induced cytotoxicity. Nearly a dozen mechanisms have been proposed as mediators of APOL1 RRV-induced cellular injury. However, there is no consensus about the causal connection between these mechanisms. (B) Our proposed conceptual framework for re-imagining the connection between the proximal driver of APOL1 RRV-induced cytotoxicity and its downstream mediators. We hypothesize that inhibition of the proximal causal event would block all downstream cytotoxicity and provide a durable cytoprotection against APOL1 RRV-induced cell death.

Supplementary Figure 2.

A

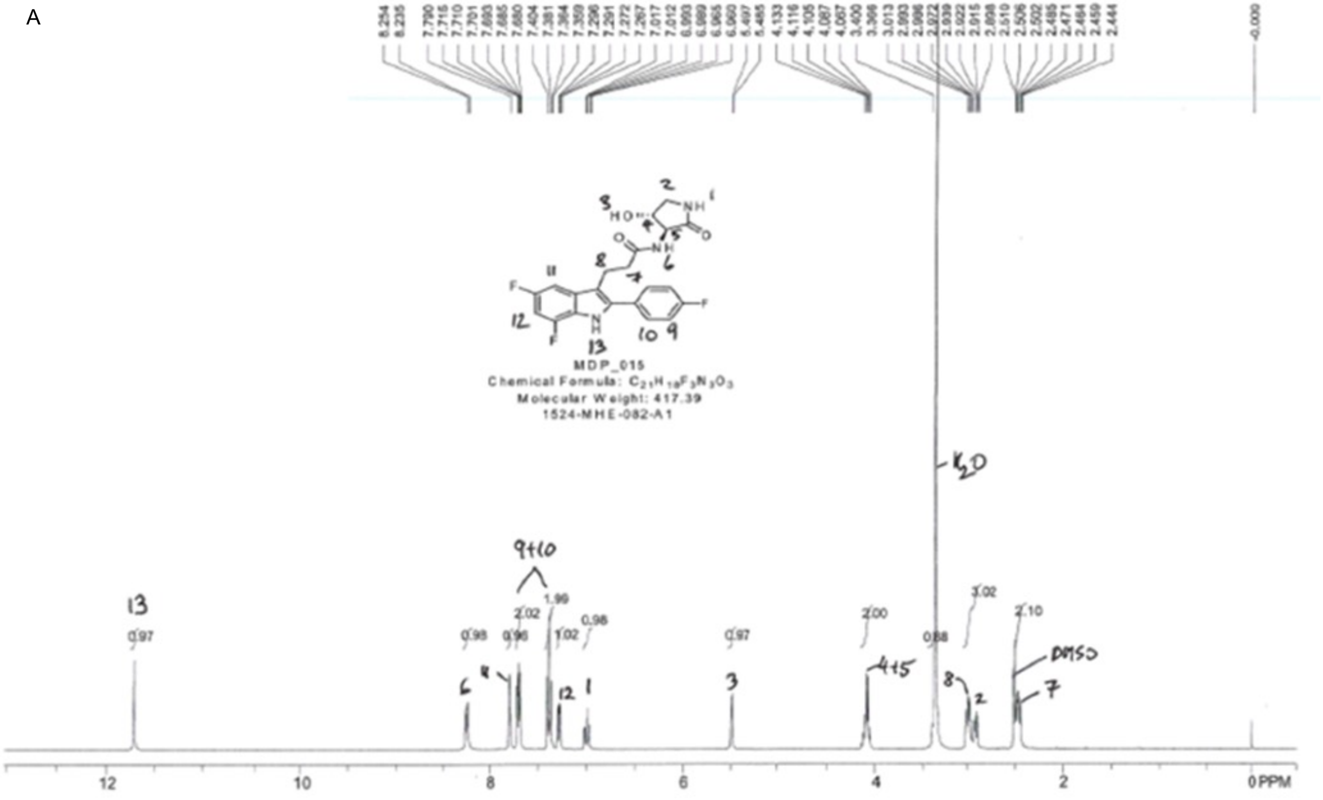

B.

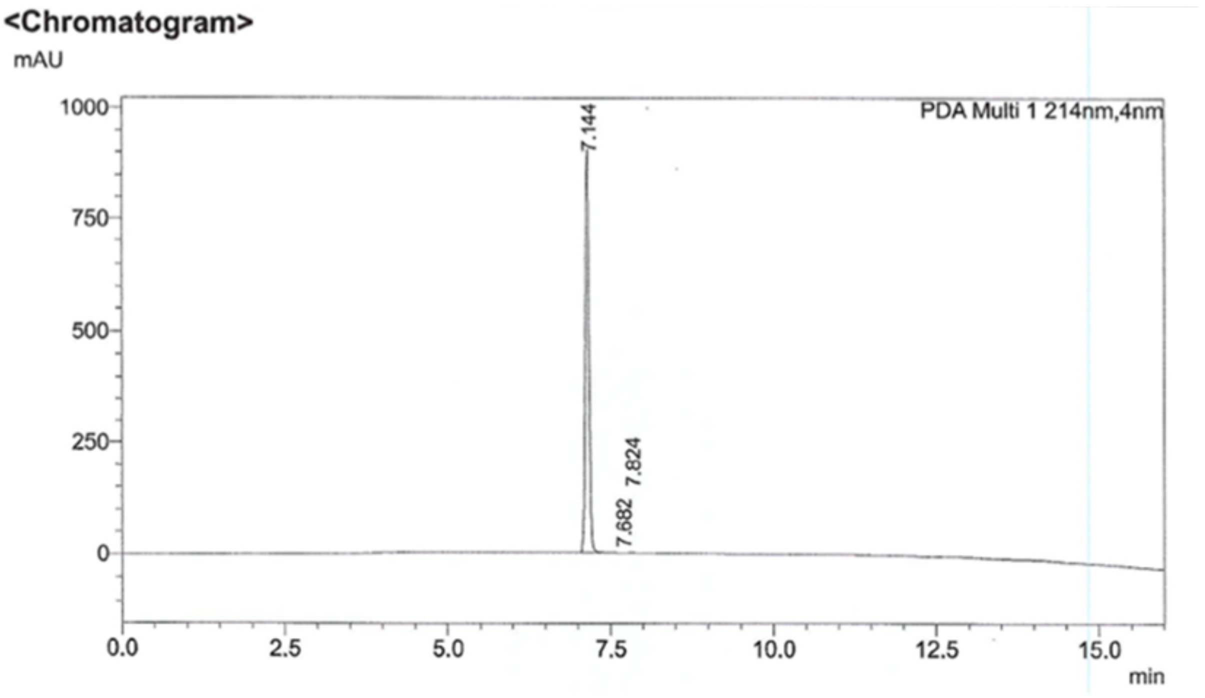

Supplementary Figure 2.

C.

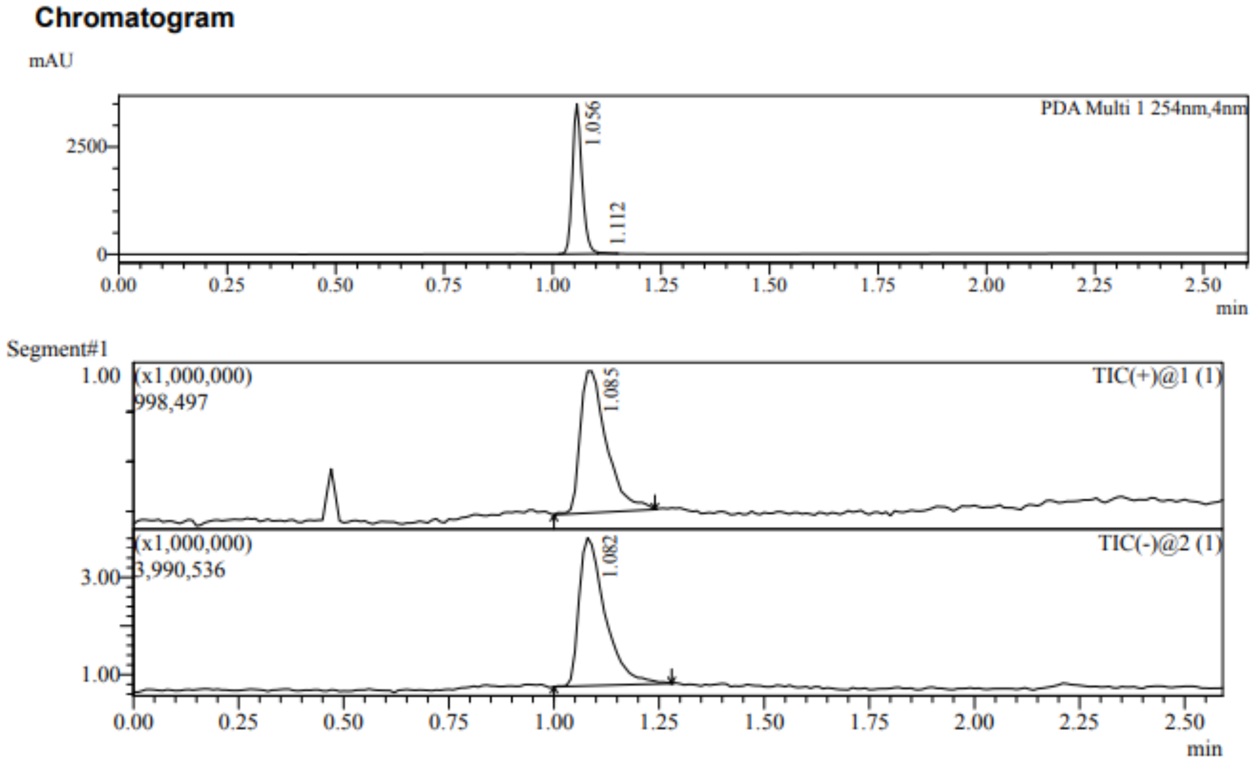

D.

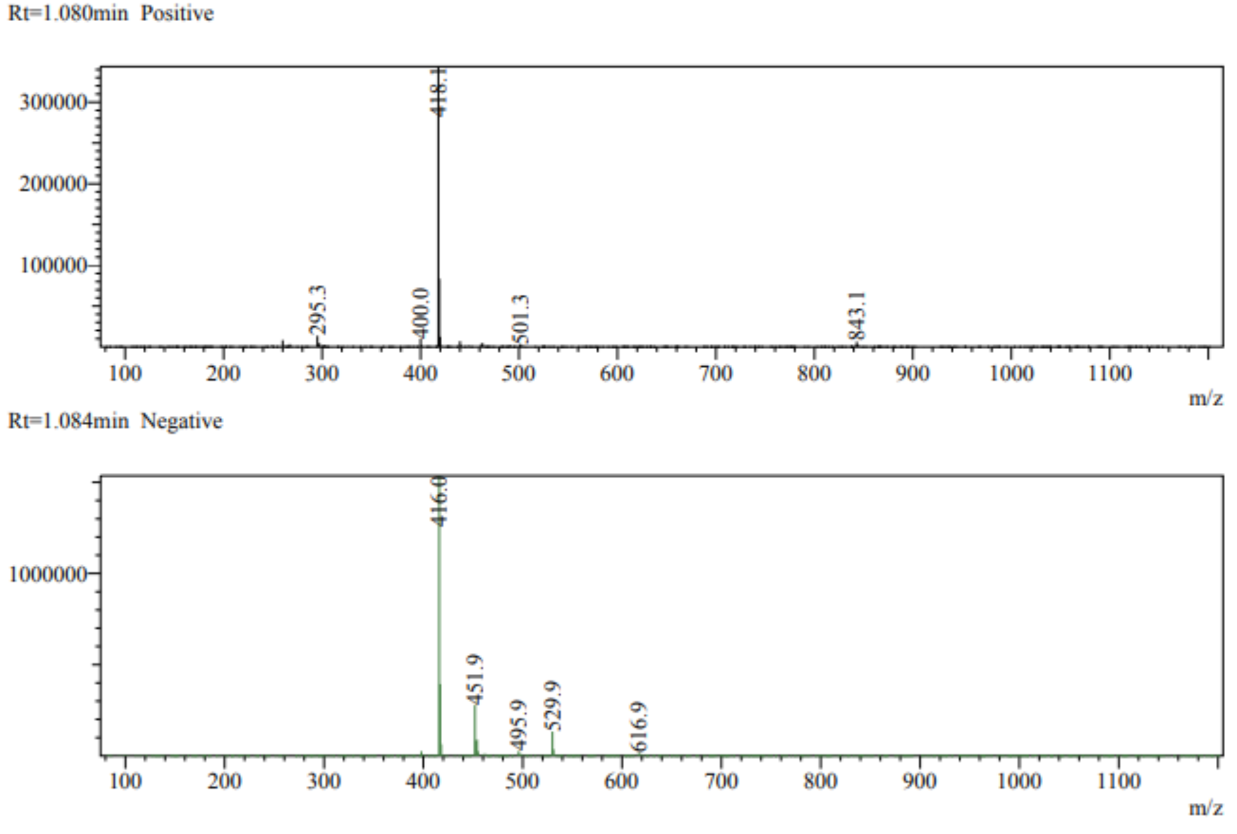

Supplementary Figure 2: NMR spectra and LCMS data for VX-147.

(A) ^{1}H NMR spectra and assignments for VX-147. Spectra acquired at 400 MHz frequency in DMSO. (B) HPLC purity profile, SunFire C18 5um 4.6x150mm 25C 1.000mlmin 16min; Pump A Mobile Phase A: 0.03%TFA in H2O; Pump B Mobile Phase A: 0.03%TFA in ACN. (C) LCMS data for VX-147. SunFire C18 50*4.6mm 5um 2.6min 2.0ml/min Temperature: 40°C Gradient: 10% B increase to 50% B for 0.40 min, increase to 95 % B within 1.60 min, 95% B for 0.90 min, back to 10% B within 0.01 min. Pump A: 0.1%FA-H2O; Pump B: 0.1%FA-ACN. LC indicates 99.39% purity. (D) Mass Spectra for VX-147. SunFire C18 50*4.6mm 5um 2.6min 2.0ml/min Temperature: 40°C Gradient: 10% B increase to 50% B for 0.40 min, increase to 95 % B within 1.60 min, 95% B for 0.90 min, back to 10% B within 0.01 min. Pump A: 0.1%FA-H2O; Pump B: 0.1%FA-ACN. MS in +ve mode provides $m/z(M+H)^{+}$ = 418, MS in -ve mode provides $m/z(M\text{-}H)^{+}$ = 416 as base peaks. The reader is referred to reference Brodney et al (18) for synthetic routes and methods.

Supplementary Figure 3.

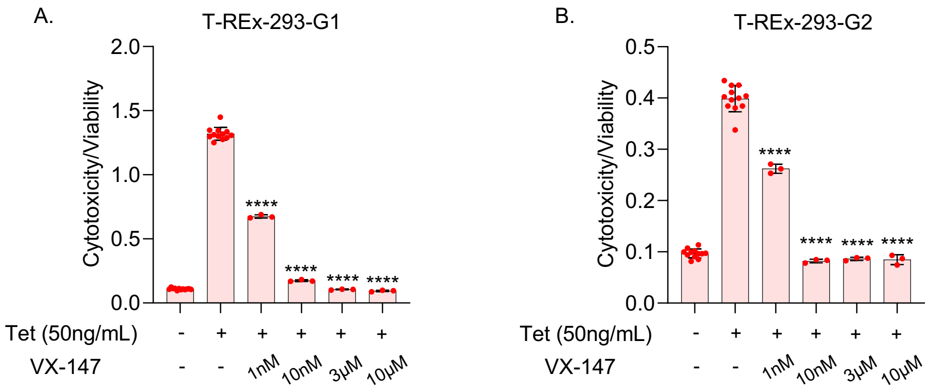

(A) T-REx-293-G1 and (B) T-REx-293-G2 cells were either treated with Tet (50ng/ml) only or Tet (50ng/ml) plus VX-147 in 96-well microplate. Cells with no treatment serve as controls. Different concentrations of VX-147 (1nM, 10nM, 3μM, and 10μM) were used to determine the optimal dose that completely rescued APOL1-induced cytotoxicity. MultiTox-Fluor Cytotoxicity Assay was performed at 24 hours (n = 12 for control and Tet; n = 3 for VX-147 conditions). All data are presented as mean ± SD.

****, P ≤ 0.0001. Ordinary one-way ANOVA with Tukey's multiple-comparison test.

Supplementary Figure 4.

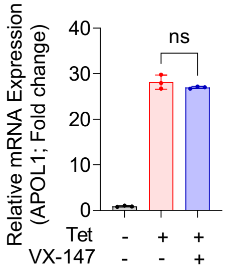

Supplementary Figure 4: VX-147 does not impact Tet-induced APOL1 mRNA level.

T-REx-293-G1 cells were either treated with Tet (50ng/ml) ± VX-147 for 8 hours followed by extraction of total RNA and quantitation of APOL1 G1 mRNA by Realtime PCR. There is no difference in the levels APOL1 G1 mRNA between Tet-treated vs Tet + VX-147.

Supplementary Figure 5.

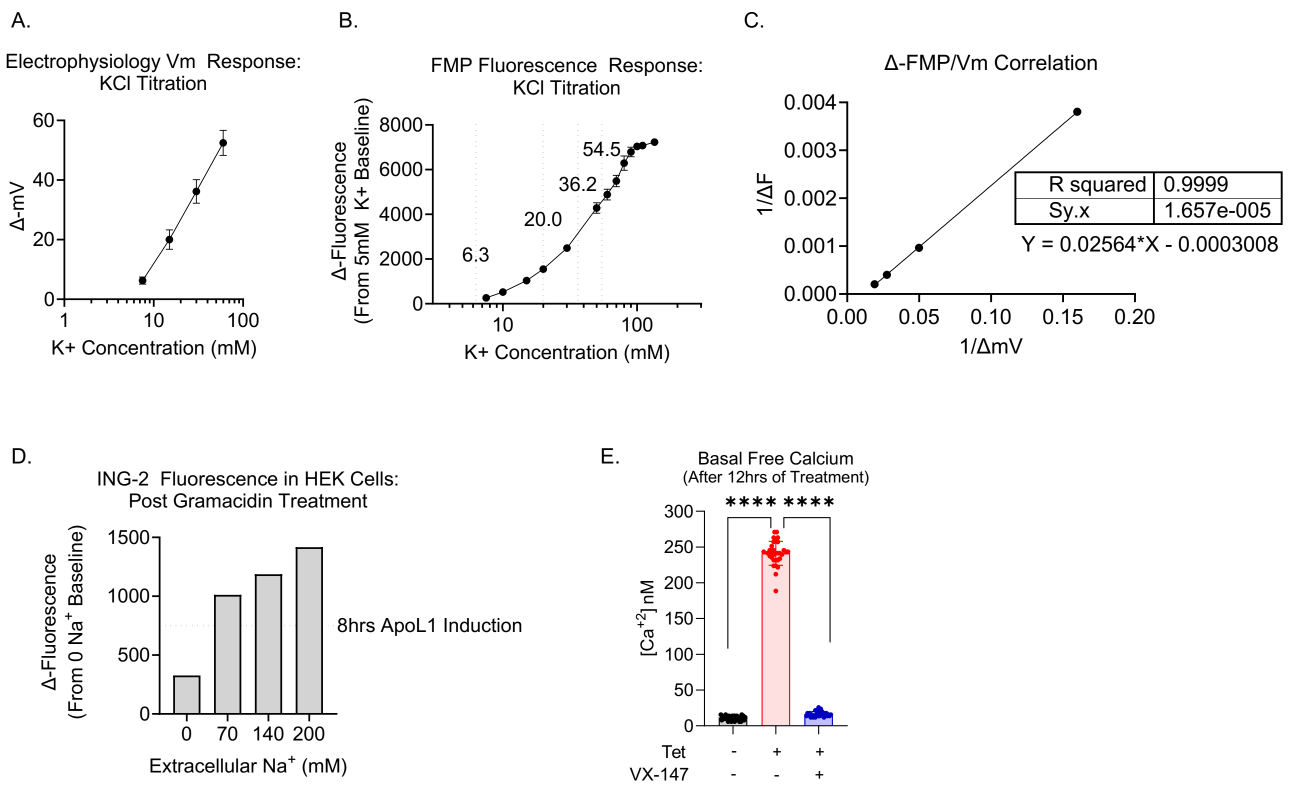

Supplementary Figure 5: Calibration of membrane potential fluorescence and ING-2 fluorescence.

Correlation of FMP membrane potential fluorescence (FMP kit Molecular Devices Cat #R8034) changes compared to measured changes in Vm. HEK-293 cells overexpressing a 2-pore K^+ channel were used to calibrate fluorescence changes relative to changes in membrane potential. Shifts in membrane were produced by varying extracellular K^+ concentrations. (A) Current clamp results measuring mV change in membrane potential in response to increasing extracellular K^+ concentrations. Δ-mV calculated relative to basal 5.4mM extracellular K^+. B) Changes in fluorescence in response as a function of extracellular K^+ concentration. Δ-fluorescence calculated relative to 5mM basal K^+ concentration. C) Correlation of Δ-Fluorescence to Δ-mV. 1/Δ correlation plot producing linear fit with R^2 = 0.999. Calibration curve applied to estimate change in membrane potential from fluorescence responses in cells expressing APOL1. (D) To determine an estimate of ING2 bound Na+, HEK cells were treated with 10uM gramicidin in the presence of increasing extracellular Na^+. The resulting plot suggests expression of APOL1 at the 8hr induction timepoint results in an approximate intracellular [Na^+] of 40-50mM. The changes in Na^+ levels are over a much more limited range than that for calcium so the absolute levels of Na^+ remain estimates but are consistent with a depolarized Vm. (E) Live cell cytosolic basal Ca^{2+} levels were measured withFura-2 in T-REx-293 G1 cells co-treated for 12 hours with Tet and VX-147. There is an increase of 200-250nM in internal free Ca2+ following APOL1 G1 induction. VX-147 prevented this rise in Ca^{2+}. ****, $P \leq 0.0001$. Ordinary one-way ANOVA with Tukey's multiple-comparison test.

Supplementary Figure 6.

A. PLC-IP3-mediated Signaling Pathway

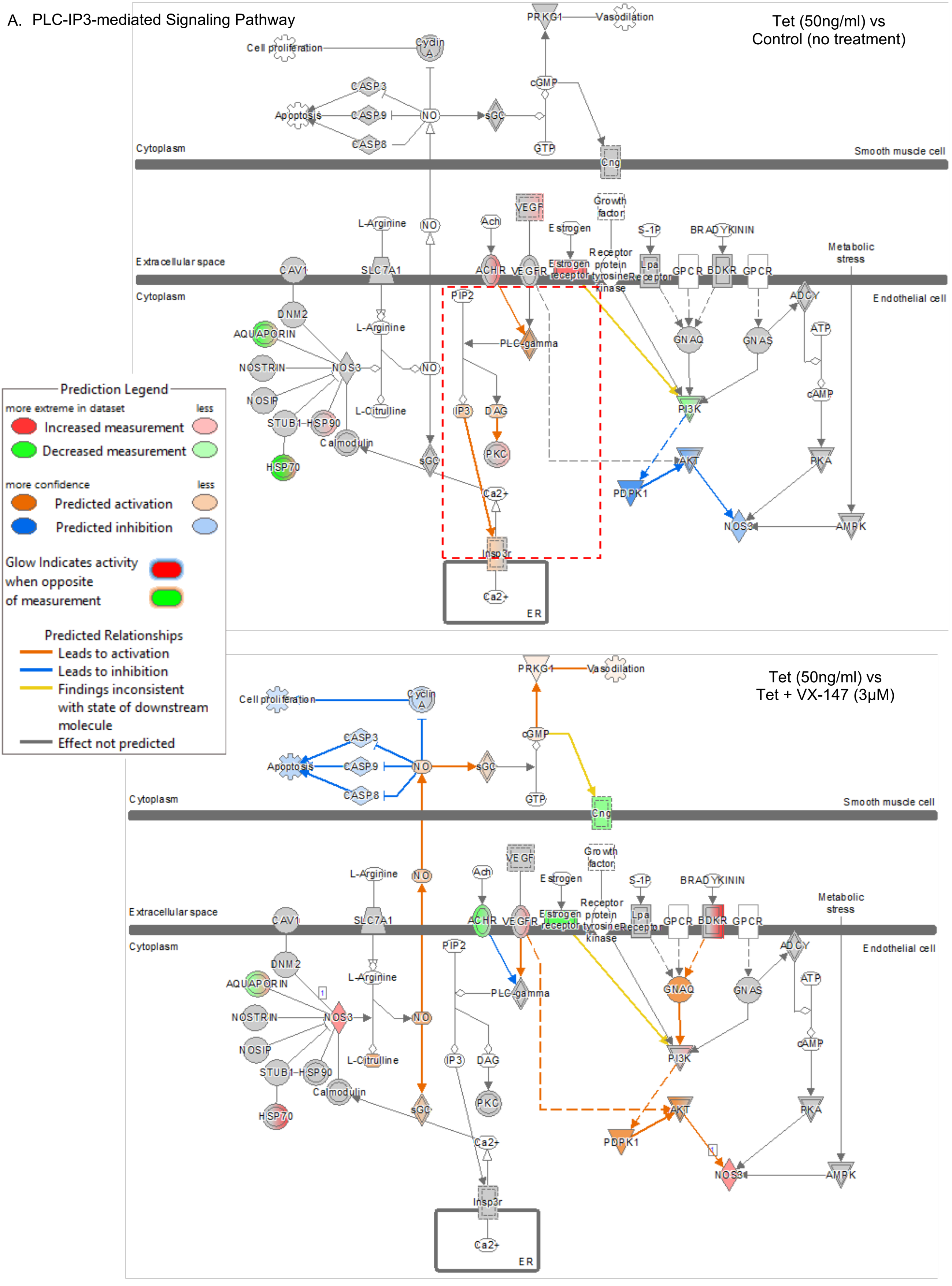

Supplementary Figure 6.

B. Prediction of Xestospongin C mediated inhibition of five signaling pathways (likely downstream of IP3-Ca2+ signaling) induced by APOL1- G1

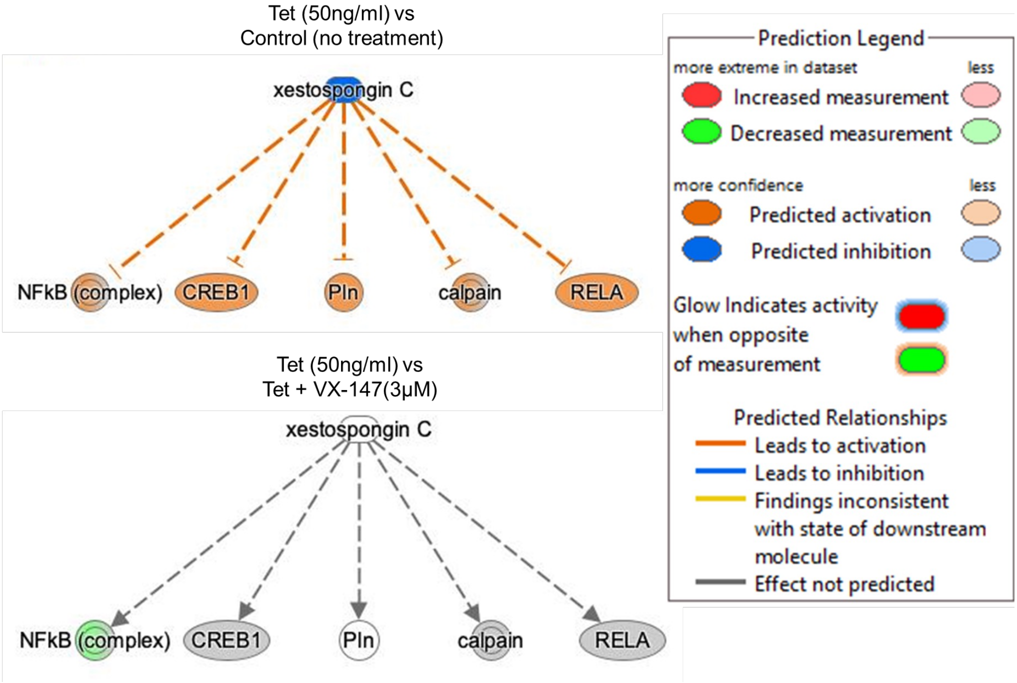

C.

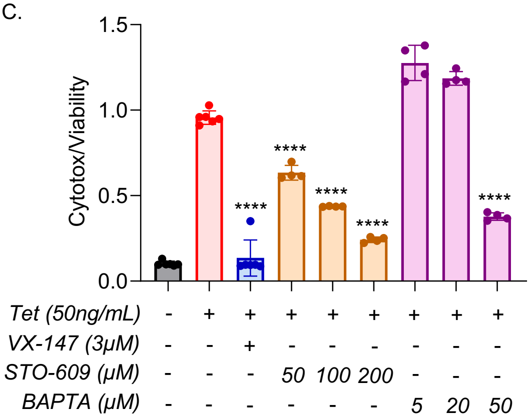

D.

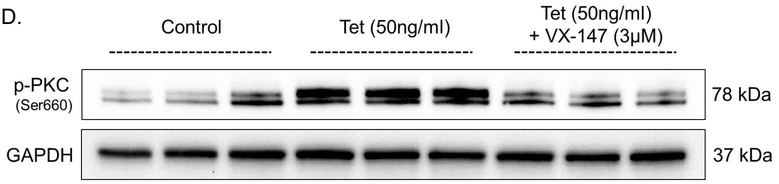

E.

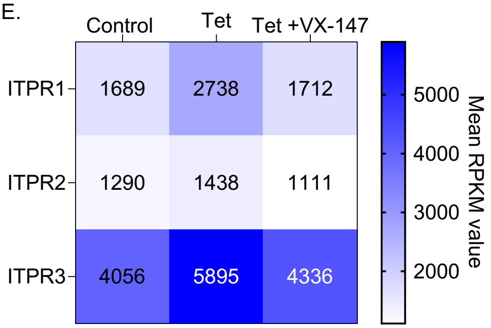

F.

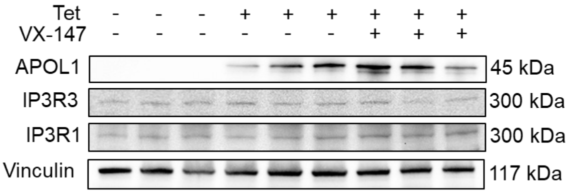

G.

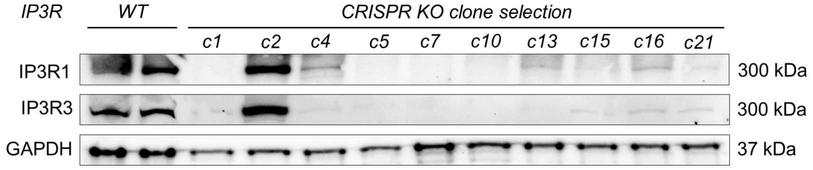

Supplementary Figure 6: Additional evidence in support of APOL1 G1-induced IP3R signaling.

(A) Ingenuity pathway analysis of RNAseq data from T-REx-293-G1 cells treated or not with Tet (50ng/ml) with or without VX-147 predicts that G1 activates PLC-IP3-mediated canonical signaling which mitigated in VX-147-treated, G1 expressing cells. (B) The analysis also predicts that xestospongin C would inhibit APOL1 G1-induced IP3-Ca^{2+} signaling. (C) T-REx-293-G1 cells were either treated with Tet (50ng/ml) with or without VX-147(3μM) or different doses of STO-609 (CaM-KK inhibitor; #S8274, Selleckchem) or BAPTA (Membrane-permeable calcium chelator; #S7534, Selleckchem). After 24 hours of treatment, cellular cytotoxicity and viability were measured using MultiTox-Fluor Multiplex Cytotoxicity Assay kit (Promega, G9201). Co-treatment with STO-609 or BAPTA reduced APOL1 G1-induced cytotoxicity. All data are presented as mean ± SD. GraphPad Prism 9 software was used for data analysis. Significance was assessed using unpaired t-test (two-tailed) between Tet (50ng/ml) only and Tet (50ng/ml) plus other inhibitors (n= 6). ****pValue≤0.0001. Ordinary one-way ANOVA with Tukey's multiple-comparison test. (D) Western blot of cell lysate from T-REx-293-G1 cells treated (8 hour) or not with Tet with or without VX-147 shows that induced expression of APOL1 G1 increases phosphorylation of PKC, which was rescued by VX-147 (n= 3). (E) Heatmap showing the gene expression (mean RPKM) of IP3-receptor type 1, 2 and 3 (ITPR1, ITPR2 and ITPR3) from T-REx-293-G1 RNAseq data.
(F) Western blot analysis of T-REx-293-G1 lysate, showing that neither APOL1 G1 expression or its inhibition VX-147 impact protein levels of IP3R1/3 (n= 3). (G) Western blot analysis for the screening of CRISPR-Cas9 edited IP3-receptor knock-out clones. Antibodies against IP3-receptor 1, 2 and 3 (IP3R1, IP3R2 and IP3R3) were used. Anti-IP3R2 antibody did not work. An abbreviated version of this immunoblot is presented in Figure 4I.

Supplementary Figure 7.

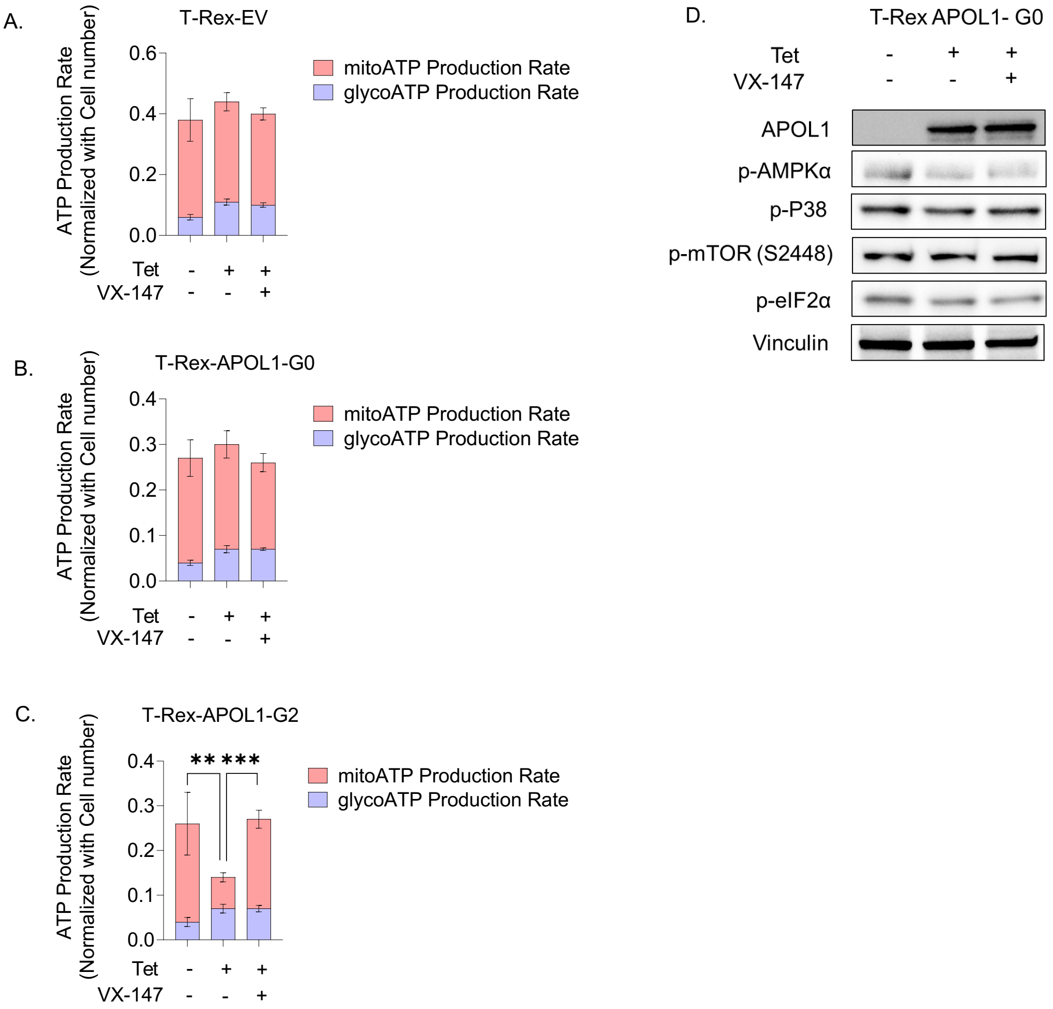

Supplementary Figure 7: APOL1 G0 does not affect mitochondrial ATP production nor downstream signaling perturbed by APOL1 G1 and G2 in T-REx-293 cells.

(A, B, C) T-REx-293- EV, G0, and G2 cells were treated or not with Tet ± VX-147 for 24 hours followed by measurement of glycolytic ATP and mitochondrial ATP production rate using Agilent Seahorse XFp Real-Time ATP Rate Assay Kit (n= 5). (D) Western blot analysis showing the levels of phospho-AMPKα (Thr172), phospho-P38, phospho-mTOR (S2448) and phospho-eIF2α in T-REx-293 G0 cells after 8 hours of treatment with Tet ± VX-147. No treatment serves as control. All data are presented as mean ± SD. ***pValue≤0.001, **pValue≤0.005, . Ordinary one-way ANOVA with Tukey's multiple-comparison test.

Supplementary Figure 8.

A.

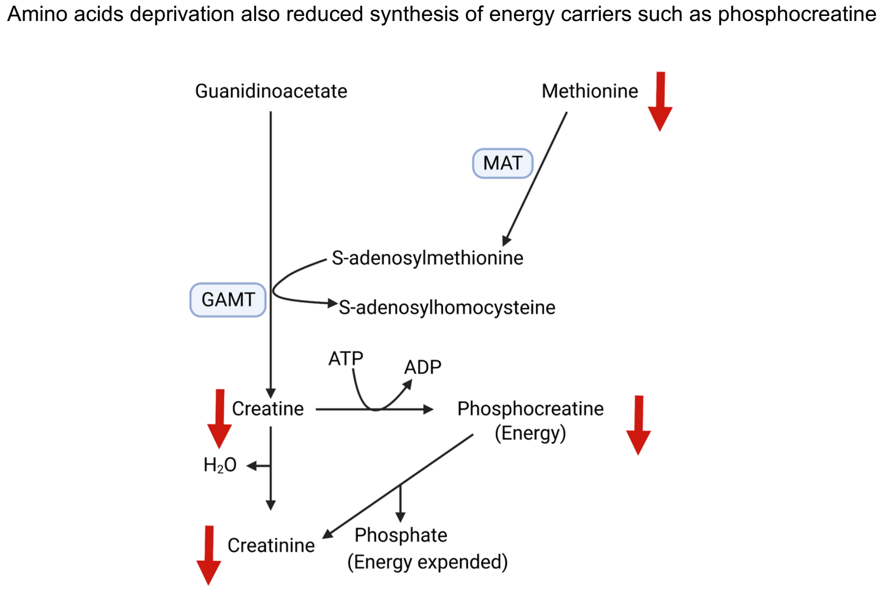

B.

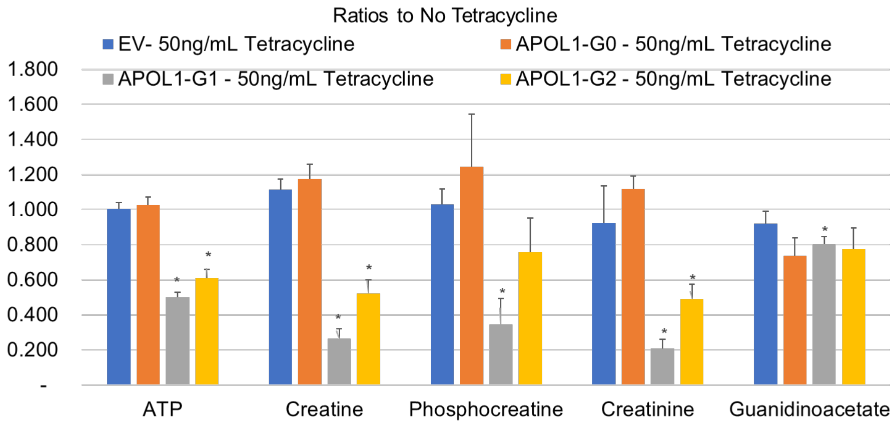

Supplementary Figure 8: APOL1 G1-induced reduction of intracellular methionine reduces the synthesis of energy carrier, phosphocreatine and its metabolites.

(A) Schematic diagram depicting how reduced methionine level, caused by APOL1 G1, leads to reduced production of creatine and energy carrier, phosphocreatine. In the context of reduced methionine, the methylation of guanidinoacetate to creatine is reduced. Methionine adenosyl transferase, MAT; Guanidoacetate methyltransferase (GAMT). (B) LC-MS/MS analysis of cellular energy carriers. Tetracycline (50ng/ml) induced expression of APOL1 G1 and G2 for 8 hours is associated with reduced levels of creatine, phosphocreatine and creatinine. Expression of G0 did not affect these metabolites. Production of creatine precursor, guanidinoacetate, was unaffected by G1 and G2 expression. Ratio of (Tet) treatment to no treatment control were used for the bar diagram. *pValue≤0.05. Ordinary one-way ANOVA with Tukey's multiple-comparison test.

Supplementary Figure 9.

Genes Associated with Amino acids transport and mTOR Signaling

A.

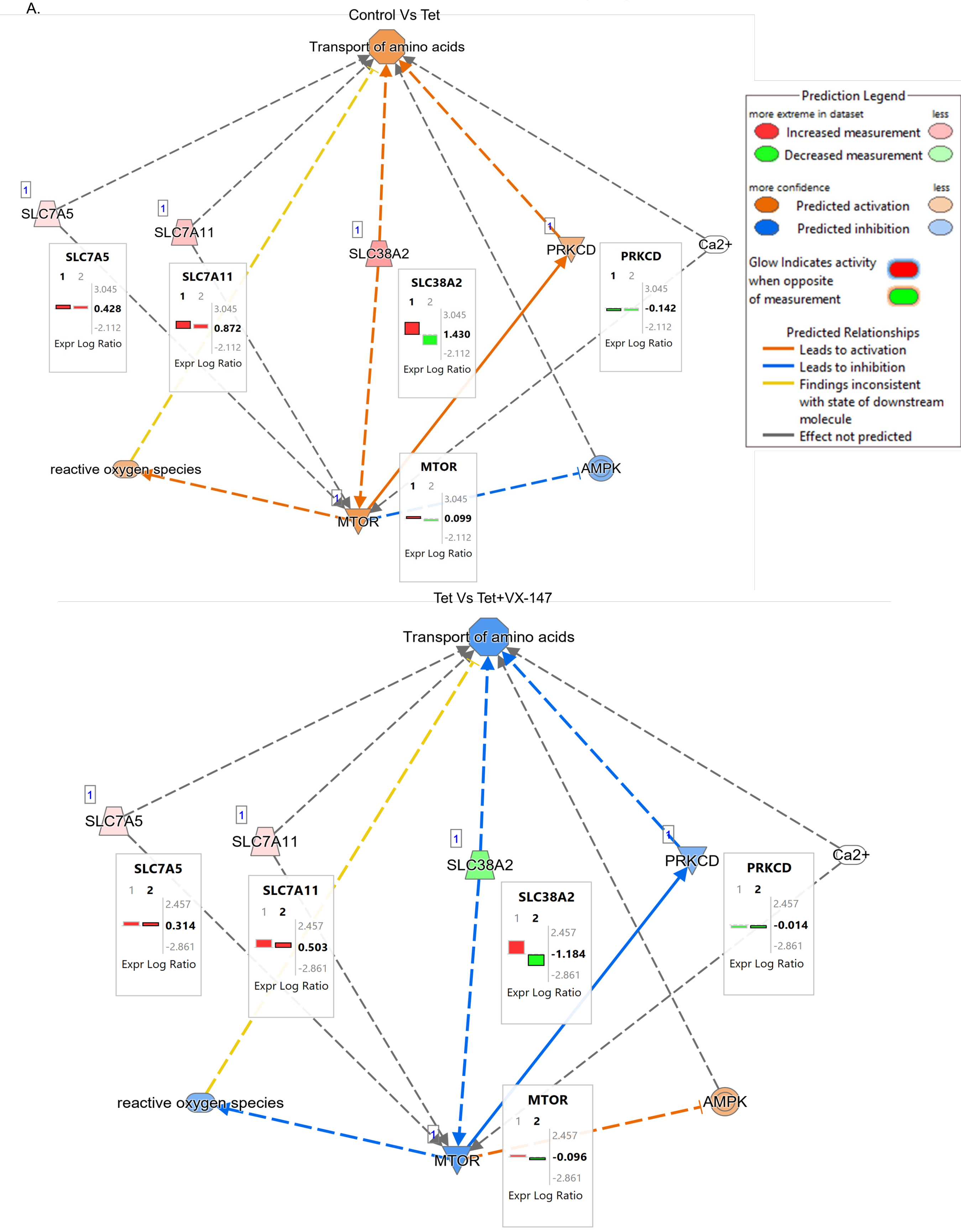

Supplementary Figure 9.

B. Genes Associated AMPKa, Autophagy and Protein synthesis

Control Vs Tet

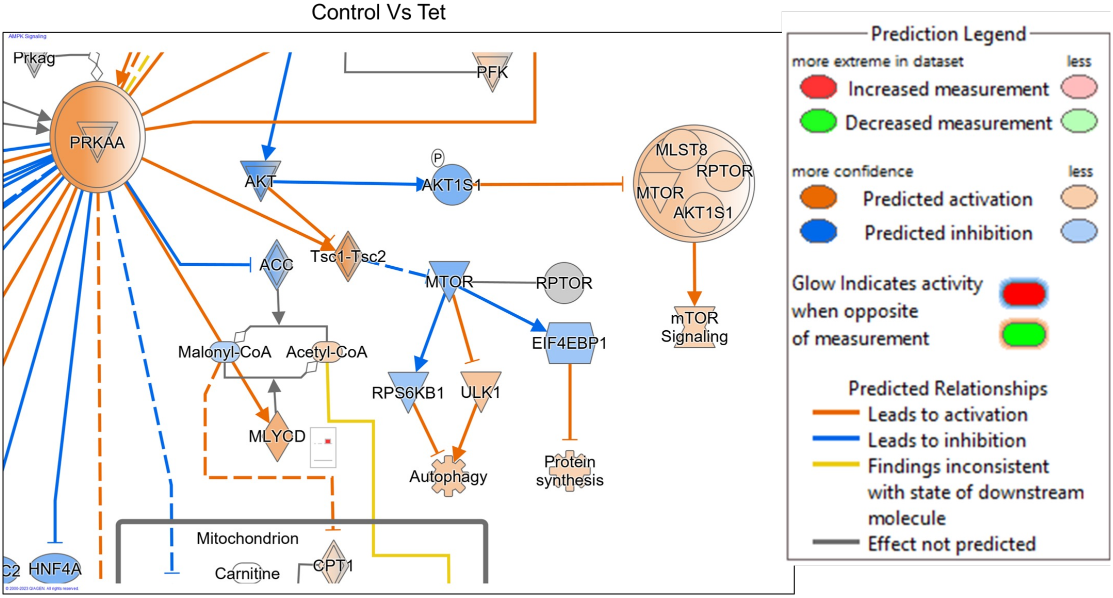

Tet Vs Tet+VX-147

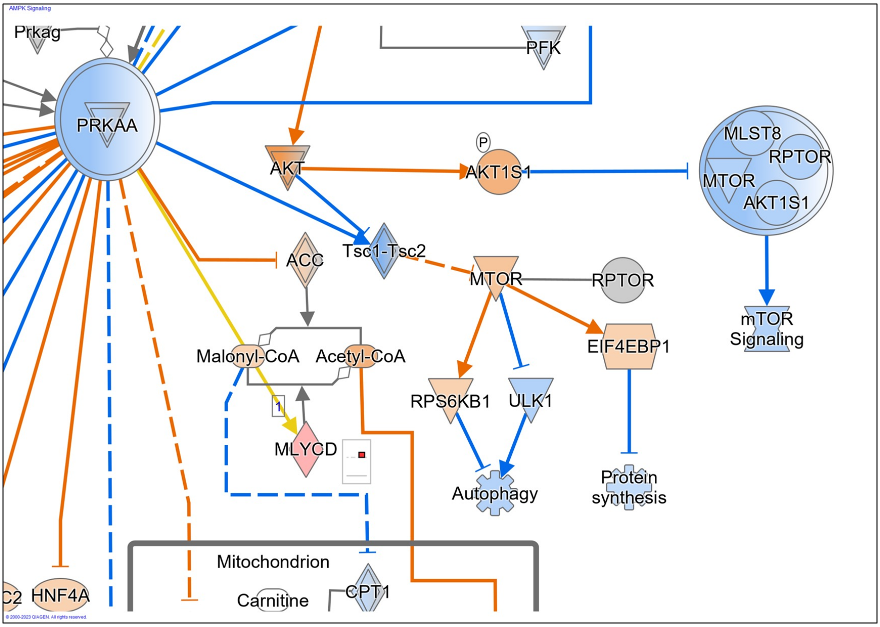

Supplementary Figure 9.

C.

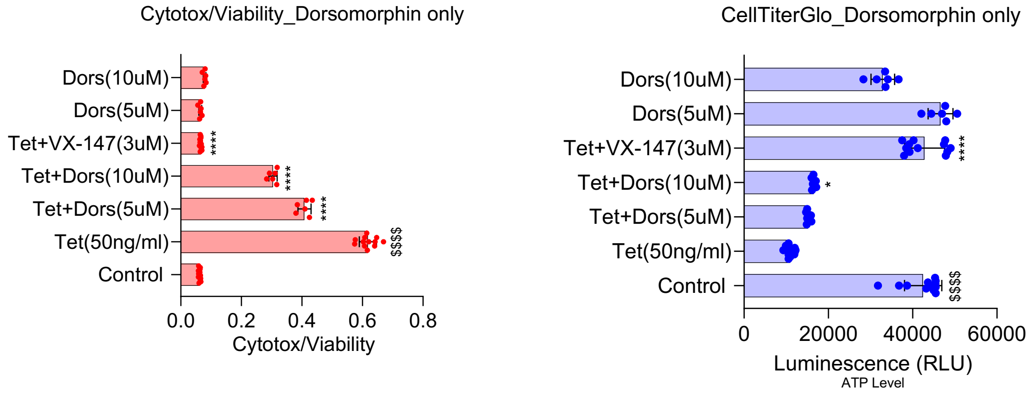

D.

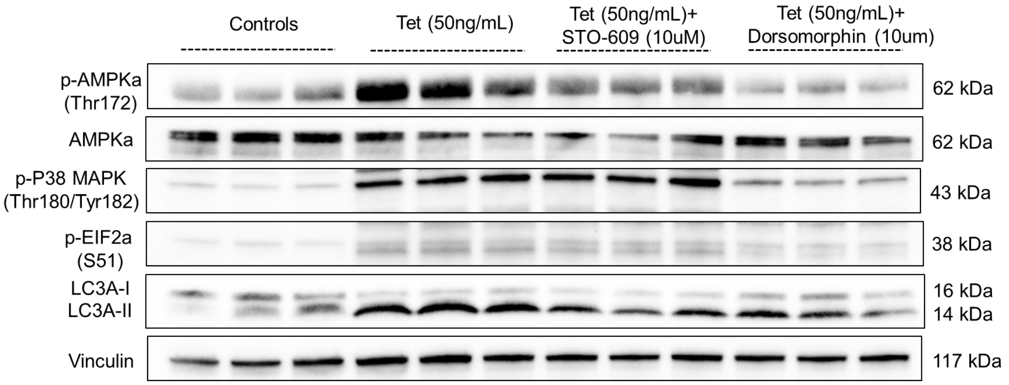

E.

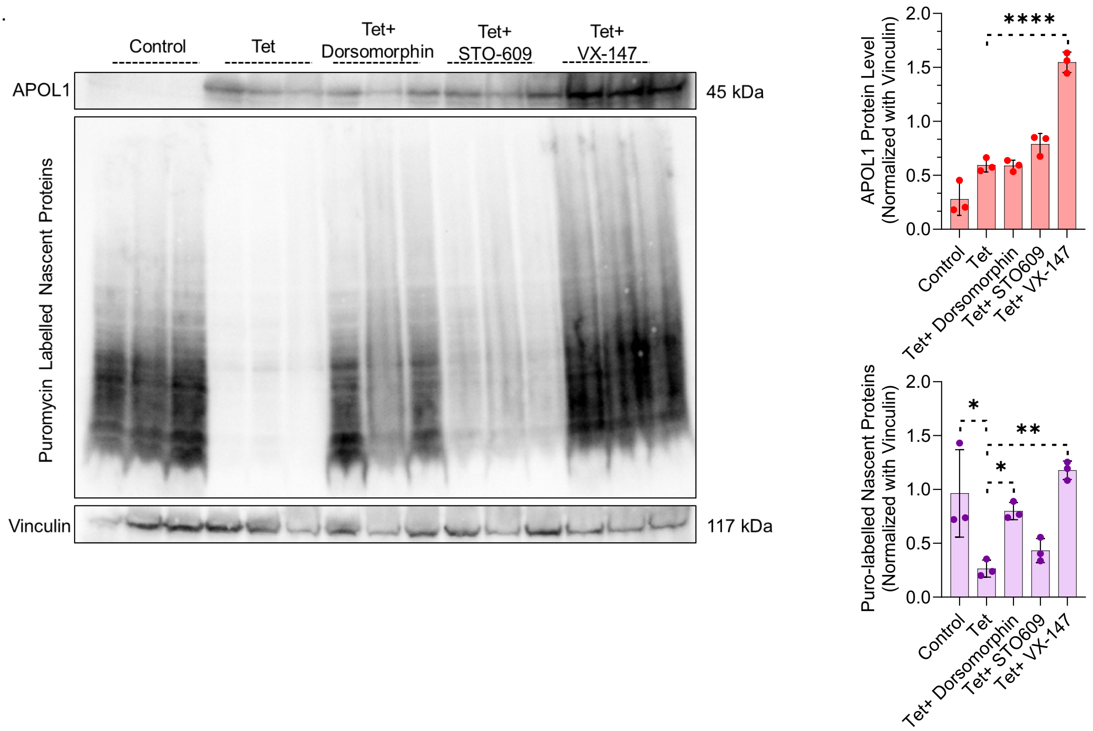

Supplementary Figure 9.

F.

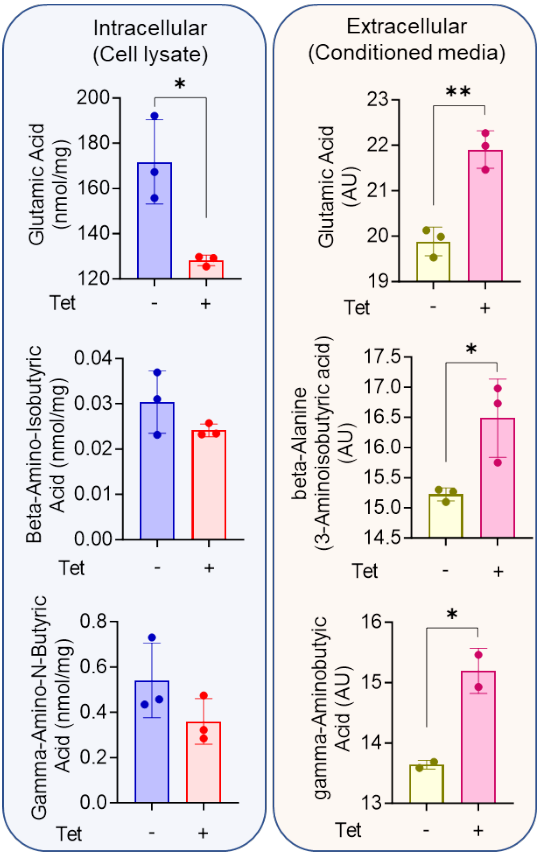

G.

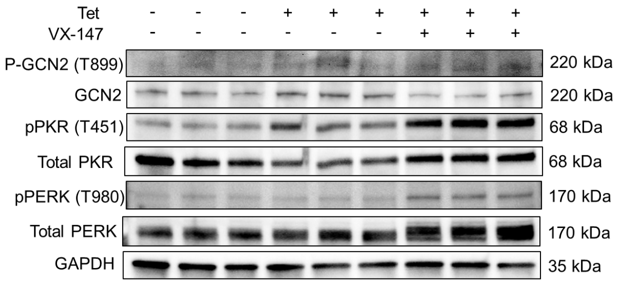

Supplementary Figure 9.

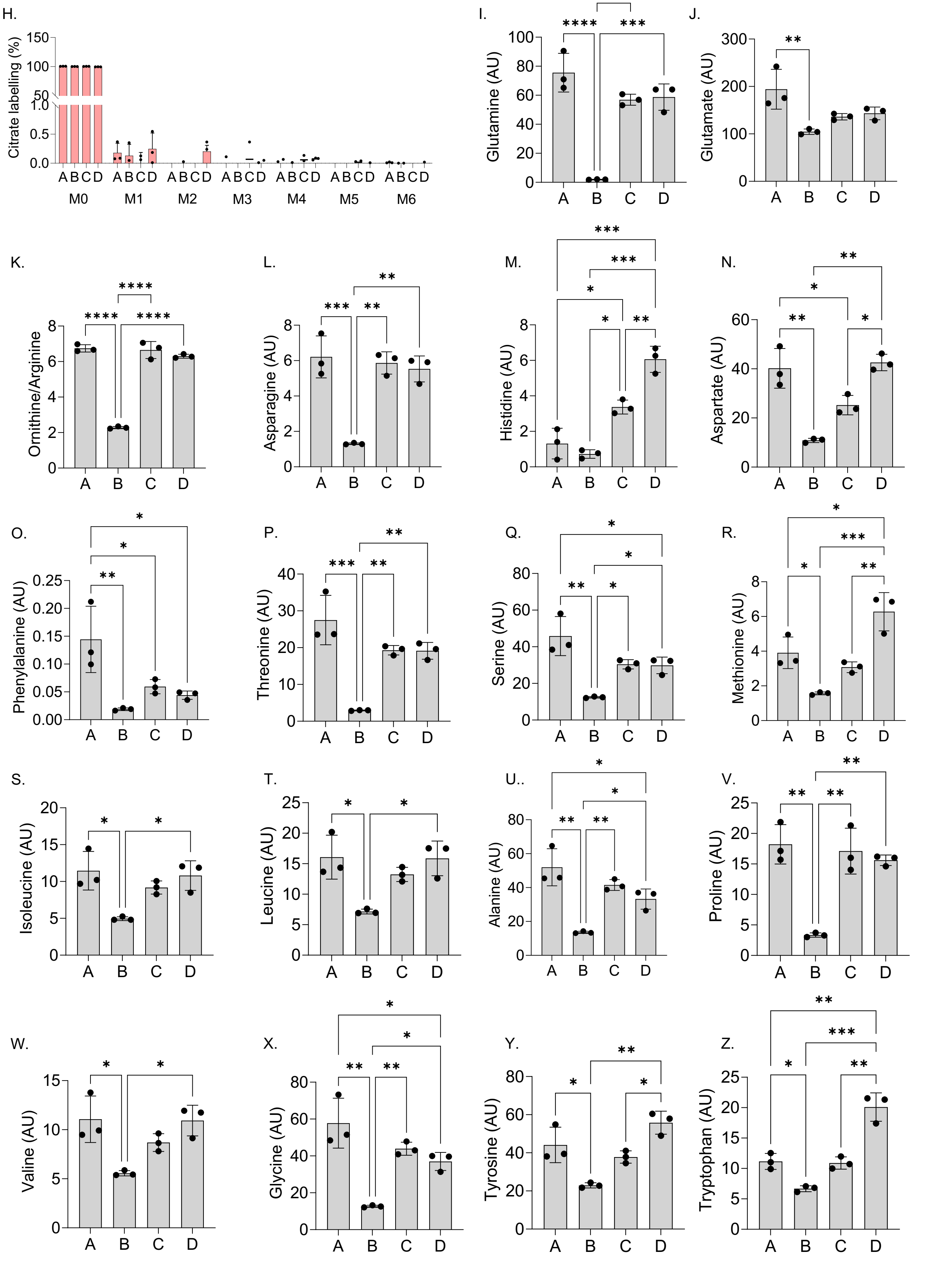

Supplementary Figure 9: Additional evidence of APOL1 G1's impact on regulators of energy metabolism (AMPK) and protein synthesis.

Ingenuity pathway analysis of RNAseq data from T-REx-293-G1 cells treated or not with Tet (50ng/ml) with or without VX-147 predicts (A) that APOL1 G1 impacts amino acid transport, and (B) that APOL1 G1 activates AMPK, inhibit MTOR, augment autophagy and inhibit protein synthesis—all predicted to be reversed by VX-147. (C) T-REx-293-G1 cells were treated with Tet with or without plus Dorsomorphin (5μM/10μM) or Dorsomorphin only (5μM/10μM). After 24 hours of treatment cellular cytotoxicity and viability were measured using MultiTox-Fluor Multiplex Cytotoxicity Assay kit (Promega, G9201) and CellTiter-Glo® 2.0 Assay kit (Promega, G9241). Ratio of cytotoxicity and viability was used to represent actual toxicity to cells from each treatment condition. (D)Western blot analyses shows that Co-treatment with Dorsomorphin also reduced pAMPK (pP38and LC3A-II) caused by APOL1 G1.(E) . Puromycin incorporation assay shows that Dorsomorphin rescued protein synthesis blocked by APOL1 G1. Rate of protein synthesis was measured by detecting puromycin-labelled nascent proteins in western blot [anti-puromycin antibody (MABE343; Sigma)]. No treatment serves as control. Densitometric quantification of the western blot protein data is shown by Bar-diagram. (F) T-REx-293 G1 cells were treated with Tet (50ng/ml) for 8 hours. Intracellular (from cell lysate; Targeted metabolomics) and extracellular (from media; Un-targeted metabolomics) levels of glutamic acid, beta-amino-isobutyric acid and gamma-amino-n-butyric acid from the same experiment were compared. (G). Western blot analyses showing the level of phospho-GCN2 (T899), total GCN2, phospho-PKR (T451), total PKR, phospho-PERK (T980) and total PERK in T-REx-293 G1 cells after 8 hours of treatment with Tetracycline (50ng/ml) only or Tetracycline (50ng/ml) + VX-147(3μM). No treatment serves as control. (H-Z) TCA cycle intermediate labeling derived from ^{13}C lysine and the changes of amino acids under treatments of Tetracycline and VX-147. (H) The labeling of citrate isotopomers derived from ^{13}C lysine. M1-M6 mean one or more ^{13}C labeled citrate.

I-Z. Amino acid levels under the treatments of Tetracycline and VX-147.

A: Control (i.e., no treatment but labeled 1hr with L-Lysine $^{13}C_6$,$^{15}N_2$).

B: Tet (50ng/ml) treatment for 8 hours and then labeled 1hr with L-Lysine $^{13}C_6$,$^{15}N_2$.

C: Tet (50ng/ml) plus VX-147 (3uM) for 8 hours and then labeled 1hour with L-Lysine $^{13}C_6$,$^{15}N_2$, in presence of 3μM VX-147.

D: Tet (50ng/ml) for 8 hours and then labeled 1 hour with L-Lysine $^{13}C_6$,$^{15}N_2$, in presence of 3μM VX-147.

All data are presented as mean ± SD. GraphPad Prism 9 software was used for data analysis. ****pValue≤0.0001, ***pValue≤0.001, **pValue≤0.005, *pValue≤0.05. One-way ANOVA with Tukey's multiple-comparison test

Supplementary Figure 10

A.

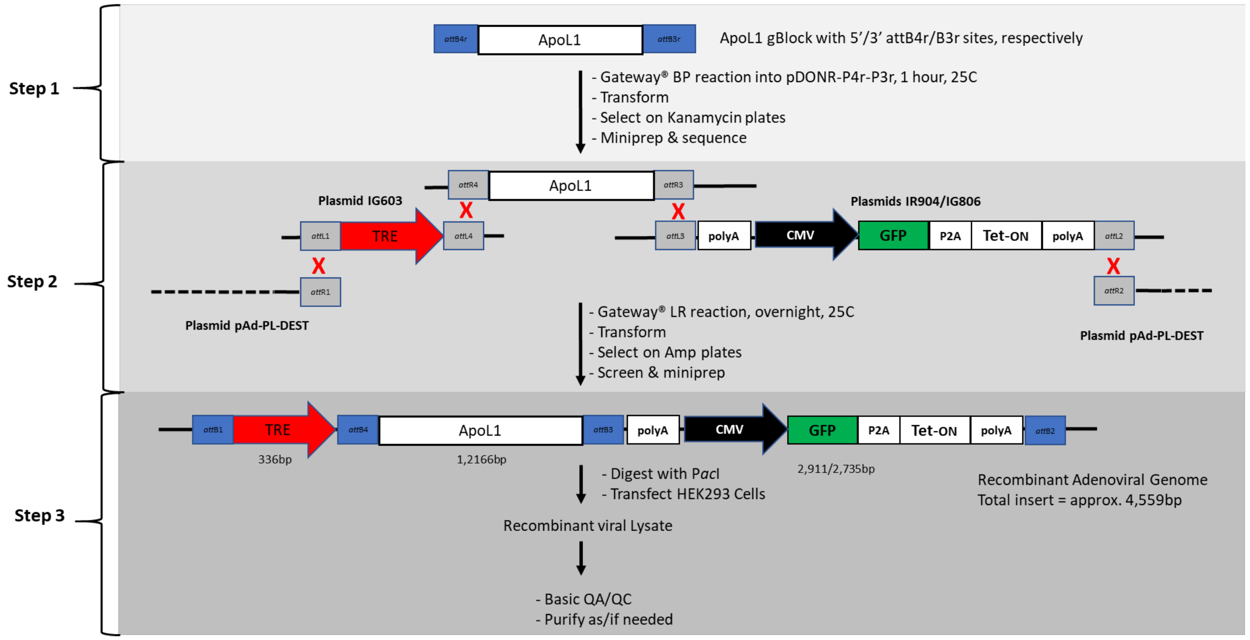

B.

APOL1-G0-coding sequence (K150)

ATGGAGGGAGCTGCTTTGCTGAGAGTCTCTGTCCTCTGCATCTGGATGAGTGCACTTTTCCTTGGTGTGGGAGTGAGGGCAGAGGAAGCTGGAGCGAGGGTGCAACAAAACGTTCCAAGTGGGACAGATACTGGAGATCCTCAAAG
TAAGCCCCTCGGTGACTGGGCTGCTGGCACCATGGACCCAGAGAGCAGTATCTTTATTGAGGATGCCATTAAGTATTTCAAGGAAAAAGTGAGCACACAGAATCTGCTACTCCTGCTGACTGATAATGAGGCCTGGAACGGATTCG
TGGCTGCTGCTGAACTGCCCAGGAATGAGGCAGATGAGCTCCGTAAAGCTCTGGACAACCTTGCAAGACAAATGATCATGAAAGACAAAAACTGGCACGATAAAGGCCAGCAGTACAGAAACTGGTTTCTGAAAGAGTTTCCTCGG
TTGAAAAGTAAGCTTGAGGATAACATAAGAAGGCTCCGTGCCCTTGCAGATGGGGTTCAGAAGGTCCACAAAGGCACCACCATCGCCAATGTGGTGTCTGGCTCTCTCAGCATTTCCTCTGGCATCCTGACCCTCGTCGGCATGGG
TCTGGCACCCTTCACAGAGGGAGGCAGCCTTGTACTCTTGGAACCTGGGATGGAGTTGGGAATCACAGCAGCTTTGACCGGGATTACCAGCAGTACCATAGACTACGGAAAGAAGTGGTGGACACAAGCCCAAGCCCACGACCTGG
TCATCAAAAGCCTTGACAAATTGAAGGAGGTGAAGGAGTTTTTGGGTGAGAACATATCCAACTTTCTTTCCTTAGCTGGCAATACTTACCAACTCACACGAGGCATTGGGAAGGACATCCGTGCCCTCAGACGAGCCAGAGCCAAT
CTTCAGTCAGTACCGCATGCCTCAGCCTCACGCCCCCGGGTCACTGAGCCAATCTCAGCTGAAAGCGGTGAACAGGTGGAGAGAGTTAATGAACCCAGCATCCTGGAAATGAGCAGAGGAGTCAAGCTCACGGATGTGGCCCCTGT
AAGCTTCTTTCTTGTGCTGGATGTAGTCTACCTCGTGTACGAATCAAAGCACTTACATGAGGGGGCAAAGTCAGAGACAGCTGAGGAGCTGAAGAAGGTGGCTCAGGAGCTGGAGGAGAAGCTAAACATTCTCAACAAT**AATTAT**A
AGATTCTGCAGGCGGACCAAGAACTGTGATCA

APOL1-G1-E150-coding sequence

ATGGAGGGAGCTGCTTTGCTGAGAGTCTCTGTCCTCTGCATCTGGATGAGTGCACTTTTCCTTGGTGTGGGAGTGAGGGCAGAGGAAGCTGGAGCGAGGGTGCAACAAAACGTTCCAAGTGGGACAGATACTGGAGATCCTCAAAG
TAAGCCCCTCGGTGACTGGGCTGCTGGCACCATGGACCCAGAGAGCAGTATCTTTATTGAGGATGCCATTAAGTATTTCAAGGAAAAAGTGAGCACACAGAATCTGCTACTCCTGCTGACTGATAATGAGGCCTGGAACGGATTCG
TGGCTGCTGCTGAACTGCCCAGGAATGAGGCAGATGAGCTCCGTAAAGCTCTGGACAACCTTGCAAGACAAATGATCATGAAAGACAAAAACTGGCACGATAAAGGCCAGCAGTACAGAAACTGGTTTCTGAAAGAGTTTCCTCGG
TTGAAAAGTGAGCTTGAGGATAACATAAGAAGGCTCCGTGCCCTTGCAGATGGGGTTCAGAAGGTCCACAAAGGCACCACCATCGCCAATGTGGTGTCTGGCTCTCTCAGCATTTCCTCTGGCATCCTGACCCTCGTCGGCATGGG
TCTGGCACCCTTCACAGAGGGAGGCAGCCTTGTACTCTTGGAACCTGGGATGGAGTTGGGAATCACAGCAGCTTTGACCGGGATTACCAGCAGTACCATAGACTACGGAAAGAAGTGGTGGACACAAGCCCAAGCCCACGACCTGG
TCATCAAAAGCCTTGACAAATTGAAGGAGGTGAAGGAGTTTTTGGGTGAGAACATATCCAACTTTCTTTCCTTAGCTGGCAATACTTACCAACTCACACGAGGCATTGGGAAGGACATCCGTGCCCTCAGACGAGCCAGAGCCAAT
CTTCAGTCAGTACCGCATGCCTCAGCCTCACGCCCCCGGGTCACTGAGCCAATCTCAGCTGAAAGCGGTGAACAGGTGGAGAGAGTTAATGAACCCAGCATCCTGGAAATGAGCAGAGGAGTCAAGCTCACGGATGTGGCCCCTGT
A**GGC**TTCTTTCTTGTGCTGGATGTAGTCTACCTCGTGTACGAATCAAAGCACTTACATGAGGGGGCAAAGTCAGAGACAGCTGAGGAGCTGAAGAAGGTGGCTCAGGAGCTGGAGGAGAAGCTAAAC**ATG**CTCAACAATAATTATA
AGATTCTGCAGGCGGACCAAGAACTGTGATCA

NOTE:
1) IN G0, 150 = K (AAG); IN G1 AND G2 150 = E (GAG)
2) G1: S342G, I384M

C.

gBlock Design:

APOL1-G0-coding sequence (K150), 1,306bp (TB1804)

CAGAGCTGGCGAACGCGTTGAACACTTCAC**ACAACTTTTCTATACAAAGTTGTAcc**ATGGAGGGAGCTGCTTTGCTGAGAGTCTCTGTCCTCTGCATCTGGATGAGTGCACTTTTCCTTGGTGTGGGAGTGAGGGCAGAGGAAGCTGGAG
CGAGGGTGCAACAAAACGTTCCAAGTGGGACAGATACTGGAGATCCTCAAAGTAAGCCCCTCGGTGACTGGGCTGCTGGCACCATGGACCCAGAGAGCAGTATCTTTATTGAGGATGCCATTAAGTATTTCAAGGAAAAAGTGAGC
ACACAGAATCTGCTACTCCTGCTGACTGATAATGAGGCCTGGAACGGATTCGTGGCTGCTGCTGAACTGCCCAGGAATGAGGCAGATGAGCTCCGTAAAGCTCTGGACAACCTTGCAAGACAAATGATCATGAAAGACAAAAACTG
GCACGATAAAGGCCAGCAGTACAGAAACTGGTTTCTGAAAGAGTTTCCTCGGTTGAAAAGTAAGCTTGAGGATAACATAAGAAGGCTCCGTGCCCTTGCAGATGGGGTTCAGAAGGTCCACAAAGGCACCACCATCGCCAATGTGG
TGTCTGGCTCTCTCAGCATTTCCTCTGGCATCCTGACCCTCGTCGGCATGGGTCTGGCACCCTTCACAGAGGGAGGCAGCCTTGTACTCTTGGAACCTGGGATGGAGTTGGGAATCACAGCAGCTTTGACCGGGATTACCAGCAGT
ACCATAGACTACGGAAAGAAGTGGTGGACACAAGCCCAAGCCCACGACCTGGTCATCAAAAGCCTTGACAAATTGAAGGAGGTGAAGGAGTTTTTGGGTGAGAACATATCCAACTTTCTTTCCTTAGCTGGCAATACTTACCAACT
CACACGAGGCATTGGGAAGGACATCCGTGCCCTCAGACGAGCCAGAGCCAATCTTCAGTCAGTACCGCATGCCTCAGCCTCACGCCCCCGGGTCACTGAGCCAATCTCAGCTGAAAGCGGTGAACAGGTGGAGAGAGTTAATGAAC
CCAGCATCCTGGAAATGAGCAGAGGAGTCAAGCTCACGGATGTGGCCCCTGTAAGCTTCTTTCTTGTGCTGGATGTAGTCTACCTCGTGTACGAATCAAAGCACTTACATGAGGGGGCAAAGTCAGAGACAGCTGAGGAGCTGAAG
AAGGTGGCTCAGGAGCTGGAGGAGAAGCTAAACATTCTCAACAAT**AATTAT**AAGATTCTGCAGGCGGACCAAGAACTGTGA**ACAACTTTGTATAATAAAGTTGT**CCTGAATAACGTACTCATCTCAACTCATTC **576bp**

APOL1-G1-E150-coding sequence 1,306bp (TB1805)

CAGAGCTGGCGAACGCGTTGAACACTTCAC**ACAACTTTTCTATACAAAGTTGTAcc**ATGGAGGGAGCTGCTTTGCTGAGAGTCTCTGTCCTCTGCATCTGGATGAGTGCACTTTTCCTTGGTGTGGGAGTGAGGGCAGAGGAAGCTGGAG
CGAGGGTGCAACAAAACGTTCCAAGTGGGACAGATACTGGAGATCCTCAAAGTAAGCCCCTCGGTGACTGGGCTGCTGGCACCATGGACCCAGAGAGCAGTATCTTTATTGAGGATGCCATTAAGTATTTCAAGGAAAAAGTGAGC
ACACAGAATCTGCTACTCCTGCTGACTGATAATGAGGCCTGGAACGGATTCGTGGCTGCTGCTGAACTGCCCAGGAATGAGGCAGATGAGCTCCGTAAAGCTCTGGACAACCTTGCAAGACAAATGATCATGAAAGACAAAAACTG
GCACGATAAAGGCCAGCAGTACAGAAACTGGTTTCTGAAAGAGTTTCCTCGGTTGAAAAGTGAGCTTGAGGATAACATAAGAAGGCTCCGTGCCCTTGCAGATGGGGTTCAGAAGGTCCACAAAGGCACCACCATCGCCAATGTGG
TGTCTGGCTCTCTCAGCATTTCCTCTGGCATCCTGACCCTCGTCGGCATGGGTCTGGCACCCTTCACAGAGGGAGGCAGCCTTGTACTCTTGGAACCTGGGATGGAGTTGGGAATCACAGCAGCTTTGACCGGGATTACCAGCAGT
ACCATAGACTACGGAAAGAAGTGGTGGACACAAGCCCAAGCCCACGACCTGGTCATCAAAAGCCTTGACAAATTGAAGGAGGTGAAGGAGTTTTTGGGTGAGAACATATCCAACTTTCTTTCCTTAGCTGGCAATACTTACCAACT
CACACGAGGCATTGGGAAGGACATCCGTGCCCTCAGACGAGCCAGAGCCAATCTTCAGTCAGTACCGCATGCCTCAGCCTCACGCCCCCGGGTCACTGAGCCAATCTCAGCTGAAAGCGGTGAACAGGTGGAGAGAGTTAATGAAC
CCAGCATCCTGGAAATGAGCAGAGGAGTCAAGCTCACGGATGTGGCCCCTGTA**GGC**TTCTTTCTTGTGCTGGATGTAGTCTACCTCGTGTACGAATCAAAGCACTTACATGAGGGGGCAAAGTCAGAGACAGCTGAGGAGCTGAAG
AAGGTGGCTCAGGAGCTGGAGGAGAAGCTAAAC**ATG**CTCAACAATAATTATAAGATTCTGCAGGCGGACCAAGAACTGTGA**ACAACTTTGTATAATAAAGTTGT**CCTGAATAACGTACTCATCTCAACTCATTC

NOTE:
1) IN G0, 150 = K (AAG); IN G1 AND G2 150 = E (GAG)
2) G1: S342G, I384M

Gray = 5'/3' random sequence
Bold = 5'/3' attB4r/B3r sequence

HindIII

Supplementary Figure 10: APOL1 G1 expression construct.

(A) Schematic illustration showing the construction and generation of recombinant adenoviral vector containing APOL1 gene. (B) Coding sequence of APOL1 G0 and G1variants used in the recombinant adenoviral construct. (C) Design of the gBlock used in the construction of the recombinant adenoviral genome.

Supplementary Figure 11

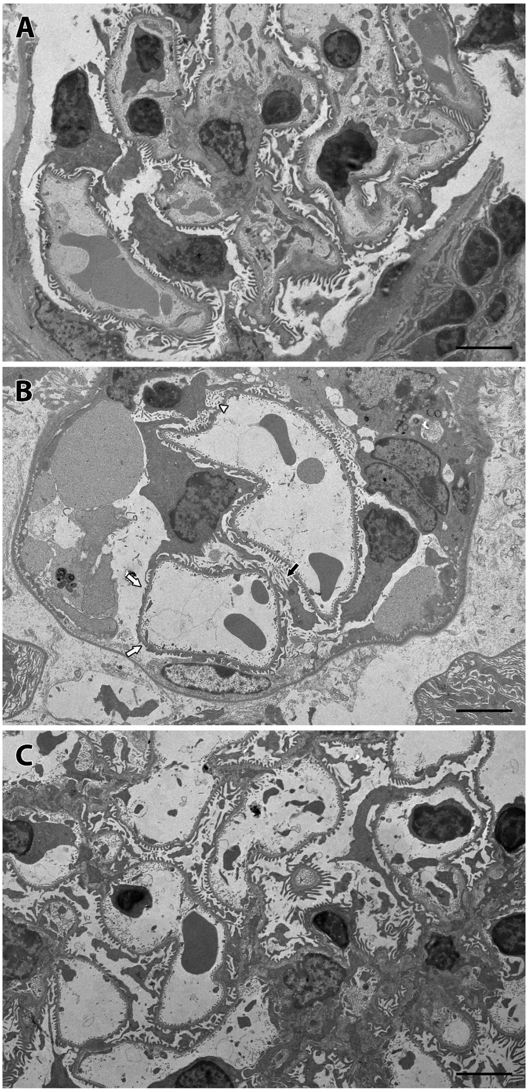

Supplementary Figure 11: Electron micrograph of APOL1 G1 transgenic mice.

Lower magnification electron micrographs of kidneys from APOL1 G1. (A) treated with PBS + vehicle, control, (B) IFNγ or (C) IFNγ + VX-147 Compared to control mice (A), FNγ-treated mice (B) have focal podocyte foot process effacement (white arrow), microvillous transformation (black arrow), and cytoplasmic shedding (white arrowhead). Treatment with VX-147 (C) rescued the cellular phenotypes. The order here is the same as in Figure 10. Bar = 400 nm.

Supplementary Figure 12.

Mouse kidney-derived podocyte staining for podocyte markers

Podocin (Green) and DAPI (Blue)

Synaptopodin (Green) and DAPI (Blue)

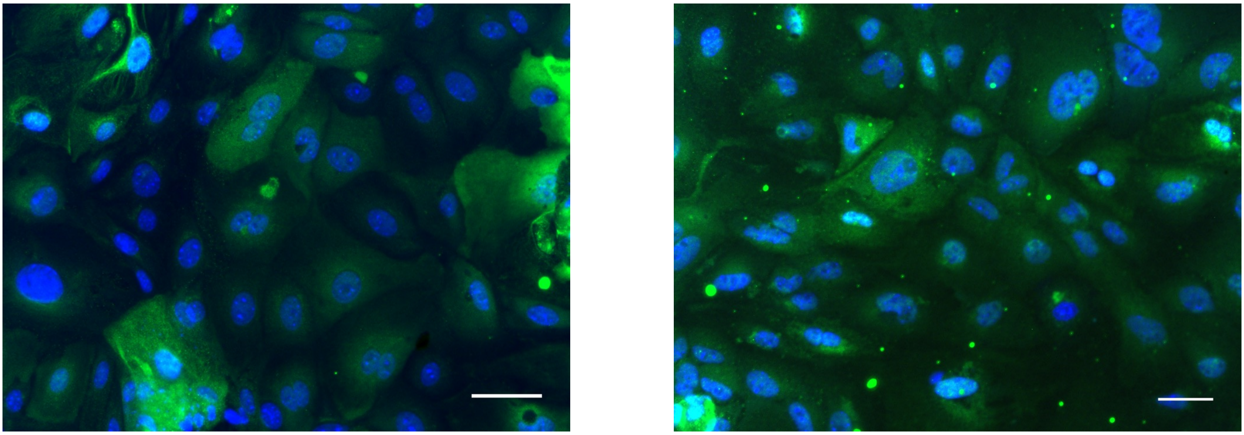

Supplementary Figure 12: Mouse kidney-derived podocytes were stained using antibodies targeting podocyte markers, podocin and synaptopodin.
