# Supplementary material for: APOL1-mediated monovalent cation transport contributes to APOL1-mediated podocytopathy in kidney disease
Source: J Clin Invest. 2024 Jan 16;134(5):e172262. doi: 10.1172/JCI172262 (PMC10904047; doi:10.1172/JCI172262)

Full unedited gel for Figure 1B.

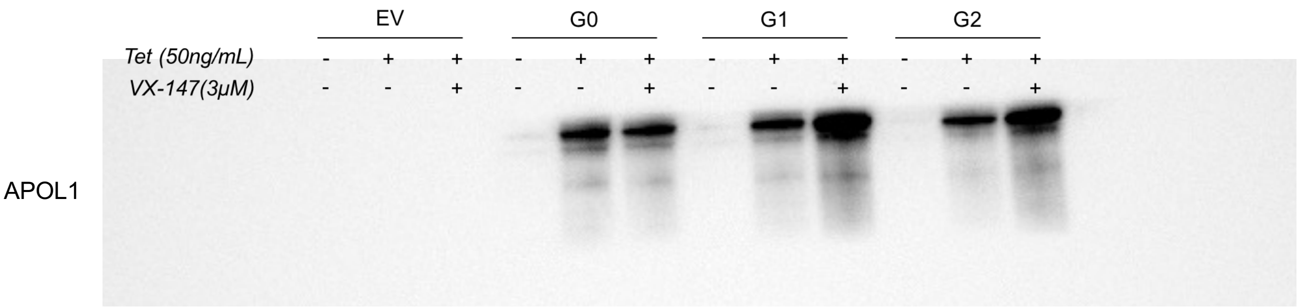

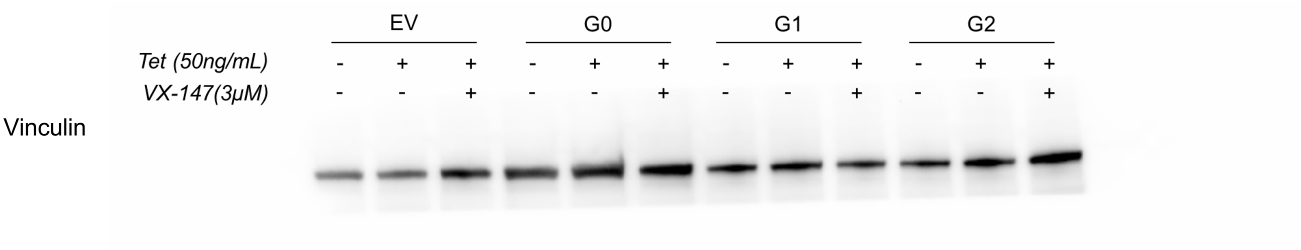

Full unedited gel for Figure 2A.

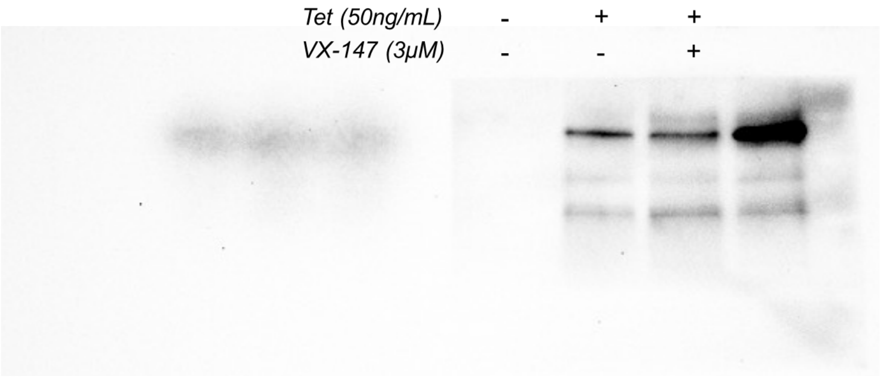

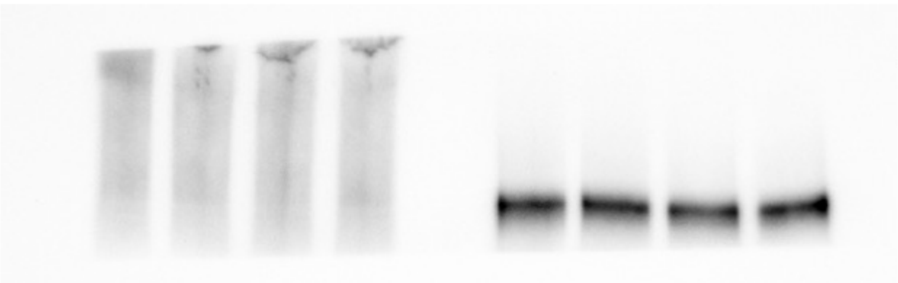

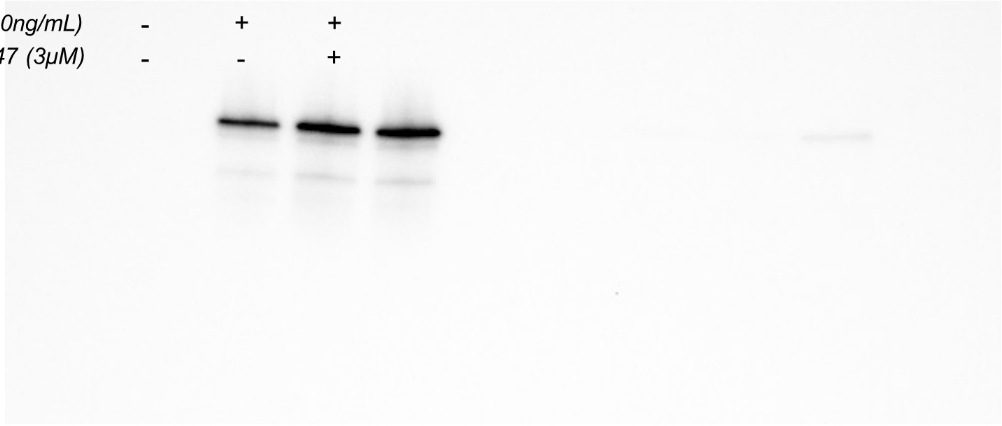

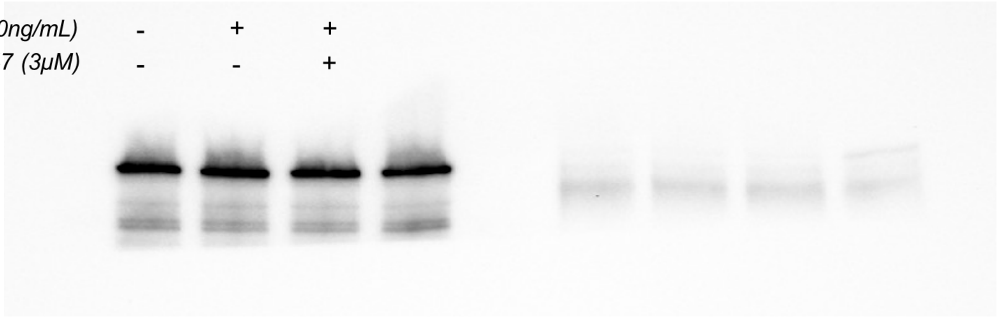

Full unedited gel for Figure 4J.

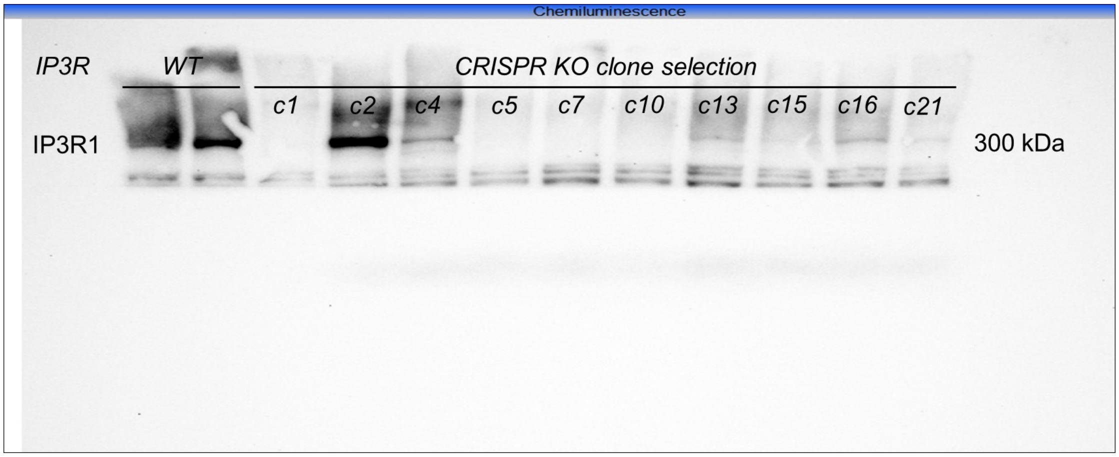

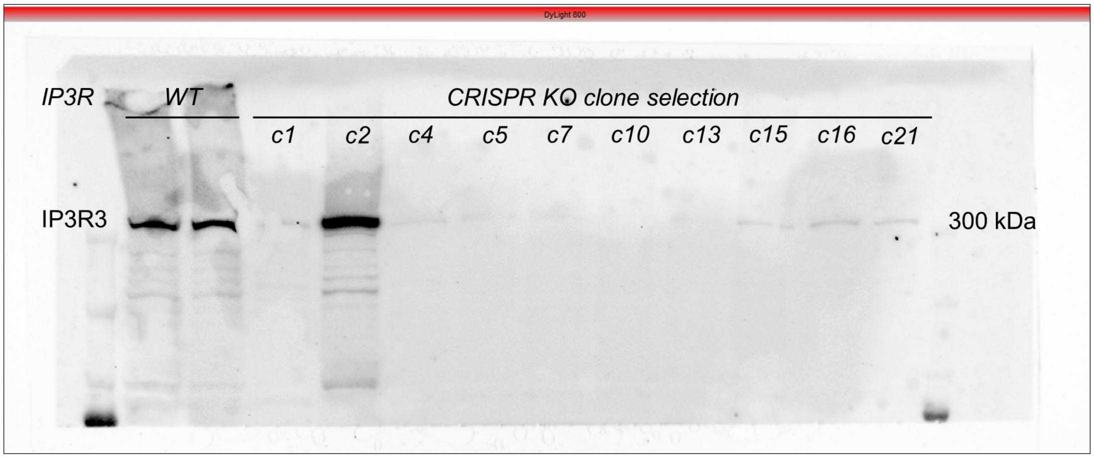

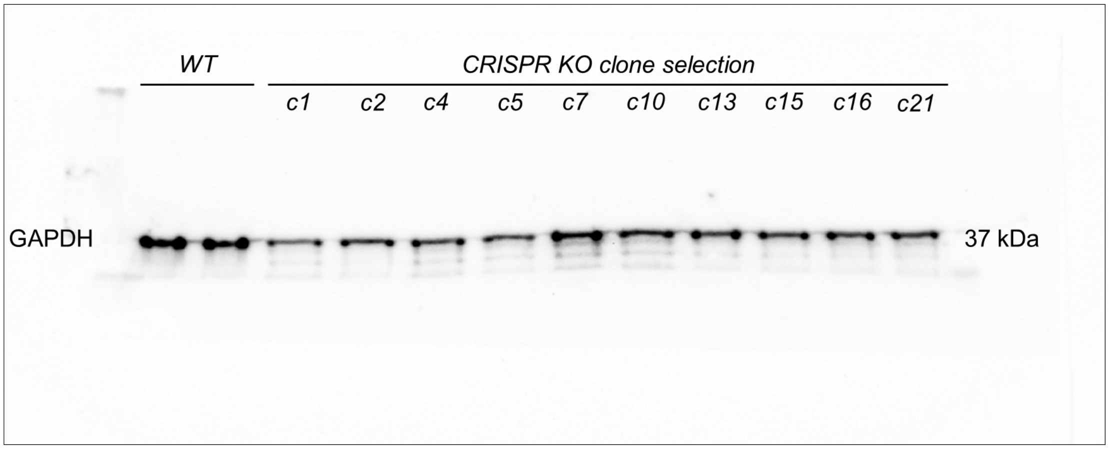

Full unedited gel for Figure 6A.

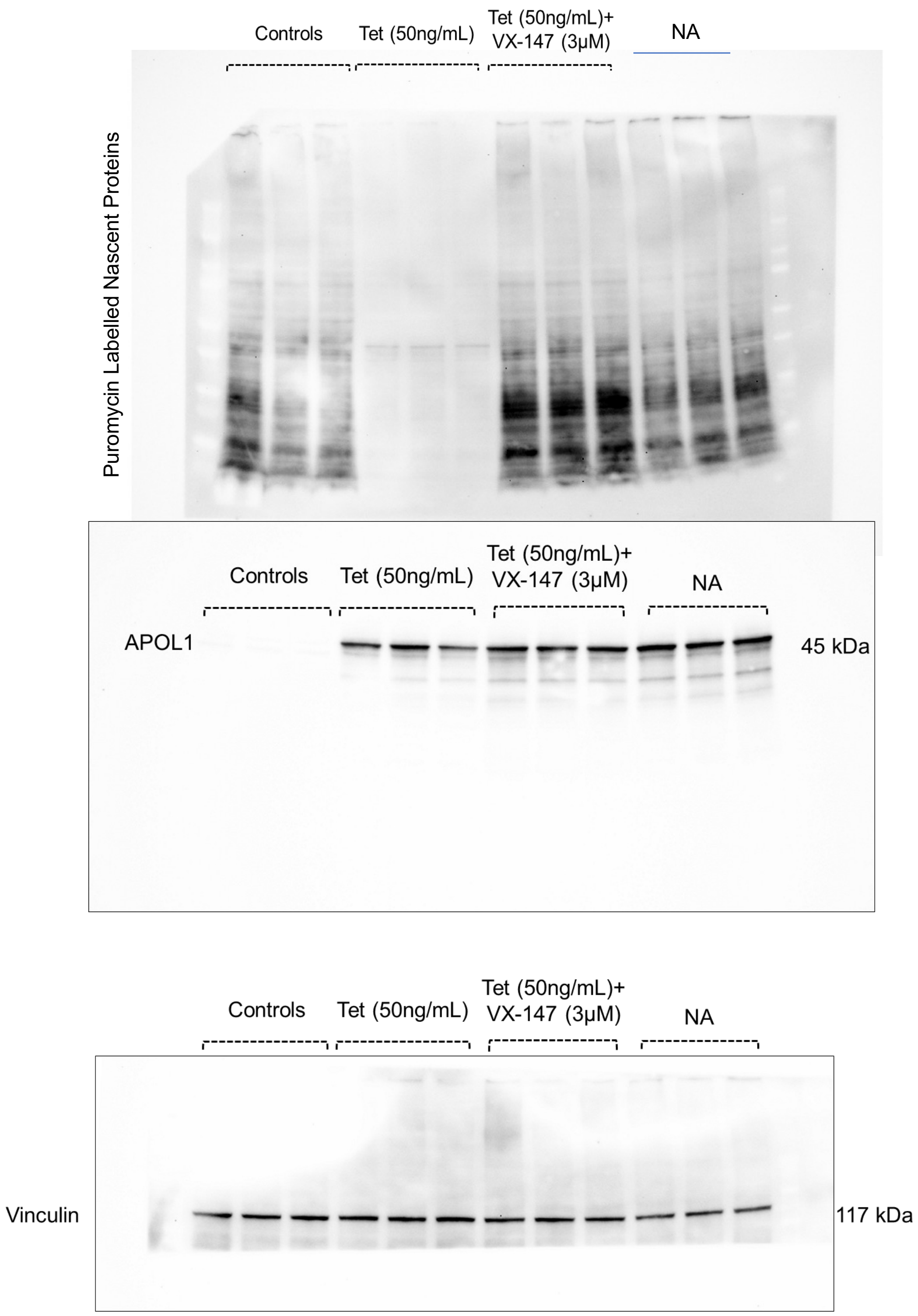

Full unedited gel for Figure 6E.

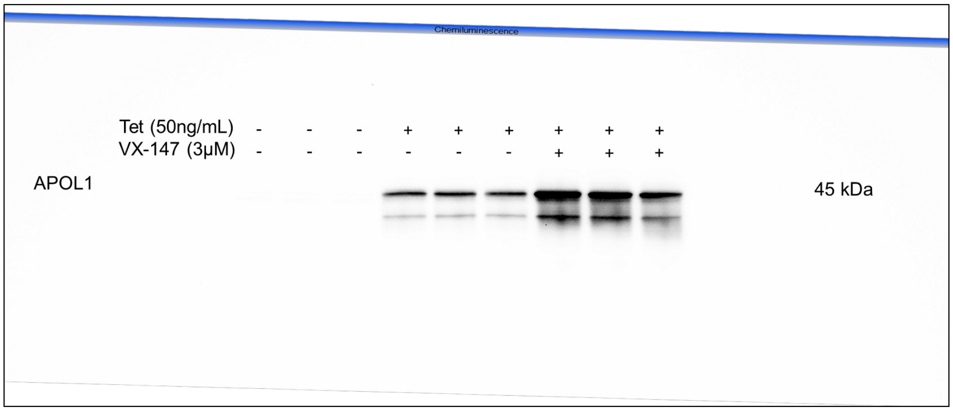

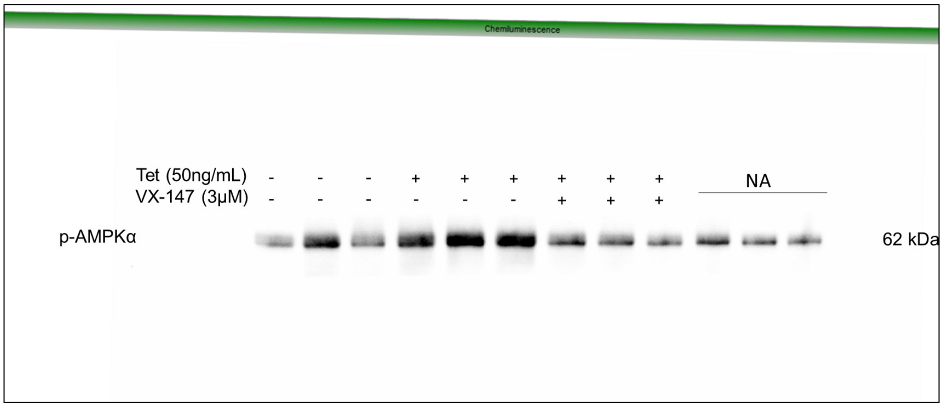

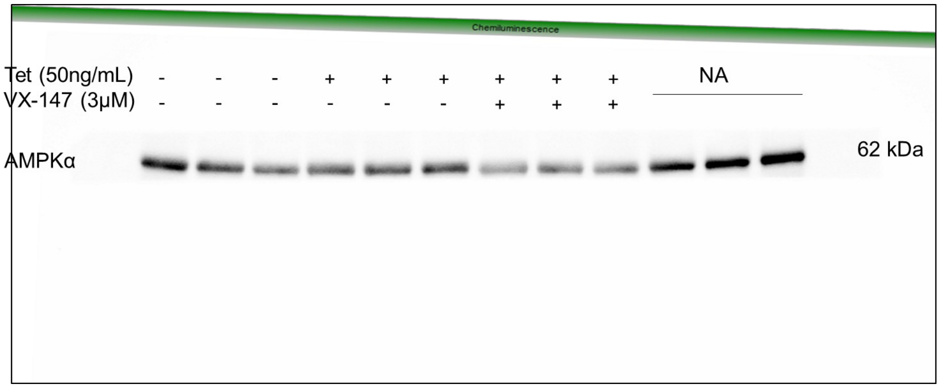

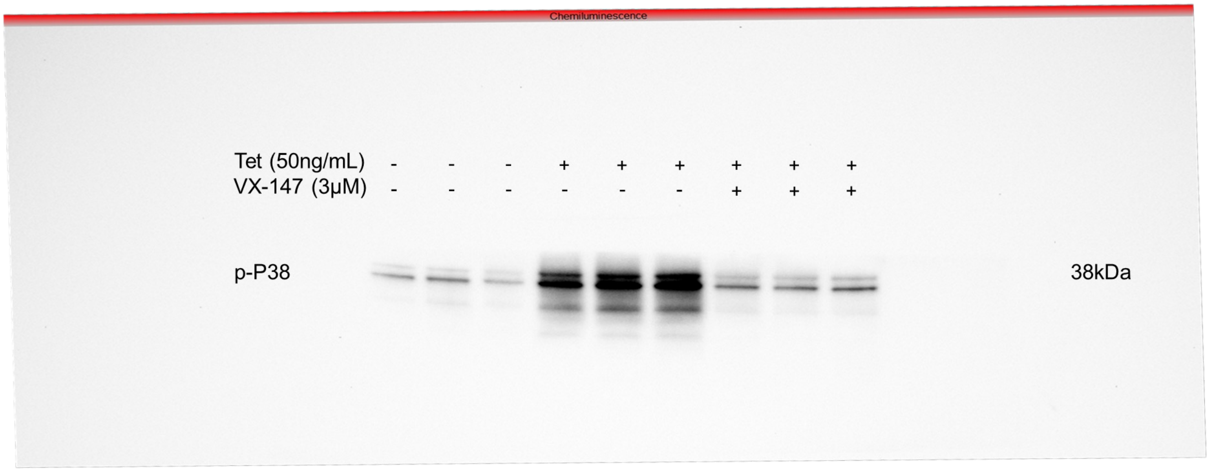

NA= Not applicable

Full unedited gel for Figure 6E.

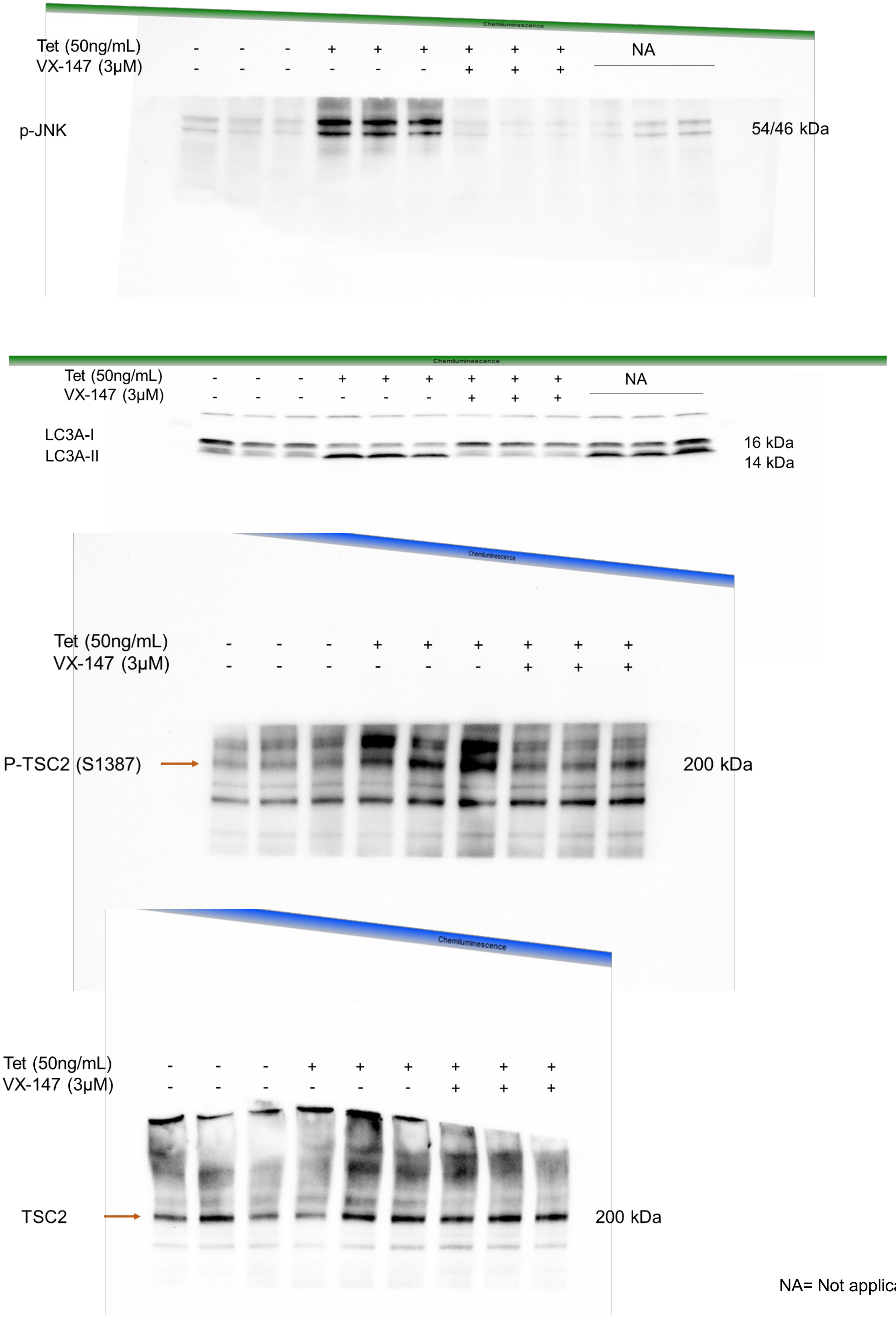

NA= Not applicable

Full unedited gel for Figure 6E.

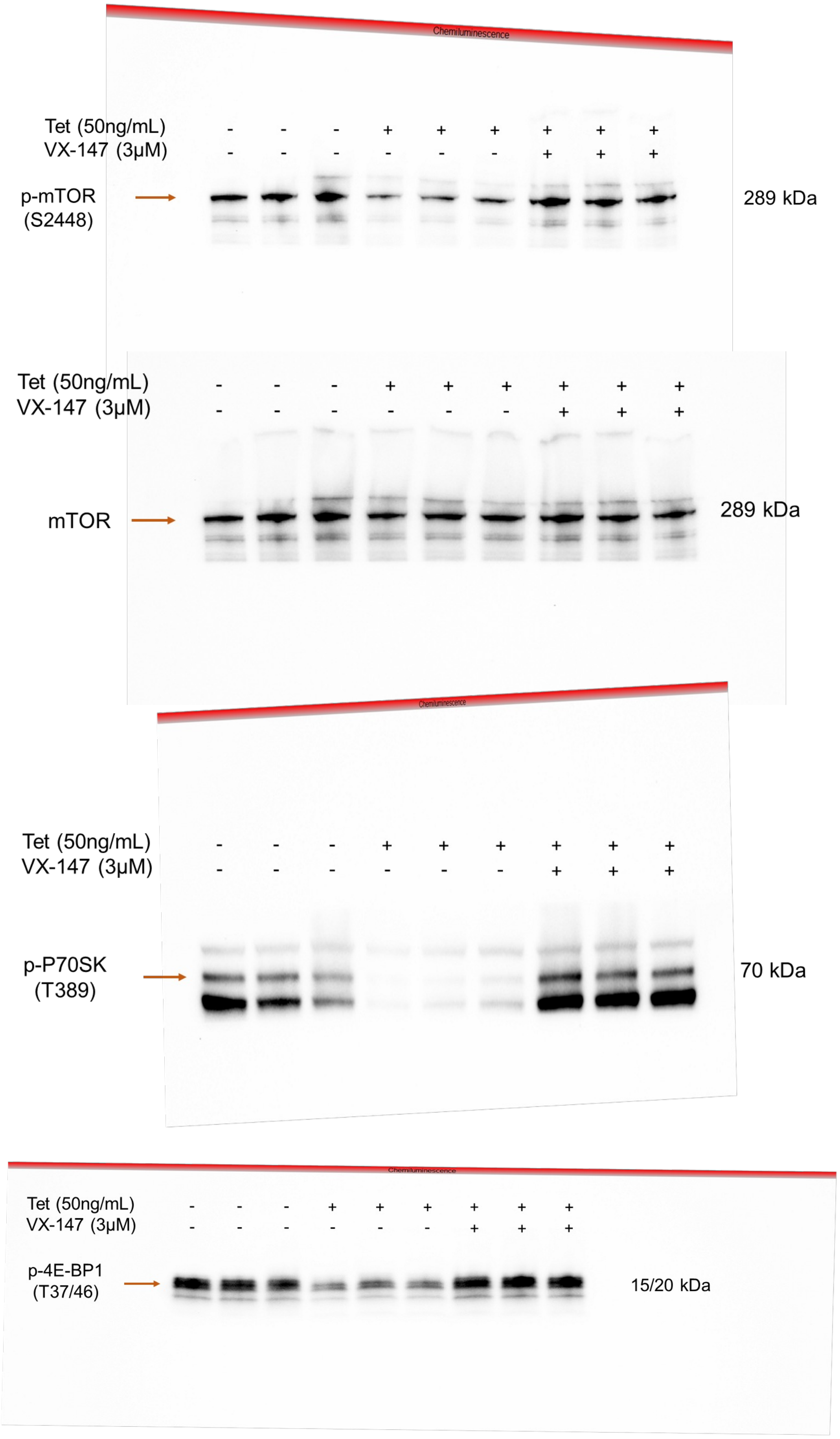

Full unedited gel for Figure 6E.

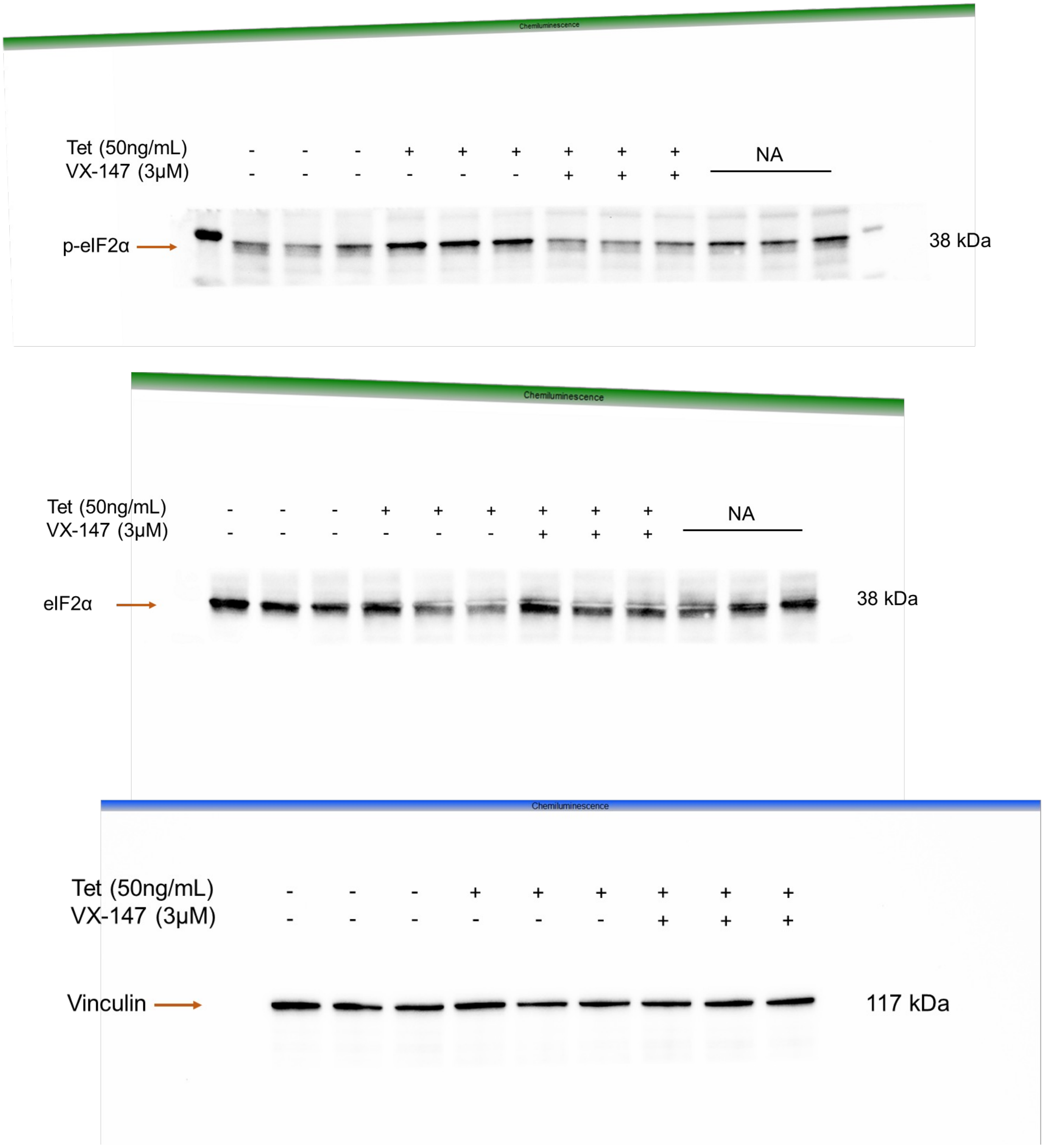

NA= Not applicable

Full unedited gel for Figure 8C.

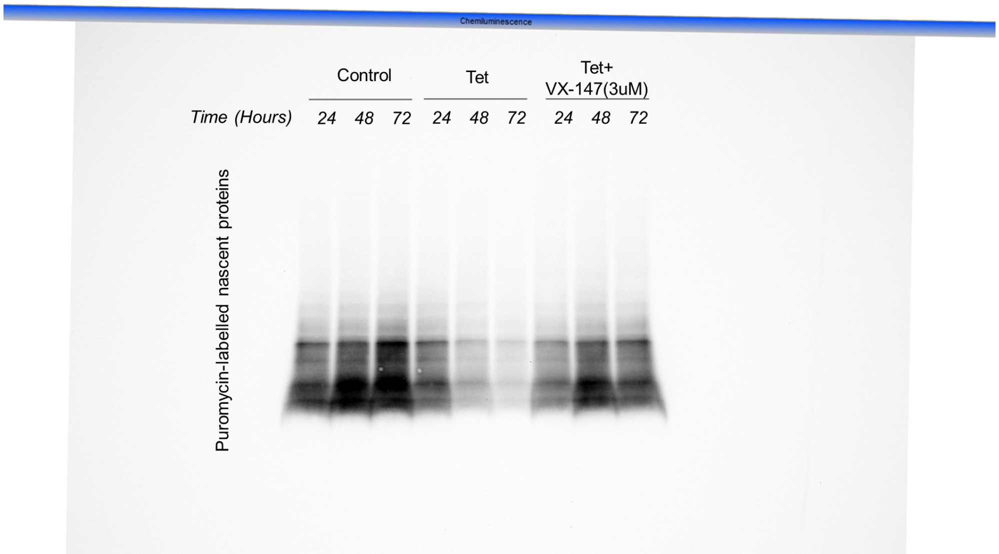

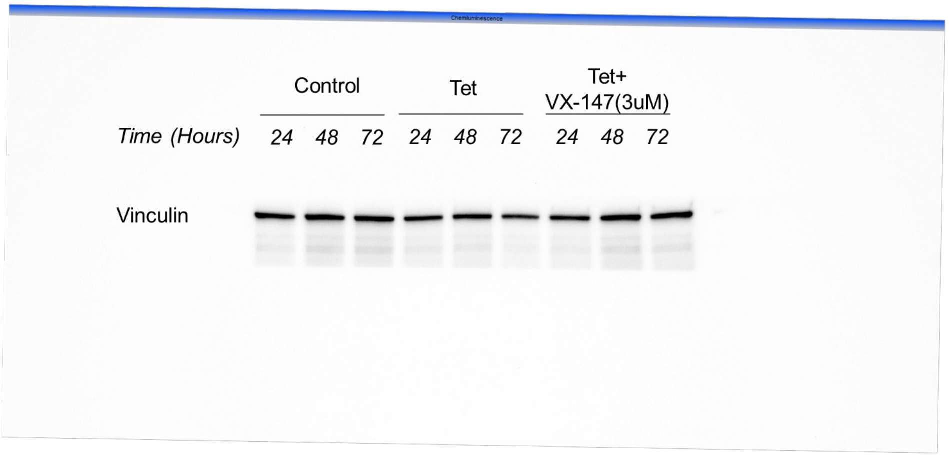

Full unedited gel for Figure 8D.

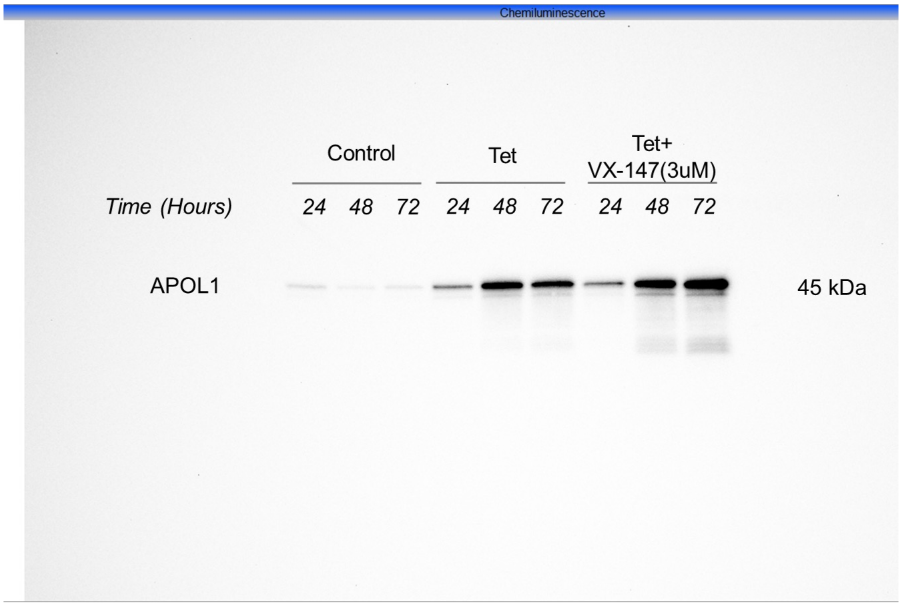

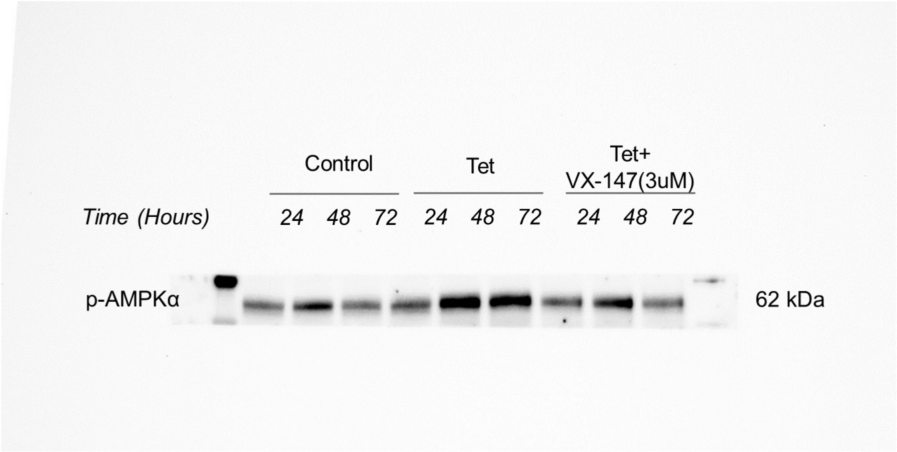

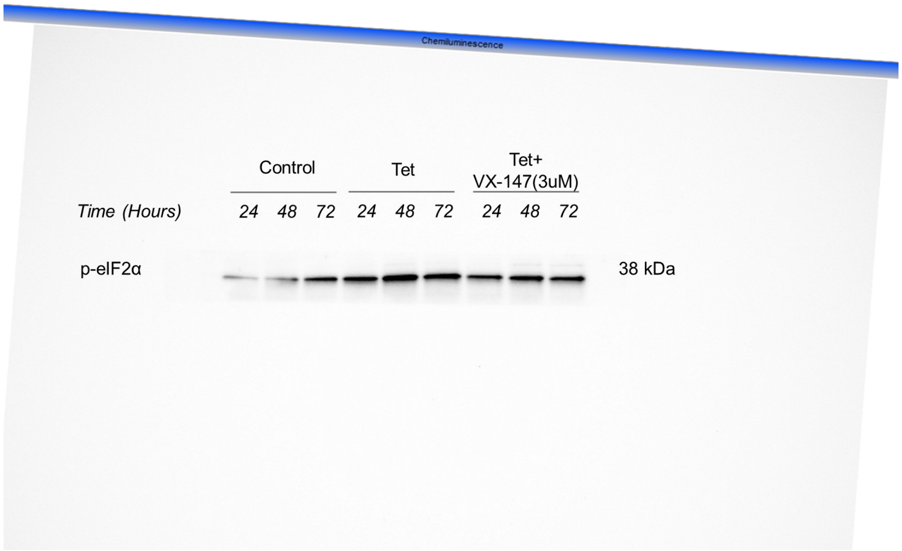

Full unedited gel for Figure 8D.

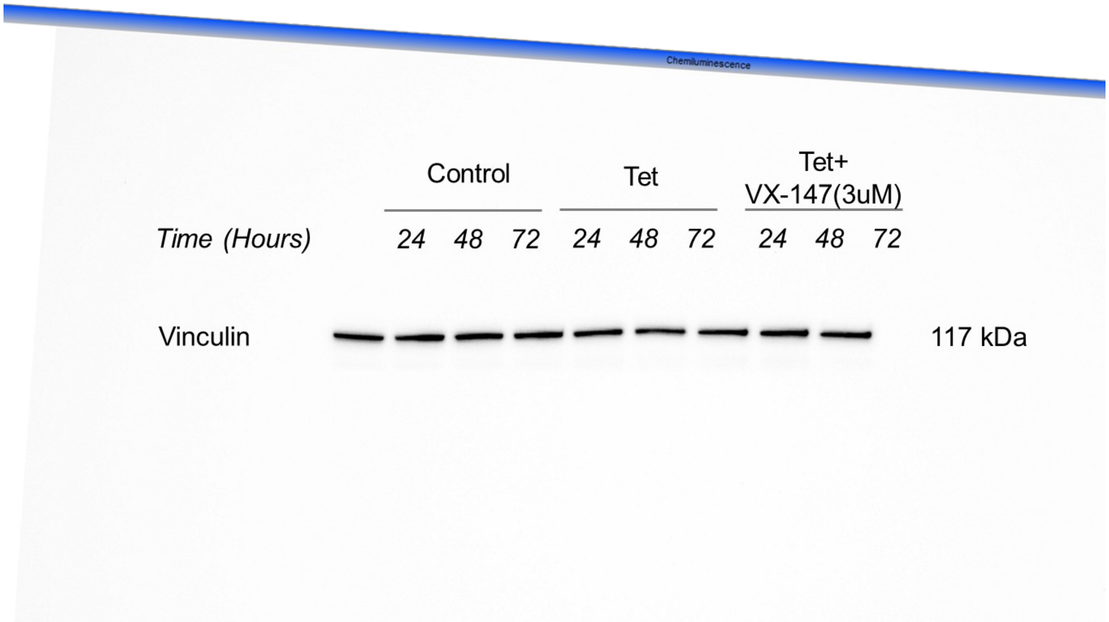

Full unedited gel for Figure 9C.

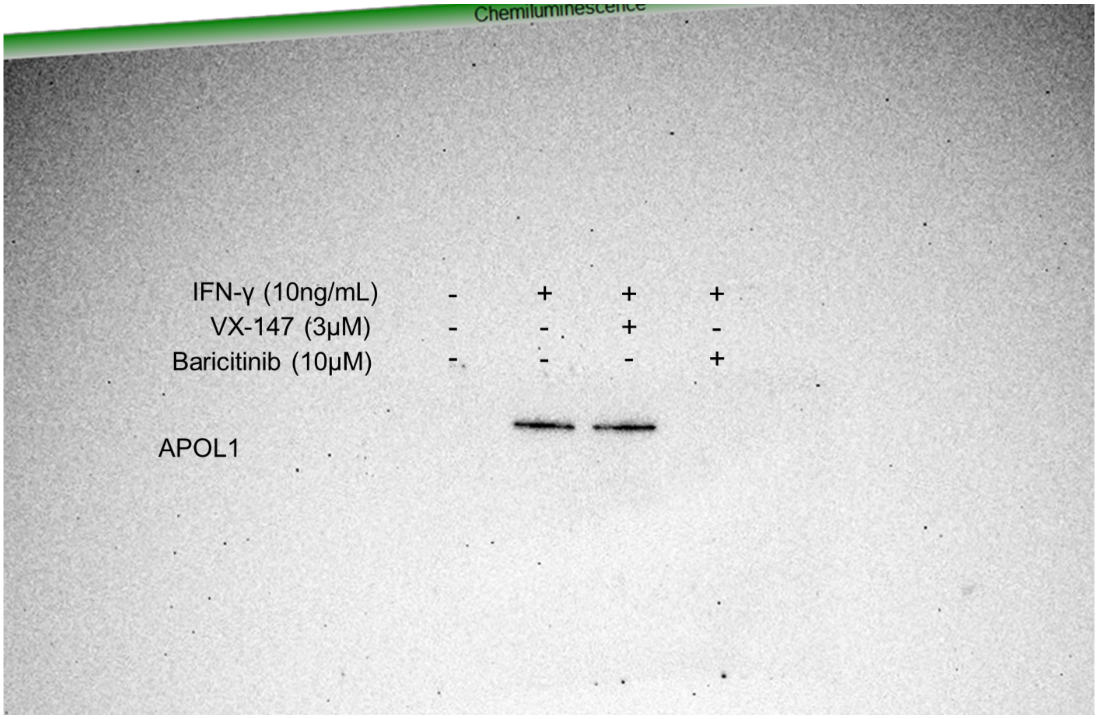

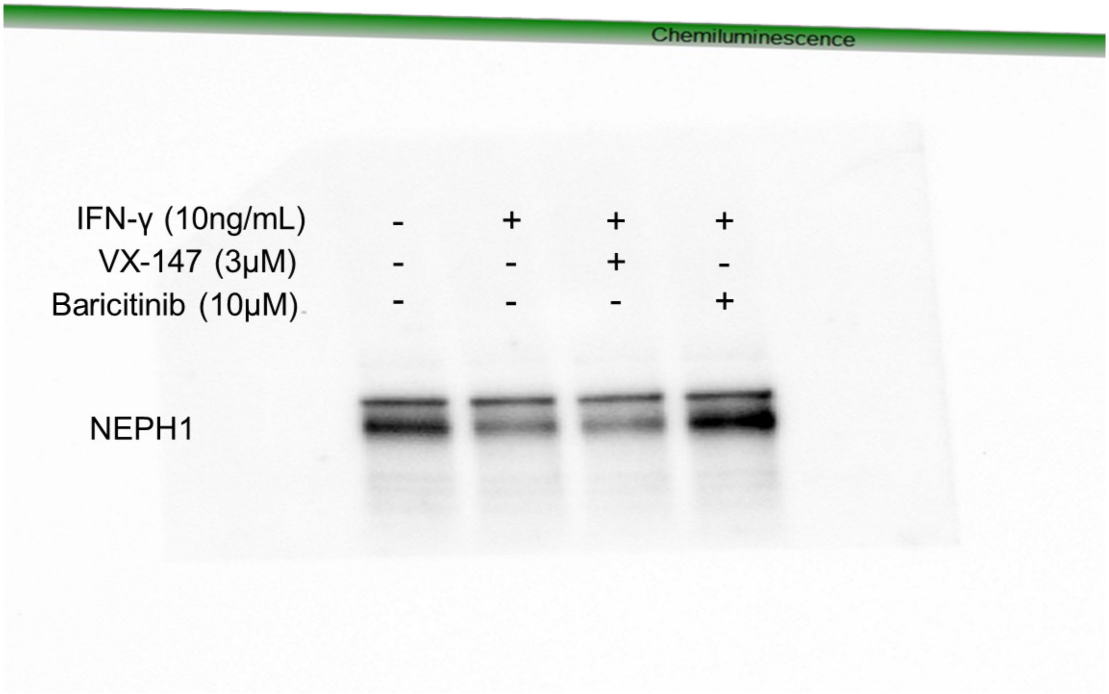

Full unedited gel for Figure 9F.

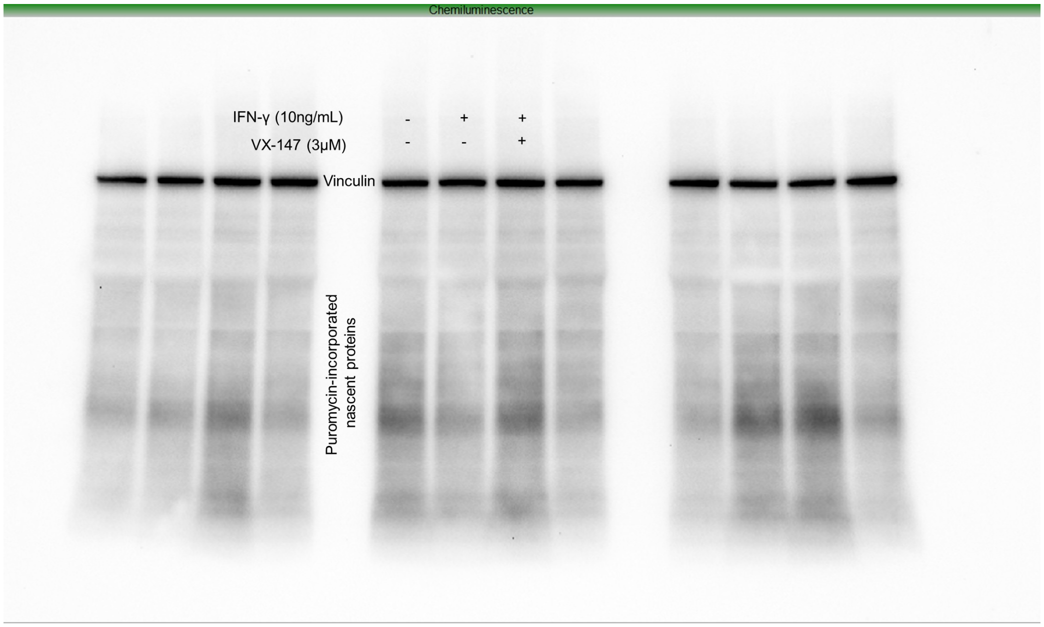

Full unedited gel for Figure 9G.

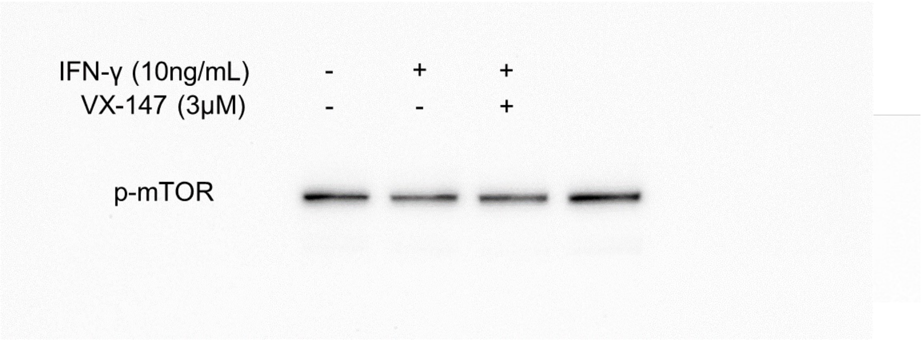

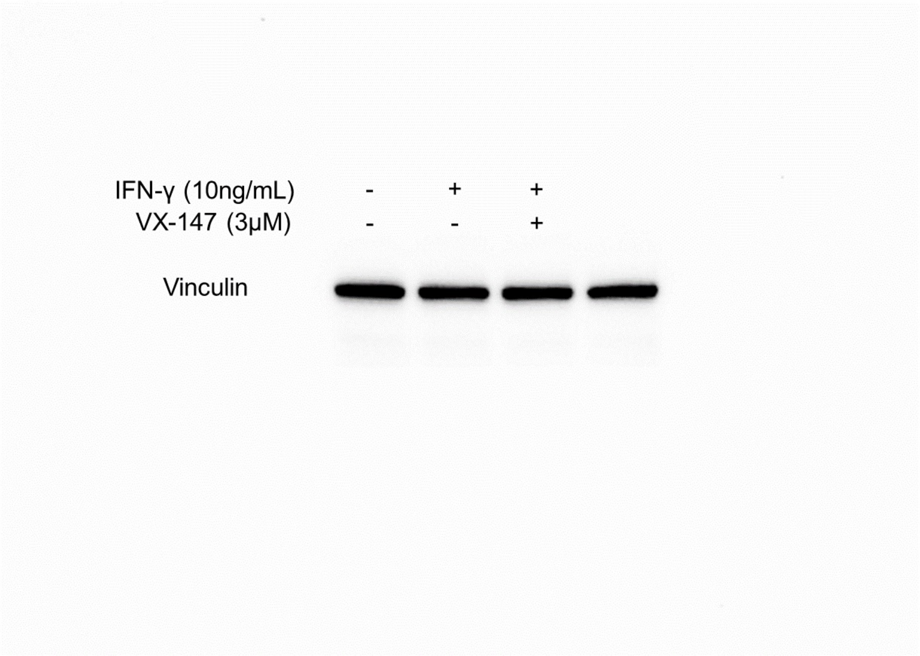

Full unedited gel for Supplementary Figure 6D.

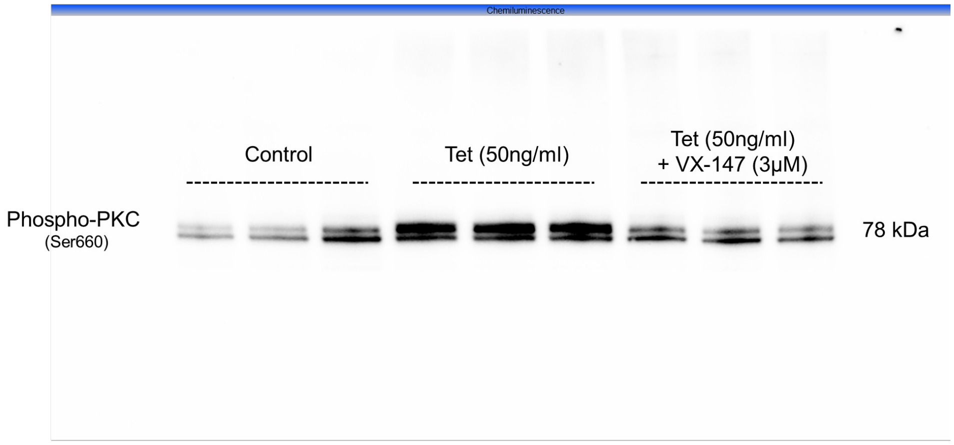

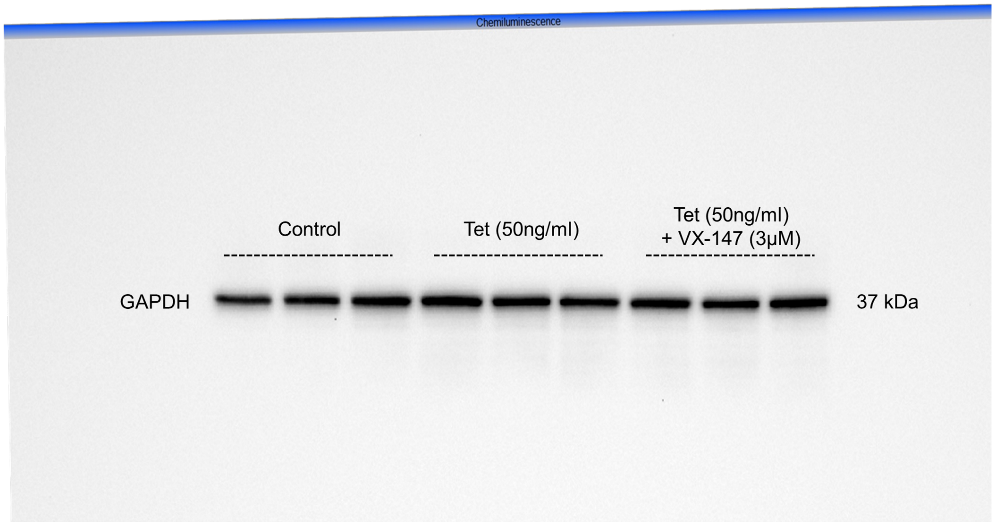

Full unedited gel for Supplementary Figure 6F.

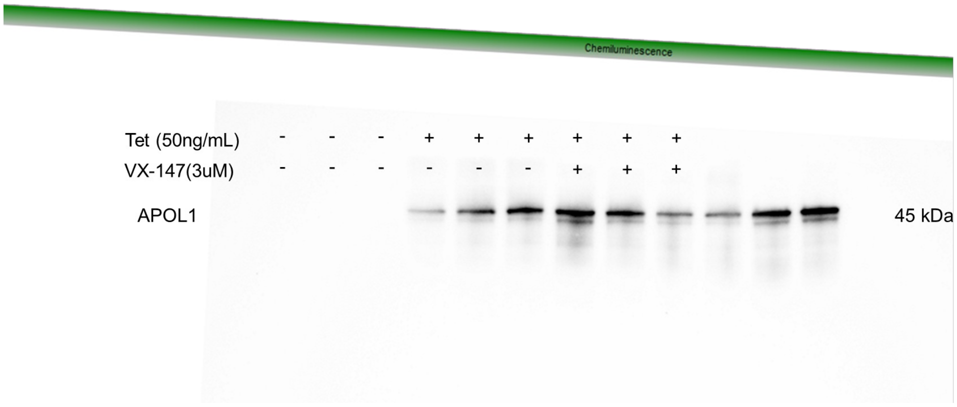

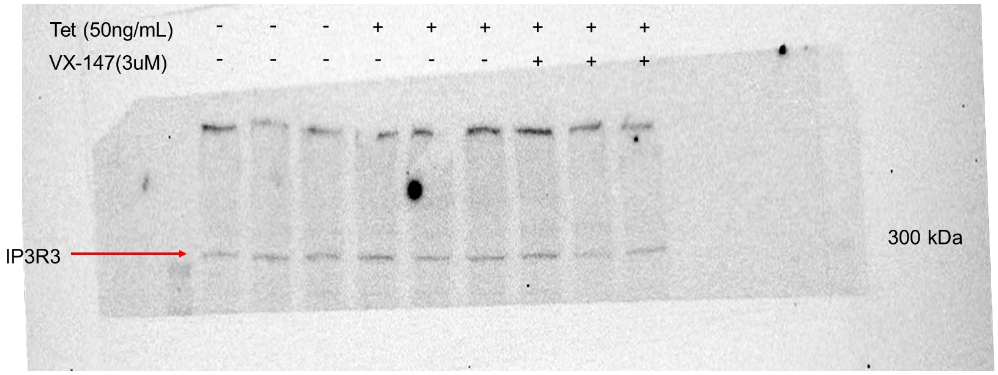

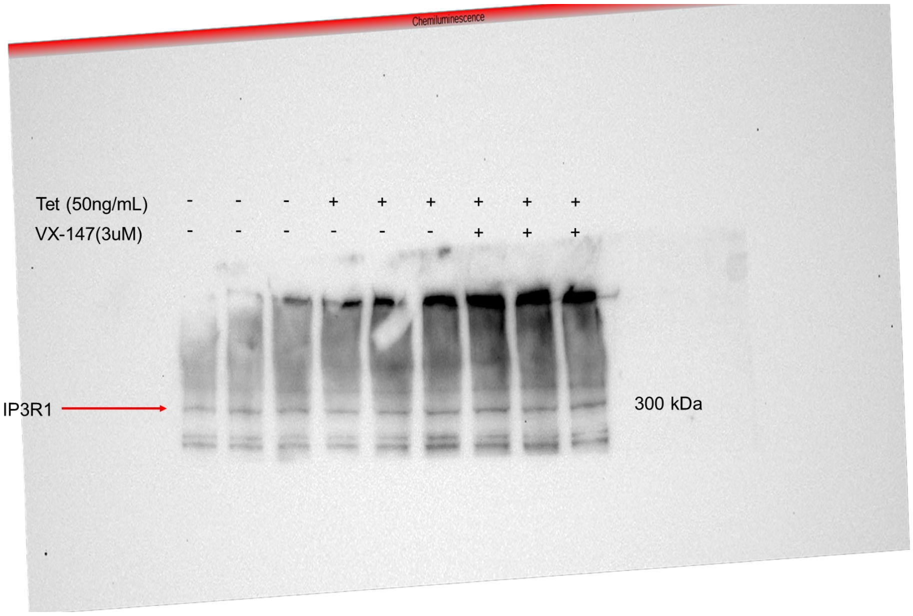

Supplementary Figure 6F.

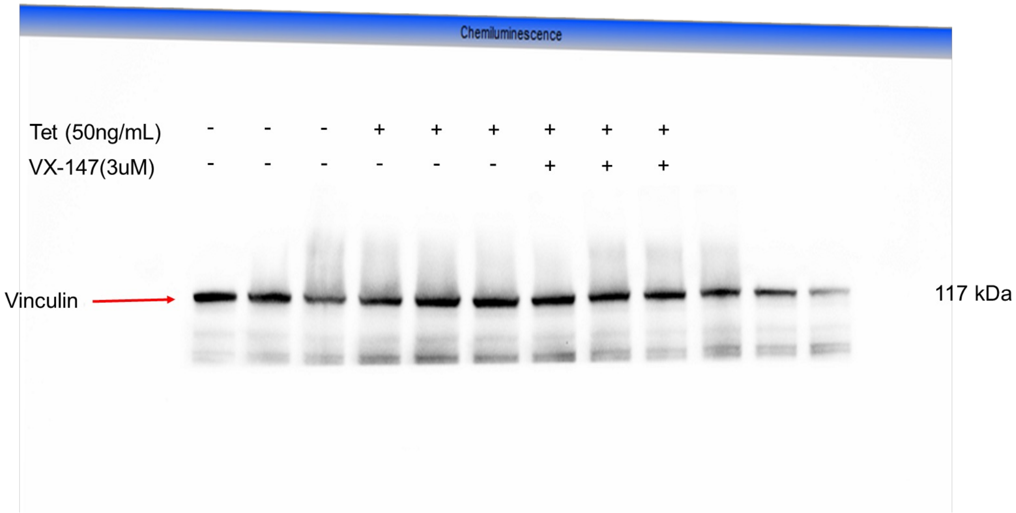

Full unedited gel for Supplementary Figure 6G.

Check Figure 4J above for the Full unedited gel

Full unedited gel for Supplementary Figure 7D.

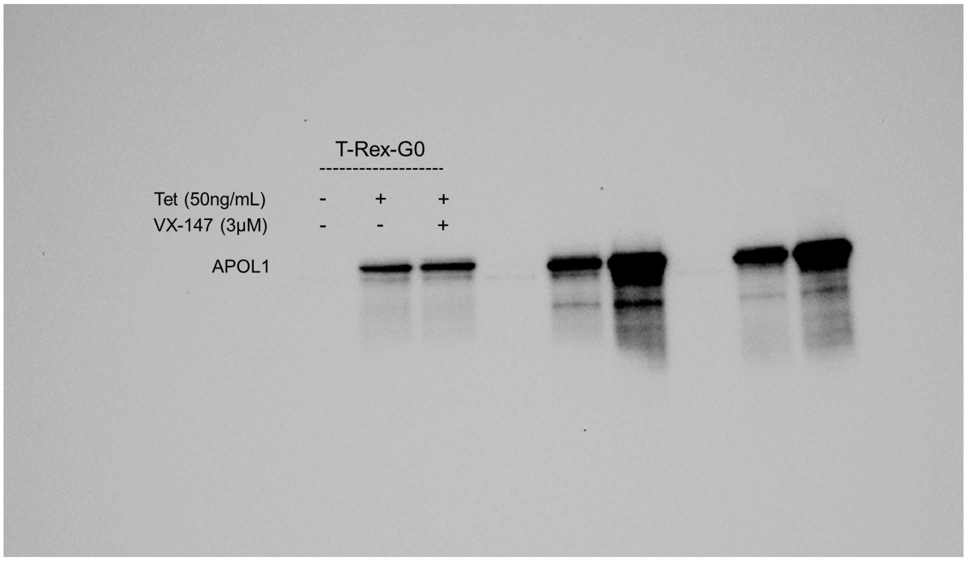

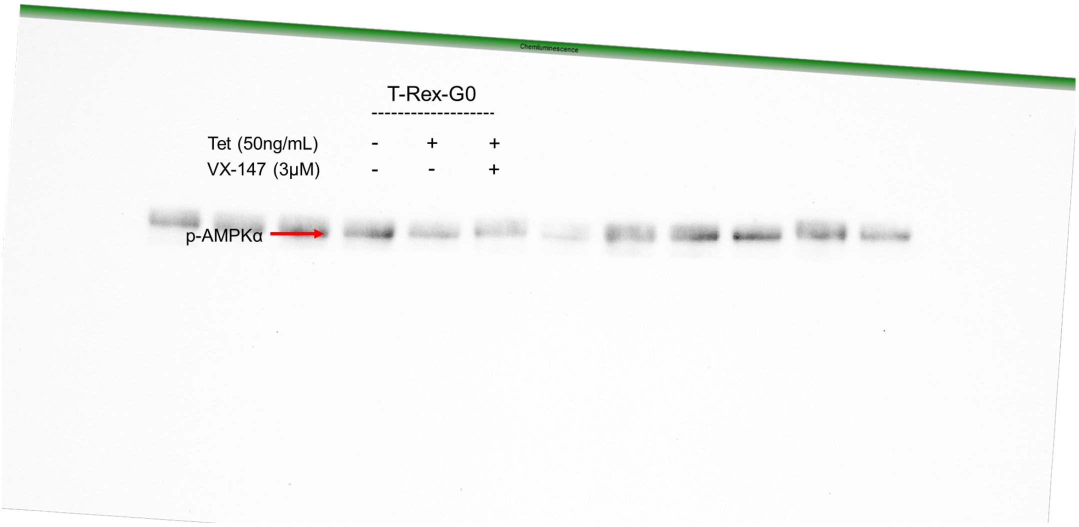

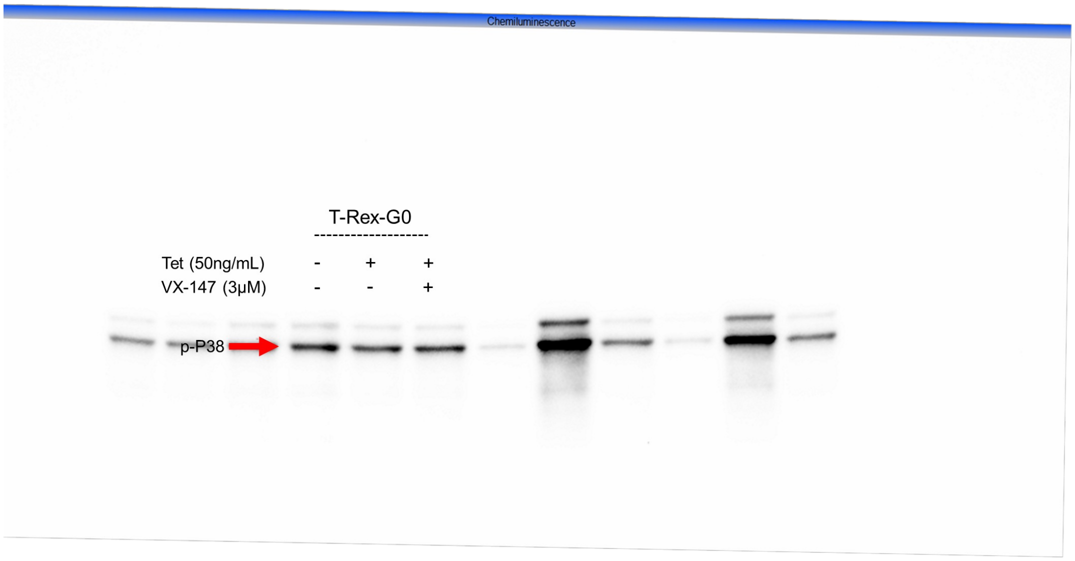

Full unedited gel for Supplementary Figure 7D.

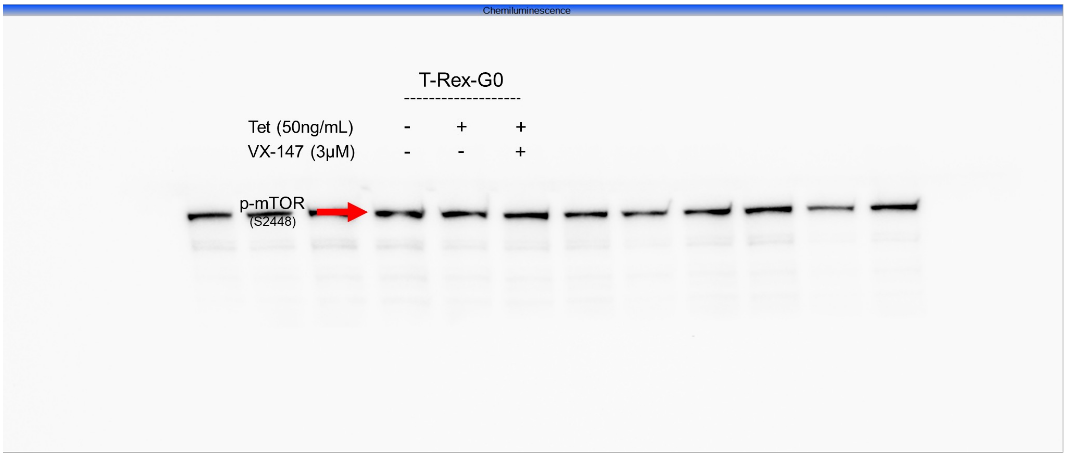

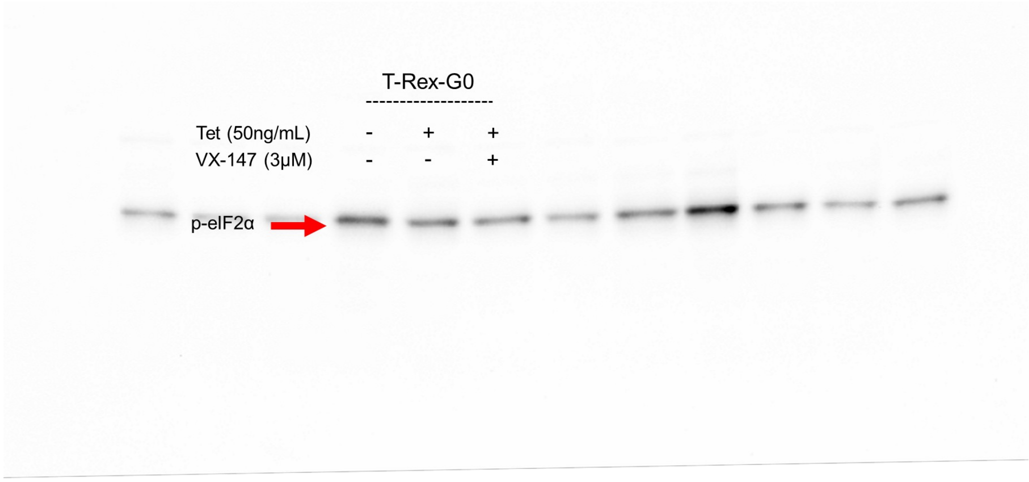

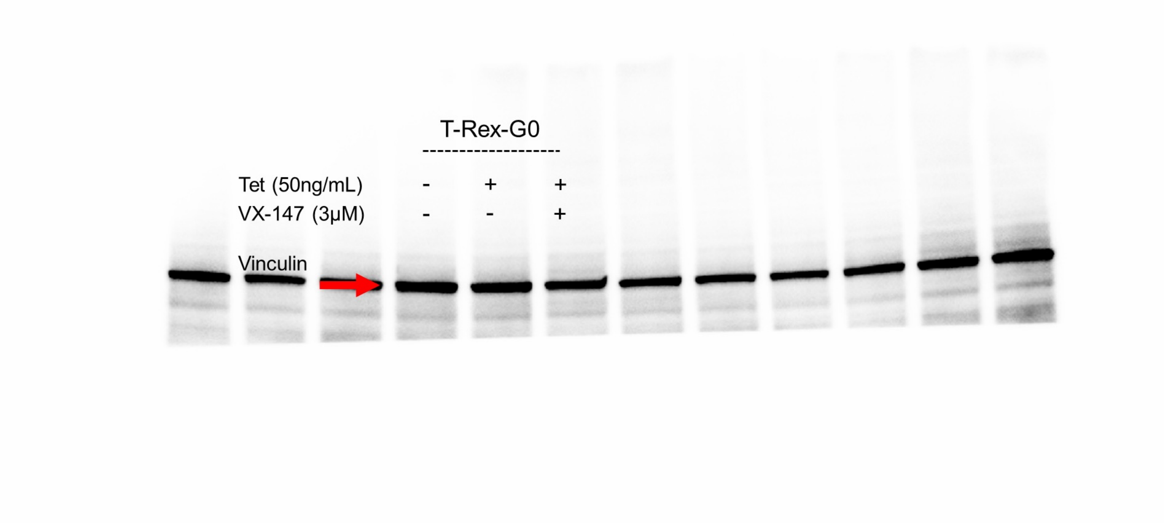

Full unedited gel for Supplementary Figure 9D.

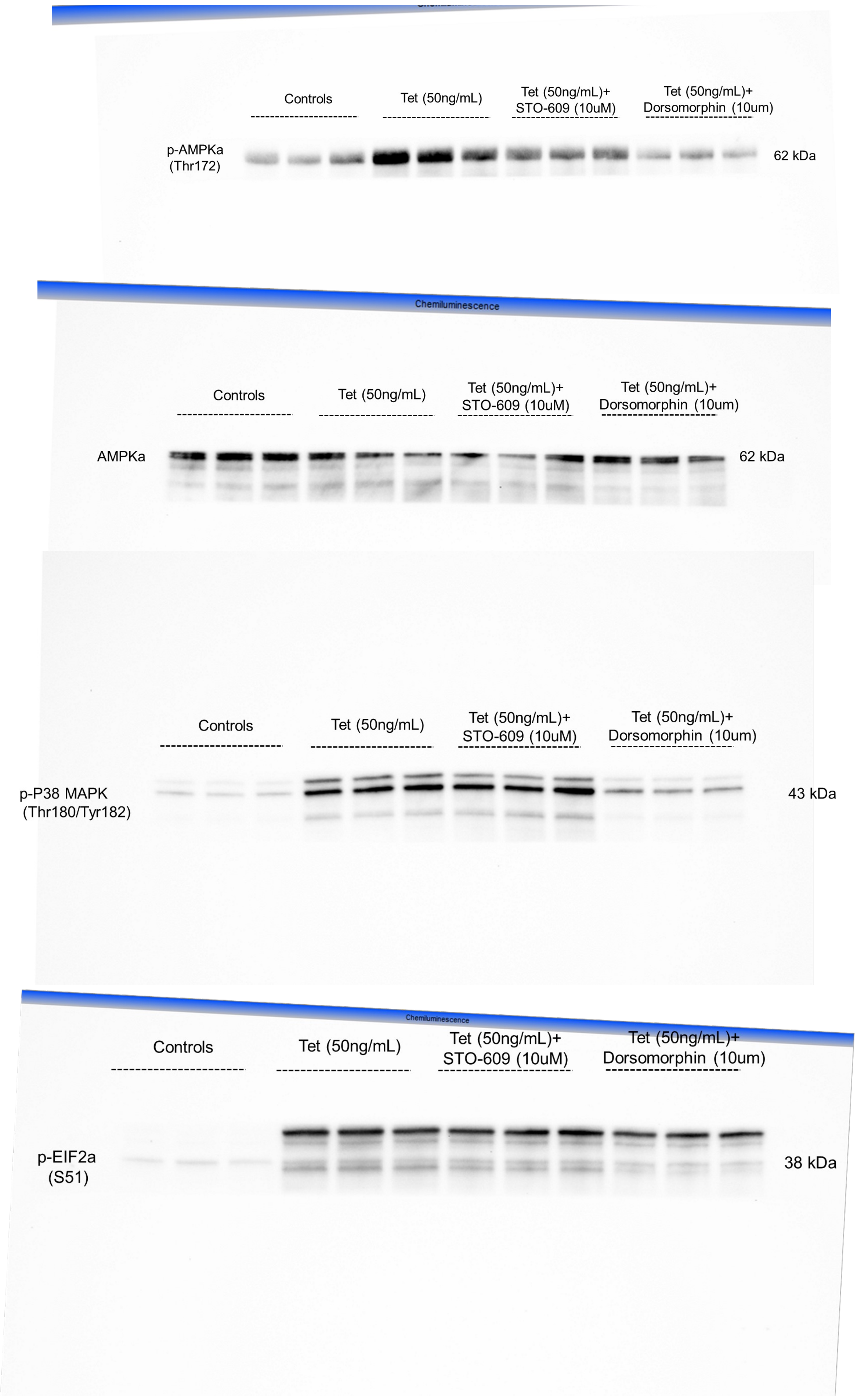

Full unedited gel for Supplementary Figure 9D.

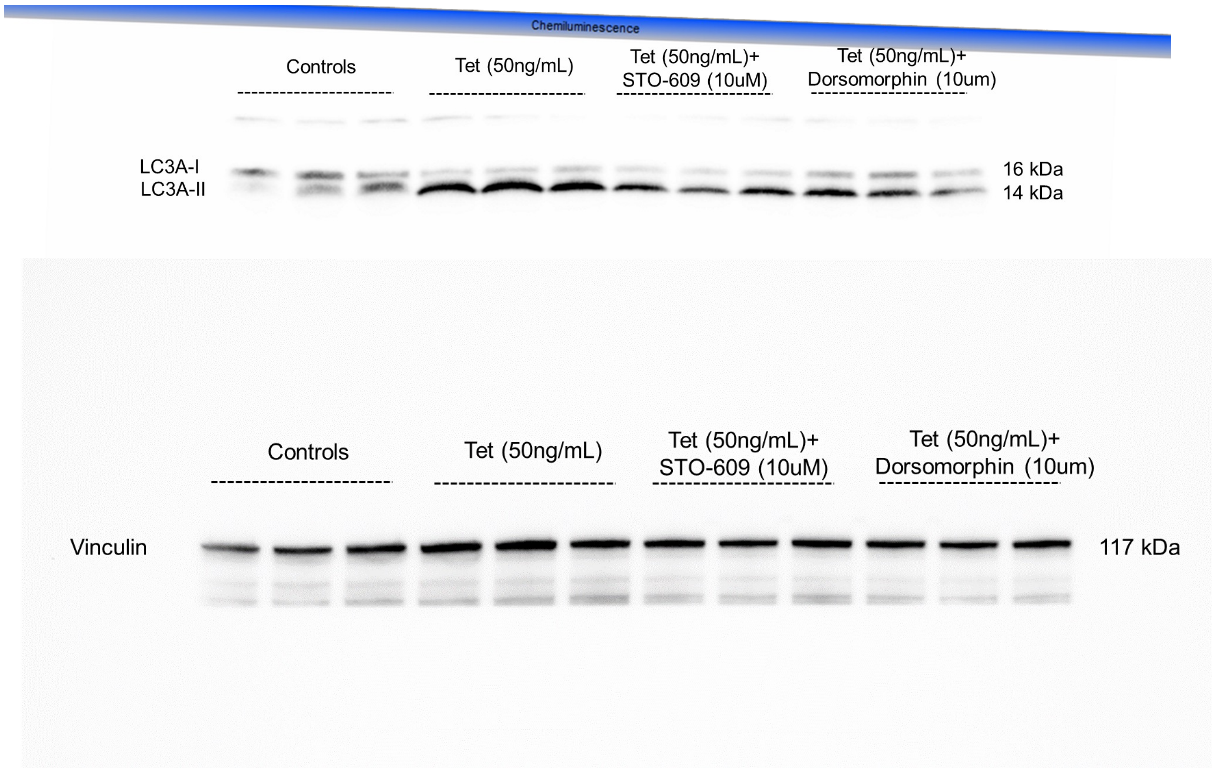

Full unedited gel for Supplementary Figure 9E.

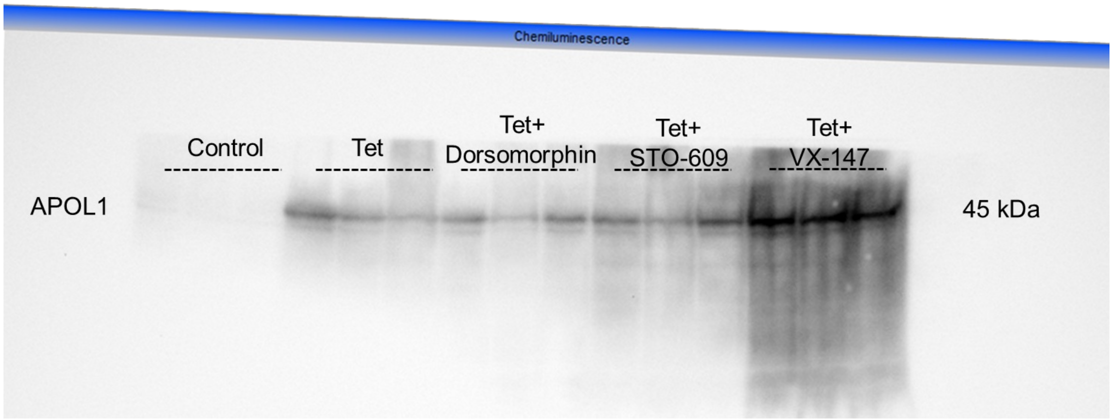

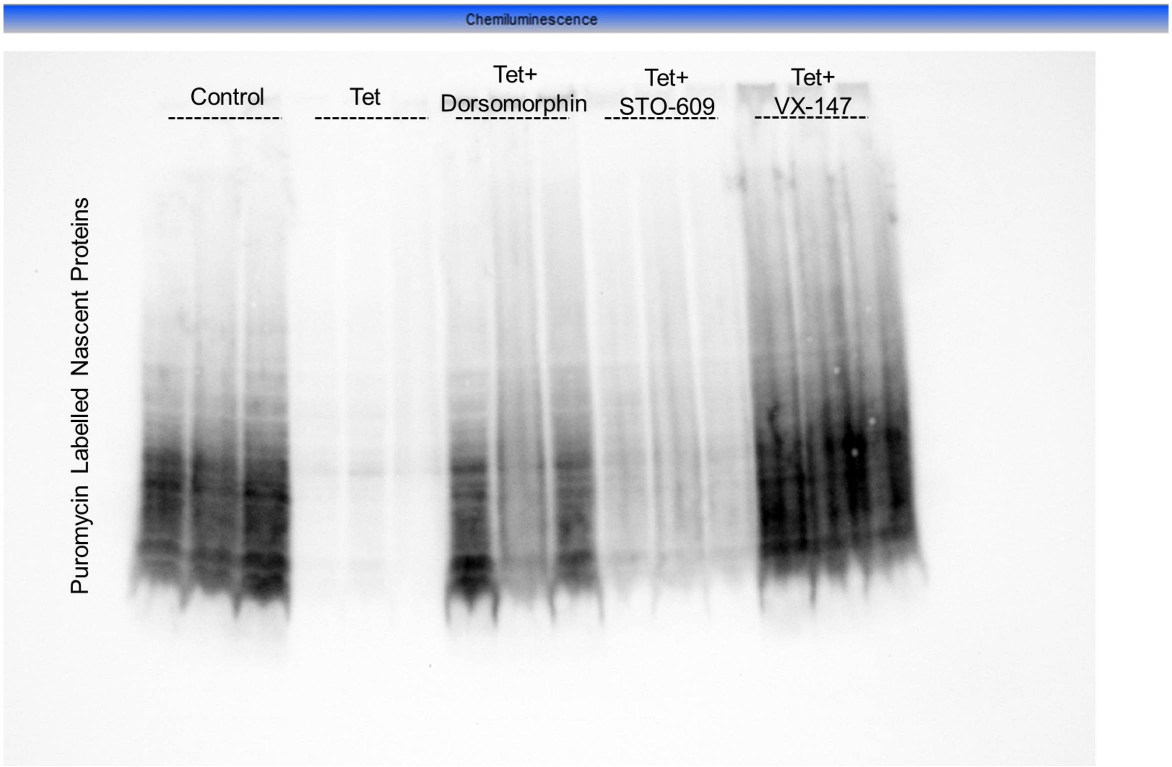

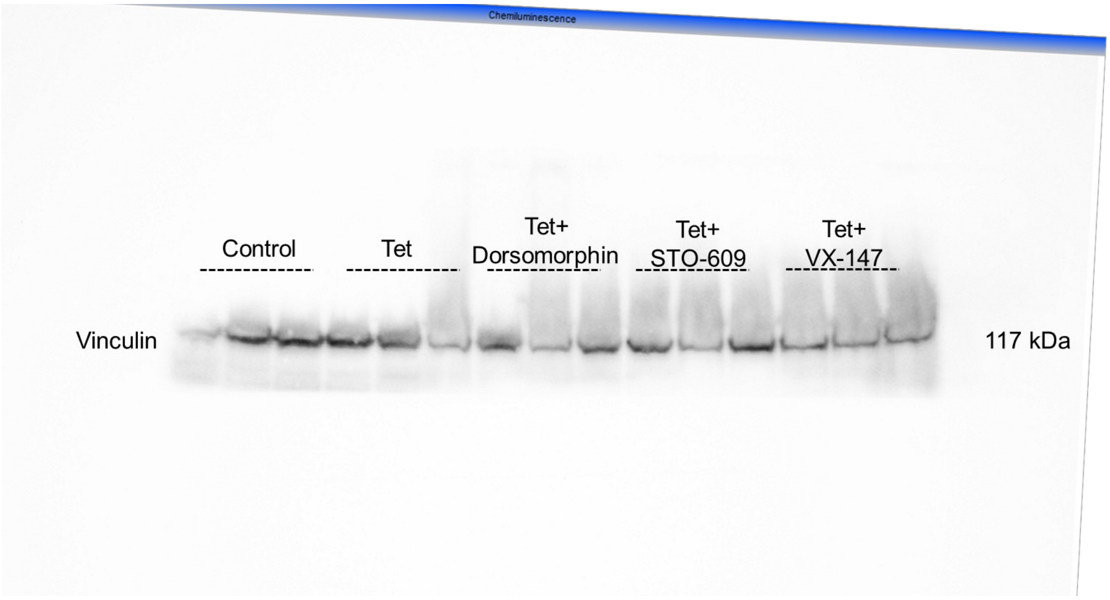

Full unedited gel for Supplementary Figure 9G.

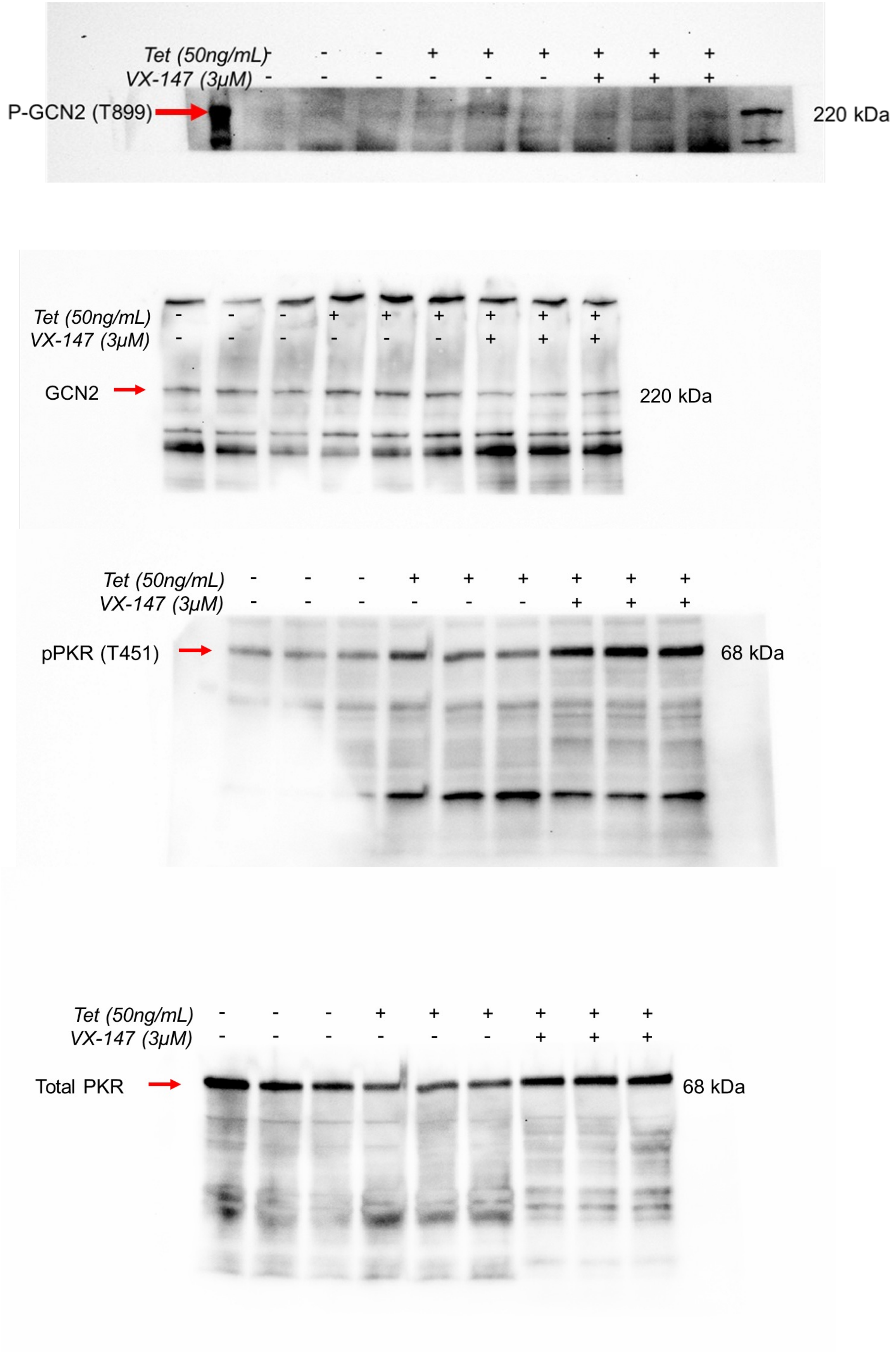

Full unedited gel for Supplementary Figure 9G.

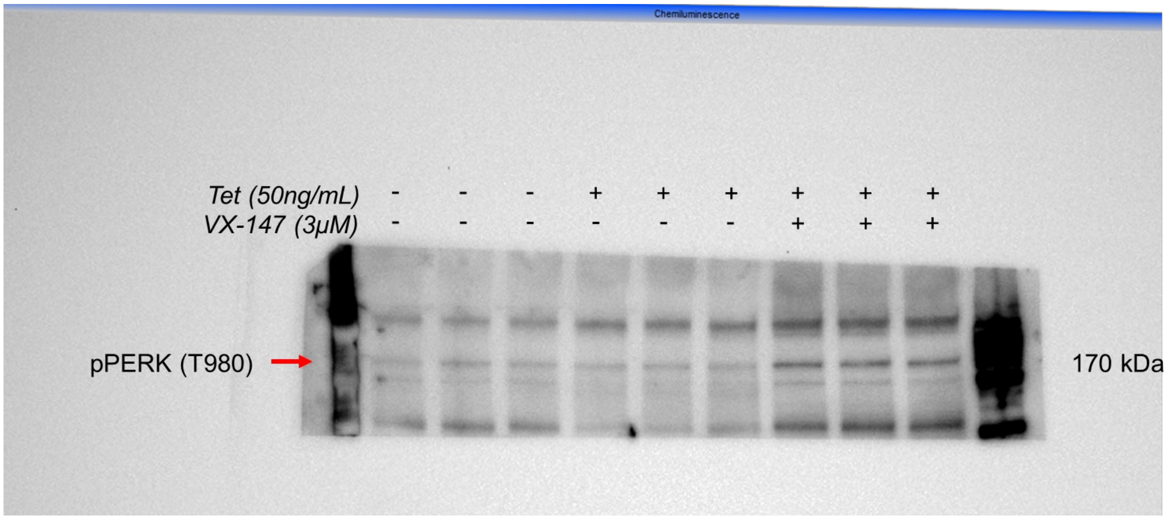

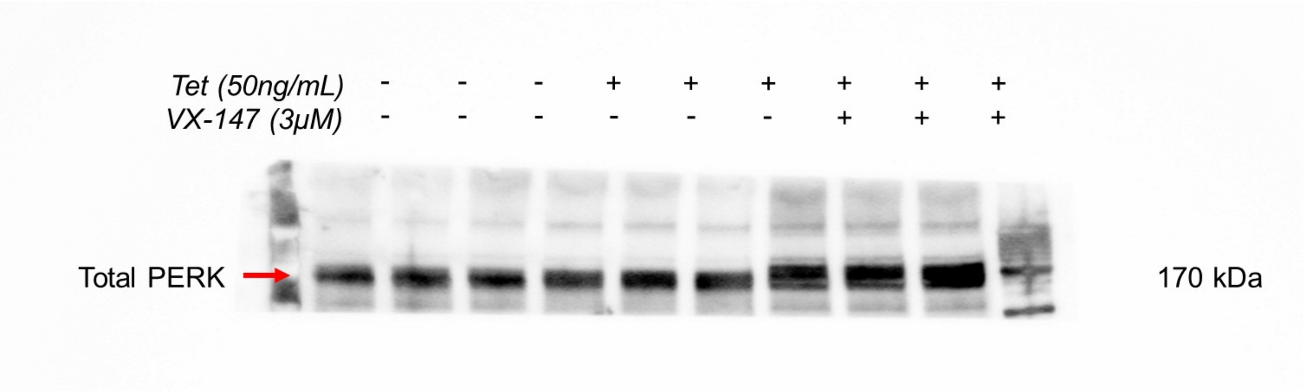

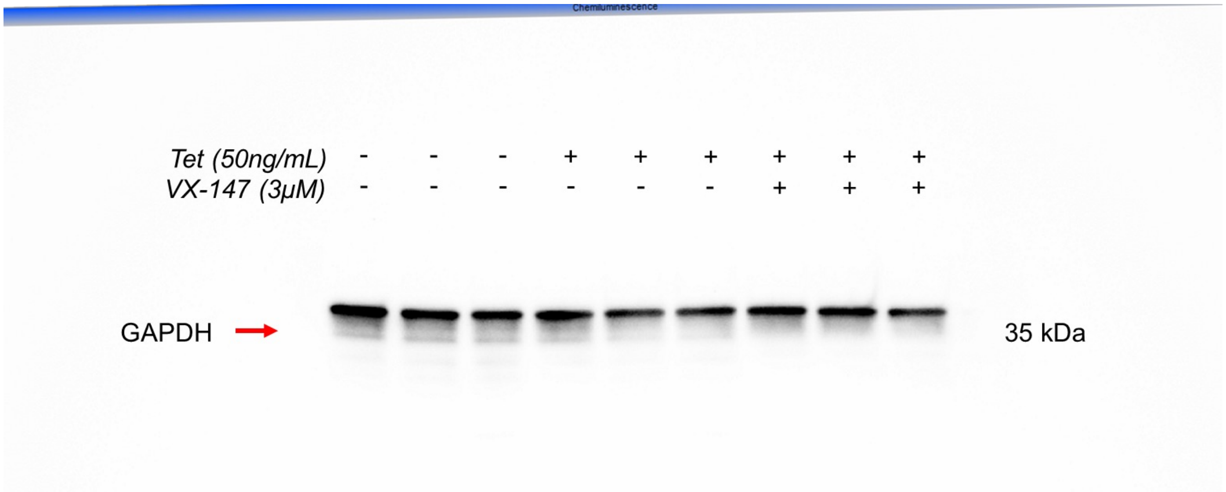

Supplement: Unedited blot and gel images [file jci-134-172262-s222.pdf]
